# Supplementary material for: Dispersal-induced instability in complex ecosystems
Source: Nat Commun. 2020 Nov 27;11:6032. doi: 10.1038/s41467-020-19824-4 (PMC7695839; doi:10.1038/s41467-020-19824-4)
Supplement: Supplementary file 1 — Supplementary Information [file 41467_2020_19824_MOESM1_ESM.pdf]

Dispersal-induced instability in complex ecosystems

— Supplementary Information —

Joseph W. Baron[1,2,*] and Tobias Galla[1,2,†]

[1]*Department of Physics and Astronomy, School of Natural Sciences, The University of Manchester, Manchester M13 9PL, United Kingdom*

[2]*Instituto de Física Interdisciplinar y Sistemas Complejos IFISC (CSIC-UIB), 07122 Palma de Mallorca, Spain*

CONTENTS

I Further details of model setup and analysis S4

S1. Model construction S4
- A. General setup: trophic structure, species interactions and diffusive dispersal S4
- B. Fourier transform and stability matrix S5
- C. Correspondence with a model of a meta-ecosystem with patches S7

S2. Boundary of the bulk of the eigenvalue distribution S8
- A. Overview: approach to calculating the eigenvalue spectral density S8
- B. Preliminaries – setting up the calculation S9
- C. Carrying out the average over the ensemble of random matrices S11

S3. Outlier eigenvalues S17
- A. Setup and general relations S17
- B. Simplification to an effective 2-species problem S17
- C. Proof that the resolvent matrix is diagonal S19
- D. Final expression for outliers S19

S4. Scaling with N and connectance C S20
- A. Rescaling with N S21
- B. Taking into account connectance C S21
- C. Combined effect S23

S5. Stability criteria S23
- A. Instabilities of the non-spatial system S24
- B. Dispersal-induced instability S27
- C. Dispersal alone cannot make an unstable equilibrium stable S29

S6. Parameters used for figures in the main text S29

* josephbaron@ifisc.uib-csic.es

† tobias.galla@manchester.ac.uk

II Robustness against variations of the model S31

S7. Universality of the theory – non-Gaussian random matrices S31
- A. Analytical demonstration of universality S31
- B. Verification with Bernoulli, Laplace and uniformly distributed random numbers S32

S8. Strict enforcement of trophic interactions S33

S9. Variation in diagonal elements S36
- A. Boundary of the bulk of the eigenvalue spectrum S36
- B. Diagonal variation stretches the bulk support along the real axis S39
- C. Calculation of outlier eigenvalues S40
- D. Dispersal-induced instability remains possible in models with trophic structure when self-interaction is heterogeneous S42
- E. Variation in dispersal coefficients can promote dispersal-induced instability S42

III Turing instability and the stabilising effects of spatial heterogeneity S44

S10. Spatial heterogeneity in interaction coefficients S45
- A. Model setup S45
- B. Saddle-point calculation for the bulk spectrum S46
- C. Outlier eigenvalues S49

S11. Previous results as limiting cases S51
- A. Model in the main text S51
- B. Stabilising mechanism reported by Gravel, Massol and Leibold (‘GML’) [S1] S52

S12. Additions to the model by GML S56
- A. The stabilising mechanism is preserved when dispersal is local S57
- B. Inclusion of correlations: $\Gamma \neq 0$ S59
- C. Outlier eigenvalues are not necessarily subject to the same stabilising effect as the bulk S60

S13. Dispersal-induced instability persists on a discrete-patch landscape with all-to-all dispersal S61

S14. Combining trophic structure, interaction heterogeneity and dispersal S63
- A. Stabilising and destabilising mechanisms: explicit example S63
- B. Analytical demonstration S64
- C. Coexistence of the Turing instability and the heterogeneity-associated stabilisation S65
- D. What determines if dispersal is stabilising or destabilising? S66

References S70

OVERVIEW

This Supplementary Material is structured as follows:

In Part I (Sections S1 – S6) we provide further details of the model setup and of the analysis. In particular we describe the calculation of the bulk region of the spectrum and of the outlier eigenvalues. We then discuss how this can be used to obtain criteria for stability. Most importantly, we show how one can use our results for the outlier eigenvalues to deduce parameter sets for which there is a dispersal-induced instability. We also provide the values of the model parameters used for the figures in the main text.

In Part II (Sections S7 – S9) we test the robustness of our main result – the possibility of dispersal-induced instability – against a number of possible variations of the model. For example, we comment on universality (i.e., non-Gaussian interaction matrices), the effects of strictly enforced trophic interactions, and of heterogeneity in the diffusion or self-interaction coefficients.

In Part III (Sections S10 – S14) we connect with the work in Ref. [S1] who reported that dispersal, in combination with spatial heterogeneity, can stabilise equilibria of complex ecosystems. To this end, we include the effects of spatial heterogeneity in our model with trophic structure. This broader model encompasses the set-up in [S1] and that in our main paper as limiting cases. We show that both the stabilising and destabilising effects of dispersal may be observed, depending on the circumstances. Using our analytical approach to calculate bulk spectra and outlier eigenvalues, we identify the factors which can make dispersal a stabilising or a destabilising influence.

Part I

Further details of model setup and analysis

This part of the Supplementary Material contains further details relating to the construction of the model (Section S1), followed by the calculation of the bulk eigenvalue spectrum in Section S2, and of outlier eigenvalues in Section S3. In Section S4 we comment on further technical details used to carry out the random-matrix calculation, and on how a connectance parameter C can be accounted for. In Section S5 we show how these results can be used to obtain stability criteria for equilibria of the ecosystem. Section S6 finally provides the detailed model parameters used for the figures in the main manuscript.

S1. MODEL CONSTRUCTION

A. General setup: trophic structure, species interactions and diffusive dispersal

We suppose that the total community consists of a sub-community of predator species and a sub-community of prey species, which are distributed in space. We assume that there are $N_u = \gamma_u N$ different prey species and $N_v = \gamma_v N$ predator species, where $\gamma_u + \gamma_v = 1$. N is the total number of species such that $N = N_u + N_v$.

We imagine that the system reaches an equilibrium and that the equations governing the dynamics of the species abundances, linearised about this equilibrium, take the following form

$$\frac{\partial u_i}{\partial t} = D_u \frac{\partial^2 u_i}{\partial x^2} - d_u u_i + \sum_{k=1}^{N_u} A_{ik}^{uu} u_k + \sum_{j=1}^{N_v} A_{ij}^{uv} v_j,$$

$$\frac{\partial v_j}{\partial t} = D_v \frac{\partial^2 v_j}{\partial x^2} - d_v v_j + \sum_{i=1}^{N_u} A_{ji}^{vu} u_i + \sum_{k=1}^{N_v} A_{jk}^{vv} v_k. \tag{S1}$$

The $u_i(x,t)$, $i = 1, \ldots, N_u$, describe perturbations of the abundances of the prey species about the equilibrium at position x and time t, and similarly $v_j(x,t)$, $j = 1, \ldots, N_v$, are perturbations of the predator abundances.

We presume for now that the interactions within a single-species population are competitive (such that $d_u > 0$ and $d_v > 0$) and that each species disperses via diffusion. The corresponding dispersal coefficients are D_u for prey, and D_v for predators. Crucially, the prey and predator species can have different dispersal coefficients $D_u \neq D_v$. In the model there are also inter-population interactions. These are described by the interaction coefficients $A_{ij}^{\alpha\beta}$, where α and β can takes values $\alpha, \beta \in \{u, v\}$, where u stands for prey and v for predator. For example, A_{ij}^{uu} describes interactions between different prey species, and A_{ij}^{uv} describes the effect that predator species j has on prey species i.

The interaction coefficients $A_{ij}^{\alpha\beta}$ are random numbers drawn from a Gaussian distribution (although this assumption can be relaxed – see Section S7) with

$$\overline{A_{ij}^{\alpha\beta}} = \mu_{\alpha\beta},$$

$$\overline{(A_{ij}^{\alpha\beta} - \mu_{\alpha\beta})^2} = \sigma^2,$$
$$\overline{(A_{ij}^{\alpha\beta} - \mu_{\alpha\beta})(A_{ji}^{\alpha'\beta'} - \mu_{\alpha'\beta'})} = \Gamma_{\alpha\beta\alpha'\beta'}\sigma^2. \tag{S2}$$

All other correlations are zero. We assume that the only non-zero $\Gamma_{\alpha\beta\alpha'\beta'}$ are $\Gamma_{uuuu} \equiv \Gamma_u$, $\Gamma_{uvvu} \equiv \Gamma_{uv}$ and $\Gamma_{vvvv} \equiv \Gamma_v$. With a probability $1 - C$, species i and j are chosen *not* to interact with one another and thus $A_{ij}^{\alpha\beta}$ and $A_{ji}^{\beta\alpha}$ are set to zero. The parameter C therefore describes the 'connectance' (the probability that any given pair of species interact with one another).

Each of the model parameters can be interpreted ecologically. We define and interpret the complexity $c = CN\sigma^2$ in the main text. The interaction mean μ_{uu} indicates the degree to which two different prey species cooperate on average (if μ_{uu} is positive) or compete (if μ_{uu} is negative). The coefficient μ_{vv} has a similar interpretation for predators. The means of the off-diagonal blocks $\mu_{uv} < 0$ and $\mu_{vu} > 0$ indicate the degree to which (on average) prey species suffer and predator species gain from predator-prey interactions. The coefficients $\Gamma_{\alpha\beta\alpha'\beta'}$ indicate statistical correlations between interaction coefficients. A positive value of $\Gamma_{\alpha\beta\alpha'\beta'}$ indicates a statistical tendency for one species to benefit more (than average) from an interaction if the other species involved does so as well. The opposite is true if $\Gamma_{\alpha\beta\alpha'\beta'}$ is negative. The only non-zero entries are taken to be $\Gamma_u \equiv \Gamma_{uuuu}$, $\Gamma_v \equiv \Gamma_{vvvv}$ and $\Gamma_{uv} \equiv \Gamma_{uvvu}$. That is, only elements which are diagonally opposite one another in $\mathbf{A}$ are correlated [S2, S3].

Models of complex ecosystems with structured random matrices have been considered before. For example, the work by Grilli et. al. [S4] involves matrices with similar block structure to those in our model, the blocks in [S4] are associated with plant and animal species. Ref. [S5] evaluates the eigenvalue spectra of random matrices with cascade structure. The elements above and below the diagonal respectively have different means and variances to the elements below the diagonal. However, this is different from the structure of the matrices in our model. We are not aware of any work combining both block structure and dispersal, and computing the eigenvalue spectra of such models.

B. Fourier transform and stability matrix

The perturbations $u_i(x,t)$ and $v_j(x,t)$ in Eq. (S1) are functions of position (and time). Taking the Fourier transform with respect to the spatial coordinate x, we write

$$\tilde{u}_i(q) = \frac{1}{\sqrt{2\pi}} \int dx\; e^{iqx} u_i(x), \tag{S3}$$

and similar for $\tilde{v}_j(q)$. The $\tilde{u}_i(q), \tilde{v}_j(q)$ describe perturbations of wavenumber q, or equivalently, of wavelength $\lambda = 2\pi/q$.

Using this in Eqs. (S1) one obtains

$$\dot{\tilde{u}}_i = -(q^2 D_u + d_u)\tilde{u}_i + \sum_{k=1}^{N_u} A_{ik}^{uu}\tilde{u}_k + \sum_{j=1}^{N_v} A_{ij}^{uv}\tilde{v}_j,$$
$$\dot{\tilde{v}}_j = -(q^2 D_v + d_v)\tilde{v}_j + \sum_{i=1}^{N_u} A_{ji}^{vu}\tilde{u}_i + \sum_{k=1}^{N_v} A_{jk}^{vv}\tilde{v}_k. \tag{S4}$$

We note that by setting $q = 0$ in Eqs. (S4) one recovers Eqs. (S1) with the diffusion term removed. From the definition of the Fourier transform Eq. (S3), we see these are then equations for the total u_i and v_j summed throughout space. This removes any sense of spatial distribution of species. Setting $q = 0$ therefore recovers the naïve 'non-spatial' model where species dispersal is not taken into account. Eqs. (S4) can be written in a more compact matrix form

$$\dot{\tilde{X}} = \mathbf{M}_q \tilde{X}, \tag{S5}$$

where the vector X is of length N, and with

$$\mathbf{M}_q = -q^2 \mathbf{D} - \mathbf{d} + \mathbf{A}. \tag{S6}$$

The matrix $\mathbf{M}_q$ is of dimension $N \times N$. It is the sum of three terms:

(i) The first term is $-q^2\mathbf{D}$, where the matrix $\mathbf{D}$ is of size $N \times N$, diagonal and of the form

$$\mathbf{D} = \begin{pmatrix} D_u \mathbb{1}_{N_u} & 0 \\ 0 & D_v \mathbb{1}_{N_v} \end{pmatrix}, \tag{S7}$$

where $\mathbb{1}_{N_u}$ and $\mathbb{1}_{N_v}$ are identity matrices of dimensions $N_u \times N_u$ and $N_v \times N_v$ respectively.
(ii) The second term is of a similar form

$$\mathbf{d} = \begin{pmatrix} d_u \mathbb{1}_{N_u} & 0 \\ 0 & d_v \mathbb{1}_{N_v} \end{pmatrix}, \tag{S8}$$

and describes intraspecific interaction.
(iii) The third term in $\mathbf{M}_q$ is a random matrix with the Gaussian statistics given in Eq. (S2). We note that the matrix $\mathbf{M}_q$ has a block structure; it is comprised of submatrices of dimensions $N_u \times N_u$, $N_u \times N_v$, $N_v \times N_u$ and $N_v \times N_v$. This is illustrated in Fig. 1 of the main text.

The eigenvalues of the matrix $\mathbf{M}_q$ tell us about the stability of the hypothetical fixed point about which we have linearised. More precisely, the fixed point is stable against perturbations of wavelength $\lambda = 2\pi/q$ if and only if all eigenvalues of $\mathbf{M}_q$ have negative real part. The fixed point is stable against perturbations of *any* wavelength only if this is the case for *all* q. That is to say, in order for the equilibrium to be stable, the matrix $\mathbf{M}_q$ must not have an eigenvalue with positive real part for any q.

We therefore wish to identify the eigenvalue spectrum of $\mathbf{M}_q$. We can then use this to deduce under what conditions the equilibrium is stable. The matrix $\mathbf{M}_q$ has $N_u + N_v$ eigenvalues in total. The eigenvalue spectrum consists of a 'bulk' of eigenvalues with some possible outliers (see Fig. 2 in the main text). The bulk contains the vast majority of eigenvalues. They are confined to a compact area in the complex plane. Both the boundary of the bulk region and the location of the outliers can be calculated based only on the statistical properties of the matrix $\mathbf{M}_q$ (as opposed to the exact entries). Further, the boundary of the bulk region can be calculated independently of the outliers. We therefore split our calculation into two parts, and present the calculation of bulk eigenvalues in Section S2, and that for the outliers in Section S3.

C. Correspondence with a model of a meta-ecosystem with patches

We now briefly consider a model in which species hop between discrete patches, as opposed to diffusion in a continuous space. A more complete treatment of a model with discrete patches (with the possibility of statistical difference between the patches) is given in Section S10.

To do this we imagine each species to be located on one of a set of discrete patches, whose locations are given by x. The continuous diffusion term in Eqs. (S1) is then replaced by its discrete counterpart to yield

$$\begin{aligned}\frac{\partial u_i x)}{\partial t} &= \hat{D}_u \sum_{x'} \left[\phi(x,x')u_i(x') - \phi(x',x)u_i(x)\right] - d_u u_i(x) \\ &\quad + \sum_{k=1}^{N_u} A_{ik}^{uu} u_k(x) + \sum_{j=1}^{N_v} A_{ij}^{uv} v_j(x), \\ \frac{\partial v_j(x)}{\partial t} &= \hat{D}_v \sum_{x'} \left[\phi(x,x')v_j(x') - \phi(x',x)v_j(x)\right] - d_v v_j(x) \\ &\quad + \sum_{i=1}^{N_u} A_{ji}^{vu} u_i(x) + \sum_{k=1}^{N_v} A_{jk}^{vv} v_j(x). \end{aligned} \tag{S9}$$

We have suppressed the dependence on time to keep the notation compact. The quantity $\phi(x, x')$ is a 'dispersal kernel', describing the probability that an individual hops from patch x' to patch x, given that a hop occurs. We assume that the patches form a linear chain, and we suppose that dispersal is only between neighbouring patches and occurs with equal probability to either side. We therefore choose

$$\phi(x,x') = \begin{cases} \frac{1}{2} & \text{if} \quad x = x' + \Delta x, \\ \frac{1}{2} & \text{if} \quad x = x' - \Delta x, \\ 0 & \text{otherwise,} \end{cases} \tag{S10}$$

where Δx is the inter-patch separation. The constants $\hat{D}_u$ and $\hat{D}_v$ in Eqs. (S9) describe the overall dispersal rates (for prey and predators respectively) to any of the two neighbouring patches.

Next, we take the discrete Fourier transform with respect to the spatial variable in Eqs. (S9) ,

$$\tilde{u}_i(q) = \sum_x \, e^{iqx} u_i(x). \tag{S11}$$

We obtain

$$\begin{aligned}\frac{\partial \tilde{u}_i}{\partial t} &= \hat{D}_u[\tilde{\phi}(q) - 1]\tilde{u}_i - d_u \tilde{u}_i + \sum_{k=1}^{N_u} A_{ik}^{uu} \tilde{u}_k + \sum_{j=1}^{N_v} A_{ij}^{uv} \tilde{v}_j \\ \frac{\partial \tilde{v}_j}{\partial t} &= \hat{D}_v[\tilde{\phi}(q) - 1]\tilde{v}_j - d_v \tilde{v}_j + \sum_{i=1}^{N_u} A_{ji}^{vu} \tilde{u}_i + \sum_{k=1}^{N_v} A_{jk}^{vv} \tilde{v}_j, \end{aligned} \tag{S12}$$

with

$$\tilde{\phi}(q) = \cos\left(q\Delta x\right). \tag{S13}$$

Provided the arrangement of patches is 'dense', i.e., that there are many patches per unit distance, we can assume $\Delta x \ll 1$, and expand $\tilde{\phi}(x) = \cos(q\Delta x)$ in powers of Δx. To lowest non-trivial order we find

$$\tilde{\phi}(x) \approx 1 - \frac{1}{2}(q\Delta x)^2. \tag{S14}$$

Eqs. (S12) then become

$$\begin{aligned}\frac{\partial \tilde{u}_i}{\partial t} &= -(q^2 D_u + d_u)\tilde{u}_i + \sum_{k=1}^{N_u} A^{uu}_{ik}\tilde{u}_k + \sum_{j=1}^{N_v} A^{uv}_{ij}\tilde{v}_j,\\ \frac{\partial \tilde{v}_j}{\partial t} &= -(q^2 D_v + d_v)\tilde{v}_j + \sum_{i=1}^{N_u} A^{vu}_{ji}\tilde{u}_i + \sum_{k=1}^{N_v} A^{vv}_{jk}\tilde{v}_k,\end{aligned} \tag{S15}$$

where we have identified the diffusion constants D_α in terms of the hopping rates $\hat{D}_\alpha$: $D_\alpha = \frac{\hat{D}_\alpha(\Delta x)^2}{2}$. We thus arrive at Eqs. (S1).

This shows that any stability criteria derived for the ecosystem with diffusion in continuous space also applies to a model with discrete patches and dispersal between adjacent patches. This is valid provided the distance between two patches in space is small compared to the characteristic wavelengths associated with the instability. It is also possible to analyse the model with discrete patches, and to find the eigenvalues as a function of q without performing this approximation. One would still find the possibility for Turing instability (as demonstrated for a different system in [S6] and also in Section S13).

S2. BOUNDARY OF THE BULK OF THE EIGENVALUE DISTRIBUTION

A. Overview: approach to calculating the eigenvalue spectral density

Our approach to calculating the support of the eigenvalue spectra of random matrices is based on methods from statistical physics. The study of random matrices has a long history in physics, going to back to Wigner who first established the semi-circle law for symmetric Gaussian random matrices in the context of nuclear physics [S7, S8]. There is a wide range of other applications of random matrices in physics [S9], in particular also in the theory of spin glasses and disordered systems [S10].

In this context, a number of different theoretical approaches have been developed to evaluate the spectra of random matrices (see e.g. [S11]). For example, one can recognise the resolvent of the random matrix [defined below in Eq. (S16)] as the Stieltjes transform of the eigenvalue density in the limit of a large size N of the matrix. This was done for Hermitian matrices in the seminal work by Pastur [S12]. This method has since been developed further – the case of non-Hermitian matrices can be addressed through the use of quaternionic resolvents [S13, S14]. Alternatively, one can evaluate the resolvent using a series expansion via the Dyson equation, dropping terms of sub-leading order in N in the limit $N \to \infty$, as was done by Bray and Moore [S15]. We use elements of this argument in Section S3 C. Approaches involving series expansions of the resolvent have also been extended to take into account higher-order correlations [S16]. We note that the

characterisation of spectra of random matrices is also a timely topic in applied mathematics. Recent work includes [S17, S18], see also [S19] for a further overview.

Our analytical approach to the calculation of bulk eigenvalue spectra in this section broadly proceeds along the lines of [S20], who used the replica method to compute elliptic spectra of random matrices with correlations between elements, but with no particular sub-structure or compartments in the matrix. The approach of [S20] is based on the introduction of a suitable 'potential' Φ (to be defined below), which is averaged over the ensemble of random matrices, and from which the resolvent can then be obtained. Essentially, the calculation converts the problem of finding the bulk region of eigenvalues into one of evaluating an integral over what would be called the (macroscopic) 'order parameters' in statistical mechanics. In the limit of large matrices, this integral is then carried out using the saddle-point approximation (see e.g. [S10, S21, S22] for modern accounts and applications to problems in disordered systems). The saddle-point method is a common approach in the theory of disordered system, with wide-ranging applications in spin-glass physics [S10], neural networks [S23, S24] and interacting-agent models and game theory [S22, S25]. Work using this approach to calculate spectra of random matrices includes [S26], see also [S11] for a more comprehensive overview.

In Section S3 we then combine this calculation of the bulk region of the eigenvalue spectrum with the approach of Ref. [S27] to find the outlier eigenvalues. These arise from the introduction of a non-zero mean to the random matrix elements. The resolvents necessary for the calculation of these outliers can readily be identified from the order parameters in the saddle-point calculation of the bulk spectrum.

The primary methodological contribution of our work is the calculation of the eigenvalue support for matrices with block structure. The block structure to which we refer is defined in more detail in the main paper and in S1 of this Supplement. In the analysis, the block structure is dealt with conveniently through the introduction of integration variables with an additional block index α. In principle, this approach can be extended for matrices with more complicated structure and statistics (Parts II and III of this Supplement contain examples). The approach has further advantages, for instance, the origin of universality is quite transparent (see S7).

B. Preliminaries – setting up the calculation

For an ensemble of $N \times N$ random matrices $\mathbf{m}$, consider the so-called resolvent, defined by

$$G(\omega) = \frac{1}{N} \overline{\mathrm{Tr}\left[(\omega \mathbb{1}_N - \mathbf{m})^{-1}\right]}. \tag{S16}$$

The overbar represents an average over the ensemble of matrices. The expression $\mathbb{1}_N$ denotes the identity matrix of size $N \times N$.

We note the following identities from general linear algebra (for an $N \times N$ matrix $\mathbf{B}$):

(i) $\mathrm{Tr}\left[\mathbf{P}^{-1}\,\mathbf{B}\,\mathbf{P}\right] = \mathrm{Tr}\left[\mathbf{B}\right]$, for any invertible $N \times N$ matrix $\mathbf{P}$;

(ii) If $\mathbf{B}$ is invertible, then the eigenvalues of $\mathbf{B}^{-1}$ are reciprocals of those of $\mathbf{B}$;

(iii) The trace is the sum of the eigenvalues;

(iv) Adding the identity matrix to a matrix increases each eigenvalue of this matrix by 1;

(v) Multiplying a matrix by a constant scales its eigenvalues by that same constant.

Using these, one can show that

$$G\left(\omega\right) = \frac{1}{N}\overline{\sum_{\lambda} \frac{1}{\omega - \lambda}}, \tag{S17}$$

where the λ are the eigenvalues of $\mathbf{m}$. In the limit $N \to \infty$, this sum can be written as an integral over the complex plane

$$G\left(\omega\right) = \int d^2\lambda \frac{\rho(\lambda)}{\omega - \lambda}, \tag{S18}$$

where $\rho(\lambda)$ is the density of eigenvalues.

Bearing in mind that $G(\omega)$ is a complex quantity, we perform a contour integral around a closed path $\mathcal{C}$, which we presume contains none of the poles of G,

$$\begin{aligned} \frac{1}{2\pi i}\int_{\mathcal{C}} d\omega\ G(\omega) &= \frac{1}{N}\overline{\sum_{\lambda} \frac{1}{2\pi i}\int_{\mathcal{C}} d\omega \frac{1}{\omega - \lambda}} \\ &= \frac{1}{N}\overline{\sum_{\lambda} 1} = \int_{\mathcal{S}} d^2\lambda\ \rho(\lambda), \end{aligned} \tag{S19}$$

In this expression $\mathcal{S}$ denotes the region bounded by $\mathcal{C}$, and we have used the residue theorem to evaluate the contour integral.

We next note the following complex version of Gauss' law (letting $\omega = x + iy$)

$$\int_{\mathcal{S}} d^2\omega \left[\frac{\partial G}{\partial x} + i\frac{\partial G}{\partial y}\right] = \frac{1}{i}\int_{\mathcal{C}} d\omega\, G(\omega). \tag{S20}$$

Defining the real-valued potential $\Phi(\omega)$ via

$$2\,\mathrm{Re}\, G = -\frac{\partial \Phi}{\partial x}, \quad 2\,\mathrm{Im}\, G = \frac{\partial \Phi}{\partial y}, \tag{S21}$$

and using Eqs. (S19) and (S20), along with the fact that the region $\mathcal{S}$ is arbitrary, we obtain Poisson's equation

$$\nabla^2 \Phi = -4\pi\rho. \tag{S22}$$

In analogy with electrostatics, the resolvent $G(\omega)$ can be thought of as being related to the 'electric field' with components $E_x = 2\,\mathrm{Re}\ G$ and $E_y = -2\,\mathrm{Im}\ G$ [S20]. Therefore, we can obtain the distribution of eigenvalues, if we can find the potential Φ.

The potential Φ can be related directly to the matrix $\mathbf{m}$ via

$$\Phi(\omega) = -\frac{1}{N}\overline{\ln\det\left[(\mathbb{1}_N\omega^\star - \mathbf{m}^T)(\mathbb{1}_N\omega - \mathbf{m})\right]}. \tag{S23}$$

This can be seen to agree with Eqs. (S21) and (S17) once one realises that $\det\left(\mathbb{1}_N\omega - \mathbf{m}\right) = \prod_\lambda(\omega - \lambda)$.

We now make the following observations about the resolvent and its relation to the potential Φ and the eigenvalue density ρ. Eq. (S21) can be written more compactly as

$$G(x,y) \equiv -\frac{1}{N}\overline{\mathrm{Tr}\left[\frac{1}{\mathbb{1}_N\omega - \mathbf{m}}\right]} = \frac{\partial\Phi(\omega,\omega^\star)}{\partial\omega}. \tag{S24}$$

Indeed, we also have

$$G^\star(x,y) = \frac{\partial\Phi(\omega,\omega^\star)}{\partial\omega^\star}. \tag{S25}$$

Combining these relations with Eq. (S22), one sees that the resolvent is related to the eigenvalue density by

$$\frac{\partial\mathrm{Re}[G]}{\partial x} - \frac{\partial\mathrm{Im}[G]}{\partial y} = \mathrm{Re}\left[\frac{\partial G(\omega,\omega^\star)}{\partial\omega^\star}\right] = 2\pi\rho. \tag{S26}$$

The left-hand side of this equation is familiar from the Cauchy-Riemann equations for analytic functions. From this we deduce that if $G(x,y)$ is an analytic function in a particular region (i.e. we can write it in terms of ω only), the eigenvalue density must be zero in this region.

C. Carrying out the average over the ensemble of random matrices

Ensemble of random matrices

Adding a low-rank perturbation to a large random matrix is known not to change the bulk of the eigenvalue spectrum [S27–S29]. This means that we can assume that the mean of the distribution from which the matrix elements are drawn is zero. The mean can have an effect on outlier eigenvalues, and we will therefore restore it for the calculation of the outliers in Section S3.

For the time being we consider a Gaussian random matrix with statistics as follows

$$\begin{aligned}
\overline{m_{ij}^{\alpha\beta}} &= 0, \\
\overline{(m_{ij}^{\alpha\beta})^2} &= \frac{\sigma^2}{N}, \\
\overline{m_{ij}^{\alpha\beta} m_{ji}^{\alpha'\beta'}} &= \frac{\Gamma_{\alpha\beta\alpha'\beta'}\sigma^2}{N}, \\
m_{ii}^{\alpha\alpha} &= -d_\alpha,
\end{aligned} \tag{S27}$$

The matrix $\mathbf{m}$ is similar to $\mathbf{M}_q$ (the matrix we focus on in the main paper), but it is different in a number of ways:

First, we note that we have re-scaled the matrix elements relative to the matrix $\mathbf{M}_q$ in the main paper – the variance and co-variances are now proportional to $1/N$. This approach is standard in the theory of random matrices, its objective is of a technical nature. The re-scaling allows us to carry out the calculation in the limit $N \to \infty$. In practice the results derived in this way are often accurate also for finite N. The scaling is undone by replacing $\sigma^2 \to N\sigma^2$ in the result. For a more detailed discussion, see Section S4.

Further, we are assuming all-to-all interaction ($C = 1$) for now between the species. We discuss how choosing a general connectance $0 < C < 1$ affects the result in Section S4.

Finally, the wavenumber q is set to zero here. The result for non-zero q can obtained by making the replacement $d_\alpha \to d_\alpha + q^2 D_\alpha$, see also Section S5 B.

Replica method and Hubbard-Stratononich transformation

In order to calculate the spectrum of the ensemble of random matrices we need to compute $\overline{\ln\det\left[(\omega^* \mathbb{1}_N - \mathbf{m}^T)(\omega \mathbb{1}_N - \mathbf{m})\right]}$. We recall that the overbar stands for the average over the randomness in $\mathbf{m}$. In order to calculate averages of logarithms, one can use the so-called replica method [S10, S20, S29, S30], based on the identity $\ln\ y = \lim_{n\to 0} \frac{y^n - 1}{n}$. This allows one to calculate $\overline{y^n}$ instead of the more intricate average $\overline{\ln\ y}$. The object y^n in turn represents an n-fold 'replicated' system in statistical physics [S10, S20, S29, S30]. In the context of the present problem one finds that the n replicas decouple in the limit of large N, similar to what was observed in [S20, S26]. This means that

$$\overline{\ln\det\left[(\mathbb{1}_N\omega^\star - \mathbf{m}^T)(\mathbb{1}_N\omega - \mathbf{m})\right]} = \ln \overline{\det\left[(\mathbb{1}_N\omega^\star - \mathbf{m}^T)(\mathbb{1}_N\omega - \mathbf{m})\right]}. \tag{S28}$$

Using a Gaussian integral we can write the determinant in Eq. (S28) in the form

$$\det\left[(\mathbb{1}_N\omega^\star - \mathbf{m}^T)(\mathbb{1}_N\omega - \mathbf{m})\right]^{-1} = \int \prod_{i,\alpha}\left(\frac{d^2 z_i^\alpha}{\pi}\right) \times \exp\left[-\epsilon\sum_i |z_i^\alpha|^2 - \sum_{i,j,k,\alpha,\beta,\gamma} z_i^{\alpha\star}(\omega^\star\delta_{ik}\delta_{\alpha\gamma} - (m^T)_{ik}^{\alpha\gamma})(\omega\delta_{kj}\delta_{\gamma\beta} - m_{kj}^{\gamma\beta})z_j^\beta\right], \tag{S29}$$

where ϵ is a positive infinitesimal quantity introduced so as to avoid singularities which occur when ω is equal to one of the eigenvalues of $\mathbf{m}$. To carry out the average over the ensemble of random matrices we need to evaluate

$$\int \prod_{ij\alpha\beta}\left(dm_{ij}^{\alpha\beta}\right)\sqrt{\frac{N}{2\pi\sigma^2(1-\Gamma_{\alpha\beta}^2)}}\exp\left[-\frac{N}{\sigma^2(1-\Gamma_{\alpha\beta}^2)}\left[(m_{ij}^{\alpha\beta})^2 - \Gamma_{\alpha\beta}m_{ij}^{\alpha\beta}m_{ji}^{\beta\alpha}\right]\right] \times \det\left[(\mathbb{1}_N\omega^\star - \mathbf{m}^T)(\mathbb{1}_N\omega - \mathbf{m})\right]^{-1}. \tag{S30}$$

We next perform a Hubbard–Stratonovich transformation [S31] in order to linearise the terms involving $m_{ij}^{\alpha\beta}$. This yields

$$\det\left[(\mathbb{1}_N\omega^\star - \mathbf{m}^T)(\mathbb{1}_N\omega - \mathbf{m})\right]^{-1} = \int \prod_{i\alpha}\left(\frac{d^2 z_i^\alpha d^2 y_i^\alpha}{2\pi^2}\right)\exp\left[-\sum_{i\alpha} y_i^{\alpha\star}y_i^\alpha + \epsilon z_i^{\alpha\star}z_i^\alpha\right] \times \exp\left[i\sum_{ij\alpha\beta} z_i^{\alpha\star}\left(m_{ji}^{\beta\alpha} - \omega^\star\delta_{ij}\delta_{\alpha\beta}\right)y_j^\beta\right]\exp\left[i\sum_{ij\alpha\beta} y_i^{\alpha\star}\left(m_{ij}^{\alpha\beta} - \omega\delta_{ij}\delta_{\alpha\beta}\right)z_j^\beta\right]. \tag{S31}$$

Average over the random matrix and introduction of order parameters

Carrying out the average over the random matrix elements in Eq. (S31) then gives [recalling the definition of the potential $\Phi(\omega)$ in Eq. (S23)]

$$\exp\left[-N\Phi(\omega)\right] = \int \prod_{i\alpha}\left(\frac{d^2 z_i^\alpha d^2 y_i^\alpha}{2\pi^2}\right) \exp\left[-\sum_{i\alpha} y_i^{\alpha\star} y_i^\alpha + \epsilon z_i^{\alpha\star} z_i^\alpha\right]$$
$$\times \exp\left[-i\sum_{i\alpha} z_i^{\alpha\star} y_i^\alpha (\omega^\star + d_\alpha) + z_i^\alpha y_i^{\alpha\star}(\omega + d_\alpha)\right]$$
$$\times \exp\left[-\frac{\sigma^2}{2N}\sum_{ij\alpha\beta}(z_i^{\alpha\star} y_j^\beta + z_i^\alpha y_j^{\beta\star})^2 + \Gamma_{\alpha\beta}(z_i^{\alpha\star} y_j^\beta + z_i^\alpha y_j^{\beta\star})(z_j^{\beta\star} y_i^\alpha + z_j^\beta y_i^{\alpha\star})\right]. \quad \text{(S32)}$$

Following [S26, S29] we disregard contributions containing $N^{-1}\sum_i (y_i^{\alpha\star})^2$, $N^{-1}\sum_i (z_i^{\alpha\star})^2$, $N^{-1}\sum_i (y_i^\alpha)^2$, $N^{-1}\sum_i (z_i^\alpha)^2$, $N^{-1}\sum_i z_i^{\alpha\star} y_i^{\alpha\star}$ and $N^{-1}\sum_i z_i^\alpha y_i^\alpha$. These do not contribute to leading order in N.

We now introduce the order parameters

$$u = \frac{1}{N}\sum_{i\alpha} z_i^{\alpha\star} z_i^\alpha, \quad v = \frac{1}{N}\sum_{i\alpha} y_i^{\alpha\star} y_i^\alpha,$$
$$w_\alpha = \frac{1}{N_\alpha}\sum_i z_i^{\alpha\star} y_i^\alpha, \quad w_\alpha^\star = \frac{1}{N_\alpha}\sum_i y_i^{\alpha\star} z_i^\alpha, \quad \text{(S33)}$$

which we impose in the integral Eq. (S32) using Dirac delta functions in their complex exponential representation. For example

$$\delta(u - \frac{1}{N}\sum_{i\alpha} z_i^{\alpha\star} z_i^\alpha) \propto \int d\hat{u} \exp\left[i\hat{u}(Nu - \sum_{i\alpha} z_i^{\alpha\star} z_i^\alpha)\right],$$
$$\delta(w_\alpha - \frac{1}{N_\alpha}\sum_i z_i^{\alpha\star} y_i^\alpha) \propto \int d^2\hat{w}_\alpha \exp\left[i\hat{w}_\alpha^\star(N_\alpha w_\alpha - \sum_i z_i^{\alpha\star} y_i^\alpha) + i\hat{w}_\alpha(N_\alpha w_\alpha^\star - \sum_i z_i^\alpha y_i^{\alpha\star})\right], \quad \text{(S34)}$$

where we note that w_α is a complex quantity. We can thus rewrite Eq. (S32) as

$$\exp\left[-N\Phi(\omega)\right] = \int \mathcal{D}[\cdots] \exp\left[N(\Psi + \Theta + \Omega)\right], \quad \text{(S35)}$$

where where $\mathcal{D}[\cdots]$ denotes integration over all of the order parameters and their conjugate ('hatted') variables, and where

$$\Psi = i\hat{u}u + i\hat{v}v + i\sum_\alpha \gamma_\alpha(\hat{w}_\alpha w_\alpha^\star + \hat{w}_\alpha^\star w_\alpha),$$
$$\Theta = -\epsilon u - v - \sigma^2 uv + \sum_\alpha \gamma_\alpha\left[-iw_\alpha(\omega^\star + d_\alpha) - iw_\alpha^\star(\omega + d_\alpha)\right] - \frac{1}{2}\sigma^2 \sum_{\alpha\beta}\gamma_\alpha\gamma_\beta\Gamma_{\alpha\beta}(w_\alpha w_\beta + w_\alpha^\star w_\beta^\star),$$
$$\Omega = \sum_\alpha \gamma_\alpha \ln\left[\int \left(\frac{d^2 z^\alpha d^2 y^\alpha}{2\pi^2}\right)\exp\left\{-i(\hat{u}z^{\alpha\star} z^\alpha + \hat{v}y^{\alpha\star} y^\alpha + \hat{w}_\alpha y^{\alpha\star} z^\alpha + \hat{w}_\alpha^\star z^{\alpha\star} y^\alpha)\right\}\right]. \quad \text{(S36)}$$

We recall that α can take the values $\alpha = u$ (prey) and $\alpha = v$ (predators), and that γ_u and γ_v are the fraction of prey and predator species respectively.

We note that the integrals over y_i^α and z_i^α are uncoupled for different values of i as a result of introducing the order parameters in Eq. (S33). Carrying out the integrals over the variables y^α and z^α in the expression for Ω one obtains

$$\Omega = -\sum_\alpha \gamma_\alpha \ln(\hat{w}_\alpha \hat{w}_\alpha^\star - \hat{u}\hat{v}). \tag{S37}$$

Saddle-point integration and evalulation of order parameters

We now take the limit $N \to \infty$, and carry out the integral in Eq. (S35) in the saddle-point approximation. To do this, we extremise the expression $\Psi + \Theta + \Omega$. Extremising with respect to the conjugate variables $\hat{u}, \hat{v}, \hat{w}_\alpha$ and $\hat{w}_\alpha^\star$, we find

$$iu = \sum_\alpha \frac{-\gamma_\alpha \hat{v}}{\hat{w}_\alpha \hat{w}_\alpha^\star - \hat{u}\hat{v}}, \quad iv = \sum_\alpha \frac{-\gamma_\alpha \hat{u}}{\hat{w}_\alpha \hat{w}_\alpha^\star - \hat{u}\hat{v}}, \quad i\gamma_\alpha w_\alpha = \frac{\gamma_\alpha \hat{w}_\alpha}{\hat{w}_\alpha \hat{w}_\alpha^\star - \hat{u}\hat{v}}, \quad i\gamma_\alpha w_\alpha^\star = \frac{\gamma_\alpha \hat{w}_\alpha^\star}{\hat{w}_\alpha \hat{w}_\alpha^\star - \hat{u}\hat{v}}. \tag{S38}$$

One makes the following observation

$$i\hat{u}u + i\hat{v}v + i\sum_\alpha \gamma_\alpha (\hat{w}_\alpha w_\alpha^\star + \hat{w}_\alpha^\star w_\alpha) = 2. \tag{S39}$$

Further, we find that

$$-w_\alpha w_\alpha^\star = \frac{\hat{w}_\alpha \hat{w}_\alpha^\star}{(\hat{w}_\alpha \hat{w}_\alpha^\star - \hat{u}\hat{v})^2}, \tag{S40}$$

which means that we can now express $\hat{w}_\alpha \hat{w}_\alpha^\star$ in terms of $w_\alpha w_\alpha^\star$ and $\hat{u}\hat{v}$. We also find that

$$-uv = \sum_{\alpha\beta} \frac{\gamma_\alpha \gamma_\beta \hat{u}\hat{v}}{(\hat{w}_\alpha \hat{w}_\alpha^\star - \hat{u}\hat{v})(\hat{w}_\beta \hat{w}_\beta^\star - \hat{u}\hat{v})}, \tag{S41}$$

which means that we can in turn express $\hat{u}\hat{v}$ in terms of $w_\alpha w_\alpha^\star$ and uv. Defining $r = uv$, we can write $\hat{u}\hat{v}$ and $\hat{w}_\alpha \hat{w}_\alpha^\star$ in terms of $w_\alpha w_\alpha^\star$ and r. This means that we can replace $\hat{w}_\alpha \hat{w}_\alpha^\star - \hat{u}\hat{v}$ in the expression for Ω.

So, letting $\hat{q}_\alpha = (\hat{w}_\alpha \hat{w}_\alpha^\star - \hat{u}\hat{v})^{-1}$, we obtain an implicit equation for $\hat{q}_\alpha$ in terms of r and $w_\alpha w_\alpha^\star$:

$$-w_\alpha w_\alpha^\star = \hat{q}_\alpha - \hat{q}_\alpha^2 \frac{r}{\left[\sum_\beta \gamma_\beta \hat{q}_\beta\right]^2}. \tag{S42}$$

We now extremise $\Psi + \Theta + \Omega$ in Eq. (S35) with respect to u, v, w_α and $w_\alpha^\star$. We find

$$\begin{aligned} i\hat{u} &= \epsilon + \sigma^2 v, \quad i\hat{v} = 1 + \sigma^2 u, \\ i\hat{w}_\alpha &= i(\omega + d_\alpha) + \sigma^2 \sum_\beta \Gamma_{\alpha\beta}\gamma_\beta w_\beta^\star, \quad i\hat{w}_\alpha^\star = i(\omega^\star + d_\alpha) + \sigma^2 \sum_\beta \Gamma_{\alpha\beta}\gamma_\beta w_\beta. \end{aligned} \tag{S43}$$

Combining these with Eqs. (S38), one sees that

$$v = (\epsilon + \sigma^2 v) \sum_\alpha \gamma_\alpha \hat{q}_\alpha,$$

$$
\begin{aligned}
u &= (1+\sigma^2 u)\sum_\alpha \gamma_\alpha \hat{q}_\alpha, \\
w_\alpha &= -\left[i(\omega+d_\alpha)+\sigma^2\sum_\beta \Gamma_{\alpha\beta}\gamma_\beta w^\star_\beta\right]\hat{q}_\alpha, \\
w^\star_\alpha &= -\left[i(\omega^\star+d_\alpha)+\sigma^2\sum_\beta \Gamma_{\alpha\beta}\gamma_\beta w_\beta\right]\hat{q}_\alpha.
\end{aligned} \tag{S44}
$$

The first equation in this set has two solutions for $\epsilon \to 0^+$.

First solution:
One solution is $v=0$, implying $r=0$, and $\hat{q}_\alpha = -w_\alpha w^\star_\alpha$. The last two of the above equations yield

$$
\begin{aligned}
1 &= i(\omega+d_\alpha)w^\star_\alpha + \sigma^2\sum_\beta \Gamma_{\alpha\beta}\gamma_\beta w^\star_\alpha w^\star_\beta, \\
1 &= i(\omega^\star+d_\alpha)w_\alpha + \sigma^2\sum_\beta \Gamma_{\alpha\beta}\gamma_\beta w_\alpha w_\beta.
\end{aligned} \tag{S45}
$$

From this, one can solve for the resolvent $G(\omega)=\sum_\alpha \gamma_\alpha w^\star_\alpha$, which we see is an analytic function of ω. This means that the eigenvalue density vanishes in regions of the complex plane for which $r=0$ is the only valid solution (see Eq. (S26).

Second solution:
The other solution to the first of Eq. (S44) is

$$
\sum_\alpha \gamma_\alpha \hat{q}_\alpha = \frac{1}{\sigma^2}. \tag{S46}
$$

This can be solved simultaneously with the following equations in order to find r, w_α and $w^\star_\alpha$ as functions of $\omega = x+iy$

$$
\begin{aligned}
-w_\alpha w^\star_\alpha &= \hat{q}_\alpha - \hat{q}_\alpha^2 \frac{r}{\left[\sum_\beta \gamma_\beta \hat{q}_\beta\right]^2}, \\
w_\alpha &= -\left[i(\omega+d_\alpha)+\sigma^2\sum_\beta \Gamma_{\alpha\beta}\gamma_\beta w^\star_\beta\right]\hat{q}_\alpha, \\
w^\star_\alpha &= -\left[i(\omega^\star+d_\alpha)+\sigma^2\sum_\beta \Gamma_{\alpha\beta}\gamma_\beta w_\beta\right]\hat{q}_\alpha.
\end{aligned} \tag{S47}
$$

Significance of the order parameters

It is important at this point to note that the order parameters defined in Eq. (S33) have some broader significance. The quantities w_α and $w^\star_\alpha$, when evaluated at the saddle point, are related to the resolvent:

$$
G(\omega) = \frac{\partial \Phi(\omega,\omega^\star)}{\partial \omega} = i\sum_\alpha \gamma_\alpha w^\star_\alpha,
$$

$$G^\star(\omega) = \frac{\partial \Phi(\omega, \omega^\star)}{\partial \omega^\star} = i \sum_\alpha \gamma_\alpha w_\alpha. \tag{S48}$$

Imagine momentarily that instead of evaluating $-N\Phi(x,y) = \overline{\ln\det\left[(\mathbb{1}_N \omega^\star - \mathbf{m}^T)(\mathbb{1}_N\omega - \mathbf{m})\right]}$, we had considered the 'potential' of the matrix $m_{ij}^{\alpha\beta} - \omega_\alpha \delta_{\alpha\beta}\delta_{ij}$ [i.e. if we had redone the calculation up until this point but had replaced $\omega \mathbb{1}_N$ in the definition of the resolvent Eq. (S16) with a diagonal matrix $(\boldsymbol{\omega})_{ij}^{\alpha\beta} = \omega_\alpha \delta_{\alpha\beta}\delta_{ij}$]. Then, instead of arriving at Eq. (S48), we would have been able to define

$$\begin{aligned} G_\alpha(\{\omega_\alpha, \omega_\alpha^\star\}) &\equiv \frac{1}{\gamma_\alpha}\frac{\partial \Phi(\{\omega_\alpha, \omega_\alpha^\star\})}{\partial \omega_\alpha} = i w_\alpha^\star, \\ G_\alpha^\star(\{\omega_\alpha, \omega_\alpha^\star\}) &\equiv \frac{1}{\gamma_\alpha}\frac{\partial \Phi(\{\omega_\alpha, \omega_\alpha^\star\})}{\partial \omega_\alpha^\star} = i w_\alpha. \end{aligned} \tag{S49}$$

The result of this line of reasoning is that, at the saddle point, we deduce $iw_\alpha = (iw_\alpha^\star)^\star$. Such a deduction is still valid once we set $\omega_\alpha = \omega$. This observation will prove important for ruling out invalid solutions when we solve the saddle-point equations.

Although the fact that at the saddle-point $iw_\alpha = (iw_\alpha^\star)^\star$ may at first seem troubling, one should note the following. Let $w_\alpha = q_\alpha + is_\alpha$ and $w_\alpha^\star = q_\alpha - is_\alpha$. The (real) variables over which we integrate in Eq. (S35), in order to impose the delta function in Eq. (S34), are q_α and s_α. Because the argument of the exponential in Eq. (S35) is complex, we have to use analytical continuation to deform the integrals, which are initially over the real axis, to contours in the complex plane. These contours include the saddle point. As such, the ostensibly 'real' variables q_α and s_α take on complex values at the saddle point. Hence, w_α and $w_\alpha^\star$ are no longer necessarily complex conjugates when evaluated at the saddle point.

Calculation of boundary of bulk spectrum

Inside the support of the eigenvalue spectrum (where the eigenvalue density is non-zero), we have $r > 0$ and the condition Eq. (S46). Assuming no discontinuities in the value of r as a function of ω, the boundary of the support of the eigenvalue spectrum is defined by points ω in the complex plane where r approaches zero, but where Eq. (S46) is still satisfied. This yields the following set of equations which one can solve for the curve defining the boundary $\omega_y(\omega_x)$ (where $\omega = \omega_x + i\omega_y$):

$$\begin{aligned} &\sum_\alpha \gamma_\alpha \hat{q}_\alpha = \frac{1}{\sigma^2}, \\ &-w_\alpha w_\alpha^\star = \hat{q}_\alpha, \\ &\qquad w_\alpha = -\left[i(\omega_x + i\omega_y + d_\alpha) + \sigma^2 \sum_\beta \Gamma_{\alpha\beta}\gamma_\beta w_\beta^\star\right]\hat{q}_\alpha, \\ &\qquad w_\alpha^\star = -\left[i(\omega_x - i\omega_y + d_\alpha) + \sigma^2 \sum_\beta \Gamma_{\alpha\beta}\gamma_\beta w_\beta\right]\hat{q}_\alpha. \end{aligned} \tag{S50}$$

That is, values of ω which lie on the boundary of the support of the eigenvalue spectrum must satisfy the above simultaneous equations.

Since $iw_\alpha = (iw_\alpha^\star)^\star$ at the saddle point, the second relation in Eq. (S50) dictates that $\hat{q}_\alpha$ is real and positive. In solving Eqs. (S50), we therefore discard solutions which do not satisfy $\hat{q}_\alpha \in \mathbb{R}$ and $\hat{q}_\alpha > 0$. Alternatively, letting $iw_\alpha^\star = (iw_\alpha)^\star = \chi_\alpha$, we obtain

$$\sum_\alpha \gamma_\alpha \left|\chi_\alpha\right|^2 = \frac{1}{\sigma^2},$$
$$-1 = -(\omega_x + i\omega_y + d_\alpha)\chi_\alpha + \sigma^2 \sum_\beta \Gamma_{\alpha\beta}\gamma_\beta\chi_\alpha\chi_\beta. \tag{S51}$$

S3. OUTLIER EIGENVALUES

A. Setup and general relations

We now wish to find the outliers that arise when we perturb the random matrix $\mathbf{m}$ by introducing a finite mean. That is, we are now interested in the matrix $\mathbf{m}' \equiv \mathbf{m} + \frac{1}{N}\boldsymbol{\mu}$, where the $N\times$N matrix $\boldsymbol{\mu}$ accounts for the non-zero mean. Specifically $\mathbf{m}'$ has elements

$$m_{ij}'^{\alpha\beta} = m_{ij}^{\alpha\beta} + \frac{\mu_{\alpha\beta}}{N}, \tag{S52}$$

in particular

$$(\boldsymbol{\mu})_{ij}^{\alpha\beta} = \mu_{\alpha\beta}, \tag{S53}$$

independently of i and j. The elements of $\mathbf{m}$ have zero mean as before. So, the elements within each of the 4 blocks $[(\alpha,\beta) = (u,u),(u,v),(v,u),(v,v)]$ of the matrix $\mathbf{m}'$ have the same mean, $\mu_{\alpha\beta}/N$. Following the conventions of statistical physics, the mean is chosen to be proportional to $1/N$ for technical convenience. In the main paper, the mean is independent of N. We explain the mapping between the two setups in Section S4, where we also discuss general values of connectance $0 < C < 1$.

The problem amounts to finding the eigenvalues of the matrix $\mathbf{m}'$ – i.e. $\mathbf{m}$ with a rank-2 perturbation $\boldsymbol{\mu}$. We follow a procedure similar to Ref. [S27]. The bulk of the eigenvalue spectrum is unchanged by the low-rank perturbation. Therefore we look for additional eigenvalues that lie outside of the bulk of eigenvalues of $\mathbf{m}$. Such an eigenvalue λ must satisfy

$$\det\left[\lambda \mathbb{1}_N - \mathbf{m} - \frac{1}{N}\boldsymbol{\mu}\right] = 0. \tag{S54}$$

Any outlier λ, is by definition, not an eigenvalue of the unperturbed matrix $\mathbf{m}$. Therefore the matrix $\mathbb{1}_N\lambda - \mathbf{m}$ is invertible. Hence,

$$\det\left[\mathbb{1}_N - \frac{1}{N}(\lambda\mathbb{1}_N - \mathbf{m})^{-1}\boldsymbol{\mu}\right] = 0. \tag{S55}$$

B. Simplification to an effective 2-species problem

Now we define the resolvent matrix $\mathbf{G} = (\lambda\mathbb{1}_N - \mathbf{m})^{-1}$.

Case $N_u = N_v$:

We suppose for now that there are the same numbers of prey species as predator species, i.e. $N_u = N_v$. We will relax this assumption once we have outlined the proof for this simpler case. Suppose we rearrange the rows and columns of the matrices so that the first species is a predator species, the second a prey species, the third again a predator species, and so on. The matrix $\boldsymbol{\mu}$ then consists of $N_u \times N_u$ block of size 2×2 of the form $\boldsymbol{\mu}^{(2)} \equiv \begin{pmatrix} \mu_{uu} & \mu_{uv} \\ \mu_{vu} & \mu_{vv} \end{pmatrix}$. Then, the matrix $\boldsymbol{\mu}$ can be written in the form

$$\boldsymbol{\mu} = \underline{u}\,\underline{v}^T, \tag{S56}$$

where $\underline{u}$ and $\underline{v}$ are vectors of length N_u. Each entry of $\underline{v}$ is given by the 2×2 matrix $\boldsymbol{\mu}^{(2)}$,

$$\underline{v}^T = \left(\boldsymbol{\mu}^{(2)}, \boldsymbol{\mu}^{(2)}, \cdots\right), \tag{S57}$$

The vector $\underline{u}$ is of the form

$$\underline{u} = (\mathbb{1}_2, \mathbb{1}_2, \mathbb{1}_2, \cdots)^T. \tag{S58}$$

We next use Sylvester's determinant identity

$$\det\left[\mathbb{1}_m + \mathbf{A}\,\mathbf{B}\right] = \det\left[\mathbb{1}_k + \mathbf{B}\,\mathbf{A}\right], \tag{S59}$$

valid for combinations of $m \times k$ matrices $\mathbf{A}$ and $k \times m$ matrices $\mathbf{B}$. We note that the matrices on the left-hand side of Eq. (S59) are of size $m \times m$, and those on the right-hand-side are of size $k \times k$.

Using this identity, we find that $\det(\mathbb{1}_2 - \frac{1}{N}\underline{v}^T(\mathbb{1}_N\lambda - \mathbf{m})^{-1}\underline{u}) = 0$. Thus we obtain

$$\det\left[\mathbb{1}_2 - \boldsymbol{\mu}^{(2)}\frac{1}{N}\sum_{ij}\mathbf{G}^{(ij)}(\lambda)\right] = 0, \tag{S60}$$

where $\left(\mathbf{G}^{(ij)}\right)_{\alpha\beta} = \left((\mathbb{1}_N\lambda - \mathbf{m})^{-1}\right)_{ij}^{\alpha\beta}$.

General case (N_u not necessarily equal to N_v):

We now wish to show that Eq. (S60) also applies in the case $N_u \neq N_v$. This is done using the following identity for block matrices: If A, B, C and D are matrices of sizes $m \times m$, $m \times k$, $k \times m$ and $k \times k$ respectively, then we have

$$\det\begin{bmatrix} A & B \\ 0 & D \end{bmatrix} = \det(A)\det(D) = \begin{bmatrix} A & 0 \\ C & D \end{bmatrix},$$
$$\begin{bmatrix} A & B \\ C & D \end{bmatrix}\begin{bmatrix} A' & B' \\ C' & D' \end{bmatrix} = \begin{bmatrix} AA' + BC' & AB' + BD' \\ CA' + DC' & CB' + DD' \end{bmatrix}. \tag{S61}$$

First we note that the determinant $\det\left[\mathbb{1}_N - \frac{1}{N}\mathbf{G}\boldsymbol{\mu}\right]$ in Eq. (S55) is unchanged by adding extra rows and columns to the matrices in the following way: We add rows and columns $\boldsymbol{\mu}$ to produce a

square matrix $\boldsymbol{\mu}_{N^\star}$ of dimension $N^\star = N + |N_u - N_v|$ which can be decomposed as in Eq. (S56). We then augment $\mathbf{G}$ to obtain

$$\mathbf{G}_{N^\star} = \begin{bmatrix} \mathbf{G} & 0 \\ 0 & 0 \end{bmatrix}. \tag{S62}$$

Then, using the theorems in Eq. (S61), one can show

$$\det\left[\mathbb{1}_{N^\star} - \frac{1}{N}\mathbf{G}_{N^\star}\boldsymbol{\mu}_{N^\star}\right] = \det\left[\mathbb{1}_N - \frac{1}{N}\mathbf{G}\boldsymbol{\mu}\right]. \tag{S63}$$

Hence, we can apply Sylvester's determinant identity as before and arrive at Eq. (S60).

C. Proof that the resolvent matrix is diagonal

We have shown that Eq. (S60) holds when the numbers of predator and prey species are unequal. It remains now to find the averaged Green's functions $\frac{1}{N}\sum_{ij}\mathbf{G}^{(ij)}(\lambda)$. For this, we follow [S15] and use the so-called 'locator expansion' to show that the resolvent matrix is diagonal in the limit $N \to \infty$, i.e. that $G_{ij}^{\alpha\beta} \propto \delta_{ij}\delta_{\alpha\beta}$. Letting $(G^{-1})_{ij}^{\alpha\beta} = (f^{-1})_i^\alpha \delta_{\alpha\beta}\delta_{ij} - m_{ij}^{\alpha\beta}$ (where in our case $(f^{-1})_i^\alpha = \lambda$), it follows that $\mathbf{m} = \mathbf{G}^{-1}(\mathbf{G} - \mathbf{f})\mathbf{f}^{-1}$, from which we obtain a Dyson equation $\mathbf{G} = \mathbf{f} + \mathbf{Gmf}$. This allows us to construct a series for the resolvent

$$G_{ij}^{\alpha\beta} = f_i^\alpha + f_i^\alpha m_{ij}^{\alpha\beta} f_j^\beta + f_i^\alpha m_{ik}^{\alpha\gamma} f_k^\gamma m_{kj}^{\gamma\beta} f_j^\beta + f_i^\alpha m_{ik}^{\alpha\gamma} f_k^\gamma m_{kl}^{\gamma\delta} f_l^\delta m_{lj}^{\delta\beta} f_j^\beta + \dots\,, \tag{S64}$$

where we have omitted sums over repeated indices for brevity. We find that we have to evaluate terms such as $\sum_{k,\gamma} m_{ik}^{\alpha\gamma} f_k^\gamma m_{kj}^{\gamma\beta} \approx \sum_{k\gamma} f_k^\gamma \overline{m_{ik}^{\alpha\gamma} m_{kj}^{\gamma\beta}}$. This involves averages over products of Gaussian variables. Such terms can be simplified using standard Gaussian combinatorics

$$\overline{m_{i_1 i_1'}^{\alpha_1\beta_1} m_{i_2 i_2'}^{\alpha_2\beta_2} \cdots m_{i_n i_n'}^{\alpha_n\beta_n}} = \sum\prod \overline{m_{i_a i_a'}^{\alpha_a\beta_a} m_{i_b i_b'}^{\alpha_b\beta_b}}, \tag{S65}$$

which is valid for even n and is zero otherwise, and where $\sum\prod$ stands for the sum of the products of all possible combinations of pairs $\overline{m_{i_a i_a'}^{\alpha_a\beta_a} m_{i_b i_b'}^{\alpha_b\beta_b}}$. Since we have

$$\overline{m_{i_a i_a'}^{\alpha_a\beta_a} m_{i_b i_b'}^{\alpha_b\beta_b}} = \frac{\sigma^2}{N}\delta_{i_a i_b}\delta_{i_a' i_b'}\delta_{\alpha_a\alpha_b}\delta_{\beta_a\beta_b} + \frac{\sigma^2\Gamma_{\alpha_a\beta_a}}{N}\delta_{i_a i_b'}\delta_{i_a' i_b}\delta_{\alpha_a\beta_b}\delta_{\beta_a\alpha_b}, \tag{S66}$$

one can see that (since the only free indices are i, j, α and β in Eq. (S64)) that $G_{ij}^{\alpha\beta} \propto \delta_{ij}\delta_{\alpha\beta}$. We therefore find that

$$\sum_{ij} G_{ij}^{\alpha\beta}(\lambda) \approx \delta_{\alpha\beta}\sum_i G_{ii}^{\alpha\beta}(\lambda), \tag{S67}$$

for large N.

D. Final expression for outliers

Given that the resolvent matrix is approximately diagonal for large N, we only need to calculate the sum of diagonal elements $\frac{1}{N}\sum_i G_{ii}^{\alpha\alpha}$ in Eq. (S60). This can be found from the quantities

involved in the bulk calculation as follows. Consider the potential $\Phi(\{\omega_\alpha, \omega_\alpha^\star\})$ associated with the generalised resolvent $G(\{\omega_\alpha\}) = \frac{1}{N}\sum_{i\alpha} \overline{(\boldsymbol{\omega} - \mathbf{m})^{-1}}$, where $(\boldsymbol{\omega})_{ij}^{\alpha\beta} = \omega_\alpha \delta_{\alpha\beta}\delta_{ij}$. We know that at the saddle point

$$\chi_\alpha \equiv i w_\alpha^\star = \frac{1}{\gamma_\alpha}\frac{\partial\Phi(\{\omega_\alpha, \omega_\alpha^\star\})}{\partial\omega_\alpha}. \tag{S68}$$

The potential is defined by $\Phi(\{\omega_\alpha, \omega_\alpha^\star\}) = -\frac{1}{N}\overline{\ln\det\left[(\boldsymbol{\omega} - \mathbf{m})^\dagger(\boldsymbol{\omega} - \mathbf{m})\right]}$. Now, differentiating with respect to ω_α and using Jacobi's formula for the derivative of a determinant, we have

$$\frac{\partial\Phi(\{\omega_\alpha, \omega_\alpha^\star\})}{\partial\omega_\alpha} = \frac{1}{N}\mathrm{Tr}\left[(\boldsymbol{\omega} - \mathbf{m})^{-1}\frac{\partial(\boldsymbol{\omega} - \mathbf{m})}{\partial\omega_\alpha}\right] = \frac{1}{N}\sum_i G_{ii}^{\alpha\alpha}(\{\omega_\alpha, \omega_\alpha^\star\}). \tag{S69}$$

Now setting $\omega_\alpha = \omega$, we see from Eq. (S60) that the outliers λ obey

$$\det\left[\mathbb{1}_2 - \boldsymbol{\mu}^{(2)}\boldsymbol{\gamma}\boldsymbol{\chi}(\lambda)\right] = 0,$$
$$\boldsymbol{\gamma}(\lambda) = \begin{bmatrix}\gamma_u & 0\\ 0 & \gamma_v\end{bmatrix}, \quad \boldsymbol{\chi}(\lambda) = \begin{bmatrix}\chi_u & 0\\ 0 & \chi_v\end{bmatrix}. \tag{S70}$$

This can be written as

$$\left[\gamma_u\mu_{uu} - \frac{1}{\chi_u(\lambda)}\right]\left[\gamma_v\mu_{vv} - \frac{1}{\chi_v(\lambda)}\right] - \gamma_u\gamma_v\mu_{uv}\mu_{vu} = 0. \tag{S71}$$

The quantities $\chi_\alpha(\lambda)$ satisfy [c.f. Eq. (S51)]

$$\begin{aligned}-1 &= -(\lambda + d_u)\chi_u + \sigma^2\Gamma_u\gamma_u\chi_u^2 + \sigma^2\Gamma_{uv}\gamma_v\chi_v\chi_u,\\ -1 &= -(\lambda + d_v)\chi_v + \sigma^2\Gamma_{uv}\gamma_u\chi_u\chi_v + \sigma^2\Gamma_v\gamma_v\chi_v^2.\end{aligned} \tag{S72}$$

Note that not all solutions of this set of equations are valid. At the saddle point, the order parameter $u = \frac{1}{N}\sum_{i\alpha} z_i^{\alpha\star} z_i^\alpha$ must satisfy $u \geq 0$, and we have $u = (1 + \sigma^2 u)\sum_\alpha \gamma_\alpha|\chi_\alpha|^2$. This means that we must have

$$\sum_\alpha \gamma_\alpha|\chi_\alpha(\lambda)|^2 < \frac{1}{\sigma^2} \tag{S73}$$

in order for the set $(\lambda, \{\chi_\alpha(\lambda)\})$ to be a valid solution to the combination of Equations (S71) and (S72).

Eqs. (S71) and (S72) can be solved numerically for λ, subject to the constraint in (S73). Note that when the left-hand side is equal to the right-hand side in the inequality (S73) the point λ in question is inside the bulk region.

S4. SCALING WITH N AND CONNECTANCE C

There are two key differences between the random matrices used in Sections S2 and S3 [c.f. Eq. (S27)] and those in the main text [see Methods section]. These are: (a) The model parameters are rescaled by factors of N; (b) Not all species interact with all others in the main text (i.e. the connectance can take values smaller than one, $0 < C < 1$). We now describe how to relate the results of the previous sections to the setup in the main text.

A. Rescaling with N

In this Supplement, we used the statistics given in Eqs. (S27) and (S52) for the entries of the random matrix $\mathbf{m}$. These are

$$\begin{aligned} \overline{m_{ij}^{\alpha\beta}} &= \frac{\mu_{\alpha\beta}}{N}, \\ \overline{(m_{ij}^{\alpha\beta})^2} - (\mu_{\alpha\beta})^2 &= \frac{\sigma^2}{N}, \\ \overline{(m_{ij}^{\alpha\beta} - \mu_{\alpha\beta})(m_{ji}^{\alpha'\beta'} - \mu_{\alpha'\beta'})} &= \frac{\Gamma_{\alpha\beta\alpha'\beta'}\sigma^2}{N}. \end{aligned} \tag{S74}$$

The statistics used in Eq. (4) and (5) of the the main paper (and also given in Eq. (S2) of this Supplement) are different. They can be obtained from the above by making the replacements $\mu_{\alpha\beta} \to N\mu_{\alpha\beta}$ and $\sigma^2 \to N\sigma^2$. The results in the main paper have been obtained from those in Sections S2, S3 and S5 by making this replacement.

The theoretical predictions for stability or instability are formally derived in the limit $N \to \infty$, but in practice they are accurate for finite values of N, provided N is not too small. Sizes of $N \approx 50$ are often sufficient to obtain satisfactory agreement (see Fig. S1 below for an example). The spectra in Fig. 2 of the main paper are obtained numerically from matrices of size 750×750, and can be seen to agree very well with the results of the mathematical theory.

B. Taking into account connectance C

A connectance $C < 1$ between species can be accounted for by making a similar replacement of σ^2 and $\mu_{\alpha\beta}$ to the one described above. We now describe how to see this.

In order to find the potential $\Phi(\omega)$ (which contains all the information about the eigenvalue spectrum and could in principle also be used to find the outliers [S29]) defined in Eq. (S23), one needs to carry out an average over the ensemble of random matrices, see e.g. the step from Eq. (S31) to Eq. (S32). We now wish to take into account that any one pair of species only interacts with probability C. We then obtain [c.f. Eq. (S30)]

$$\begin{aligned} &\int \prod_{ij\alpha\beta} \left(dm_{ij}^{\alpha\beta}\right) P(\{m_{ij}^{\alpha\beta}\}) \det\left[(\mathbb{1}_N\omega^\star - \mathbf{m}^T)(\mathbb{1}_N\omega - \mathbf{m})\right]^{-1} \\ &= \int \prod_{ij\alpha\beta} \left(dm_{ij}^{\alpha\beta}\right) \det\left[(\mathbb{1}_N\omega^\star - \mathbf{m}^T)(\mathbb{1}_N\omega - \mathbf{m})\right]^{-1} \\ &\Bigg\{(1-C)\delta(m_{ij}^{\alpha\beta})\delta(m_{ji}^{\beta\alpha}) \\ &+ C\sqrt{\frac{N}{2\pi\sigma^2(1-\Gamma_{\alpha\beta}^2)}} \exp\left[-\frac{N}{\sigma^2(1-\Gamma_{\alpha\beta}^2)}\left[(m_{ij}^{\alpha\beta} - \mu_{\alpha\beta})^2 - \Gamma_{\alpha\beta}(m_{ij}^{\alpha\beta} - \mu_{\alpha\beta})(m_{ji}^{\beta\alpha} - \mu_{\beta\alpha})\right]\right]\Bigg\}. \end{aligned} \tag{S75}$$

Upon evaluating the integral, one obtains

$$
\begin{aligned}
&\exp\left[-N\Phi(\omega)\right] = \int \prod_{i\alpha}\left(\frac{d^2 z_i^\alpha d^2 y_i^\alpha}{2\pi^2}\right)\exp\left[-\sum_{i\alpha} y_i^{\alpha\star}y_i^\alpha + \epsilon z_i^{\alpha\star}z_i^\alpha\right] \\
&\times \exp\left[-i\sum_{i\alpha} z_i^{\alpha\star}y_i^\alpha(\omega^\star + d_\alpha) + z_i^\alpha y_i^{\alpha\star}(\omega + d_\alpha)\right] \\
&\times \prod_{i,j,\alpha,\beta}\left\{(1-C) + C\exp\left[\frac{i}{N}\mu_{\alpha\beta}\left(z_i^{\beta\star}y_j^\alpha + z_i^\beta y_j^{\alpha\star}\right)\right.\right. \\
&\left.\left. - \frac{\sigma^2}{2N}\left[(z_i^{\alpha\star}y_j^\beta + z_i^\alpha y_j^{\beta\star})^2 + \Gamma_{\alpha\beta}(z_i^{\alpha\star}y_j^\beta + z_i^\alpha y_j^{\beta\star})(z_j^{\beta\star}y_i^\alpha + z_j^\beta y_i^{\alpha\star})\right]\right]\right\}. \qquad \text{(S76)}
\end{aligned}
$$

If we set $C = 1$, we recover the term we would normally obtain with all species connected

$$
\begin{aligned}
&\exp\left[-N\Phi(\omega)\right] = \int \prod_{i\alpha}\left(\frac{d^2 z_i^\alpha d^2 y_i^\alpha}{2\pi^2}\right)\exp\left[-\sum_{i\alpha} y_i^{\alpha\star}y_i^\alpha + \epsilon z_i^{\alpha\star}z_i^\alpha\right] \\
&\times \exp\left[-i\sum_{i\alpha} z_i^{\alpha\star}y_i^\alpha(\omega^\star + d_\alpha) + z_i^\alpha y_i^{\alpha\star}(\omega + d_\alpha)\right] \\
&\times \exp\left[\frac{i}{N}\sum_{i,j,\alpha,\beta}\mu_{\alpha\beta}\left(z_i^{\beta\star}y_j^\alpha + z_i^\beta y_j^{\alpha\star}\right)\right. \\
&\left. - \frac{\sigma^2}{2N}\sum_{i,j,\alpha,\beta}(z_i^{\alpha\star}y_j^\beta + z_i^\alpha y_j^{\beta\star})^2 + \Gamma_{\alpha\beta}(z_i^{\alpha\star}y_j^\beta + z_i^\alpha y_j^{\beta\star})(z_j^{\beta\star}y_i^\alpha + z_j^\beta y_i^{\alpha\star})\right]. \qquad \text{(S77)}
\end{aligned}
$$

For individual sets of i, j, α and β, and assuming $N \gg 1$, the expression in the curly brackets in Eq. (S76) can be expanded in orders of $1/N$ to obtain

$$
\begin{aligned}
&\left\{(1-C) + C\exp\left[\frac{i}{N}\mu_{\alpha\beta}\left(z_i^{\beta\star}y_j^\alpha + z_i^\beta y_j^{\alpha\star}\right)\right.\right. \\
&\left.\left. - \frac{\sigma^2}{2N}(z_i^{\alpha\star}y_j^\beta + z_i^\alpha y_j^{\beta\star})^2 + \Gamma_{\alpha\beta}(z_i^{\alpha\star}y_j^\beta + z_i^\alpha y_j^{\beta\star})(z_j^{\beta\star}y_i^\alpha + z_j^\beta y_i^{\alpha\star})\right]\right\} \\
&\approx 1 + \frac{i}{N}C\mu_{\alpha\beta}\left(z_i^{\beta\star}y_j^\alpha + z_i^\beta y_j^{\alpha\star}\right) \\
&- \frac{C\sigma^2}{2N}(z_i^{\alpha\star}y_j^\beta + z_i^\alpha y_j^{\beta\star})^2 + \Gamma_{\alpha\beta}(z_i^{\alpha\star}y_j^\beta + z_i^\alpha y_j^{\beta\star})(z_j^{\beta\star}y_i^\alpha + z_j^\beta y_i^{\alpha\star}) \\
&\approx \exp\left[\frac{i}{N}C\mu_{\alpha\beta}\left(z_i^{\beta\star}y_j^\alpha + z_i^\beta y_j^{\alpha\star}\right)\right. \\
&\left. - \frac{C\sigma^2}{2N}\left[(z_i^{\alpha\star}y_j^\beta + z_i^\alpha y_j^{\beta\star})^2 + \Gamma_{\alpha\beta}(z_i^{\alpha\star}y_j^\beta + z_i^\alpha y_j^{\beta\star})(z_j^{\beta\star}y_i^\alpha + z_j^\beta y_i^{\alpha\star})\right]\right]. \qquad \text{(S78)}
\end{aligned}
$$

Comparing Eq. (S76) [with Eq. (S78) substituted in] to Eq. (S77), we see that the consequence of introducing a connectance $0 < C < 1$ is to scale the system parameters in the following way

$$
\begin{aligned}
\mu_{\alpha\beta} &\to C\mu_{\alpha\beta}, \\
\sigma^2 &\to C\sigma^2. \qquad \text{(S79)}
\end{aligned}
$$

C. Combined effect

In summary, the scaling that one needs to perform in order to obtain the results in the main text from those in Sections S2 and S3 is given by

$$\begin{aligned} \mu_{\alpha\beta} &\to NC\mu_{\alpha\beta}, \\ \sigma^2 &\to NC\sigma^2. \end{aligned} \tag{S80}$$

All simulation results in the main text were performed using the model as defined in Section S1 (which is the model defined in the main text). We note that Eqs. (S51) and (S71-S73) become respectively

$$\begin{aligned} \sum_\alpha \gamma_\alpha \left|\chi_\alpha\right|^2 - \frac{1}{c} &= 0, \\ -(\omega_x + i\omega_y + d_\alpha + q^2 D_\alpha)\chi_\alpha + c\sum_\beta \Gamma_{\alpha\beta}\gamma_\beta\chi_\alpha\chi_\beta + 1 &= 0, \end{aligned} \tag{S81}$$

and

$$\begin{aligned} \left[\gamma_u NC\mu_{uu} - \frac{1}{\chi_u(\omega)}\right]\left[\gamma_v NC\mu_{vv} - \frac{1}{\chi_v(\omega)}\right] - (NC)^2\gamma_u\gamma_v\mu_{uv}\mu_{vu} &= 0, \\ -(\omega + d_u + q^2 D_u)\chi_u + c\Gamma_u\gamma_u\chi_u^2 + c\Gamma_{uv}\gamma_v\chi_v\chi_u &= -1, \\ -(\omega + d_v + q^2 D_v)\chi_v + c\Gamma_{uv}\gamma_u\chi_u\chi_v + c\Gamma_v\gamma_v\chi_v^2 &= -1, \\ \sum_\alpha \gamma_\alpha |\chi_\alpha(\omega)|^2 &< \frac{1}{c}, \end{aligned} \tag{S82}$$

where $c = NC\sigma^2$.

S5. STABILITY CRITERIA

We class the instabilities in our system into two types:

(1) The bulk of the eigenvalue distribution may cross the imaginary axis. This is the type of instability May identified [S32].

(2) One of the outlier eigenvalues may cross the imaginary axis, with the bulk staying in the negative half-plane. Instabilities of this type are discussed for non-spatial systems for example in [S33].

Below, we provide analytical criteria for each of these instabilities. Crucially, we note that an instability of the second kind can be introduced to an otherwise stable system by the inclusion of diffusion. That is, for a finite band of wavenumbers, the spatial system is unstable where the non-spatial system would be stable.

First, we provide criteria for the stability of the non-spatial system, and then we characterise the spatial instability. In the following the model parameters σ^2 and $\mu_{\alpha\beta}$ are scaled in the same way as in the main text, and we allow for general connectance $0 < C < 1$.

A. Instabilities of the non-spatial system

Instability due to bulk eigenvalues, and relation to May's bound

Mathematical condition for instability:
The transition occurs when the boundary of the bulk eigenvalue spectrum first hits the imaginary axis. We generally observe that the bulk of the eigenvalue spectrum is a convex set. Since the matrix we are dealing with is real, we also know that the spectrum is symmetric with respect to the real axis. As a consequence we find that point of first contact is at $\omega = 0$. We note that, from the correspondence with the resolvent shown in the previous section, this means that the variables $\chi_\alpha(0)$ must be real. In order for the system to be stable, $\lambda = 0$ must necessarily be outside the bulk (otherwise there are positive real eigenvalues). Hence, a necessary condition for stability is

$$\sum_\alpha \gamma_\alpha \chi_\alpha^2 < \frac{1}{c}, \tag{S83}$$

We have used Eq. (S73) with the rescaling $\mu_{\alpha\beta} \to C\mu_{\alpha\beta}$, and $\sigma^2 \to C\sigma^2$, and we recall that the complexity parameter is given by $c = NC\sigma^2$. The quantities χ_α satisfy

$$-1 = -d_\alpha \chi_\alpha + c \sum_\beta \Gamma_{\alpha\beta}\gamma_\beta \chi_\alpha \chi_\beta. \tag{S84}$$

The equations above cannot be solved analytically in general, but a numerical solution can be obtained.

Special cases:
We can make the connection with May's original criterion [S32] in several special cases. First, consider the case where $\gamma_v = 0$ – that is, we do not distinguish between predator and prey species, and consider a general community of $N = N_u$ species (as May did). In this case, Eqs. (S83) and (S84) yield

$$\sqrt{c}(1 + \Gamma_{uu}) < d_u. \tag{S85}$$

We recover May's criterion for $\Gamma_{uu} = 0$ and $d_u = 1$. Secondly, the criterion for stability also simplifies greatly when the prey and predator sub-communities have uncorrelated internal interactions so that $\Gamma_u = \Gamma_v = 0$, the same number of species (i.e., $\gamma_u = \gamma_v = \frac{1}{2}$), and equal death rates $d_u = d_v = d$. In this case, Eqs. (S83) and (S84) give

$$\sqrt{2c}(1 + \frac{1}{4}\Gamma_{uv}) < d. \tag{S86}$$

Similarly, if $\Gamma_{uv} = 0$ and $\Gamma_u = \Gamma_v = \Gamma$, we obtain

$$\sqrt{2c}(1 + \frac{1}{4}\Gamma) < d. \tag{S87}$$

A further special case is a situation in which all interactions are uncorrelated ($\Gamma_{\alpha\beta} = 0$ for all α and β). Here we find

$$c \sum_\alpha \frac{\gamma_\alpha}{d_\alpha^2} < 1. \tag{S88}$$

From these examples, we get a general idea of how each of the model parameters affect the onset of instability. The intra-species interaction $I_\alpha = -d_\alpha$ is required to be negative in order to have stability. The more negative I_α is, the more stable the equilibrium is. The equilibrium can also be stabilised by asymmetry in the interaction coefficients. That is, the lower the parameters $-1 \leq \Gamma_{\alpha\beta} \leq 1$ are, the more stable the equilibrium. Finally and crucially, as per May's conclusion, a lower complexity $c = NC\sigma^2$ contributes to making the equilibrium more stable in all the special cases outlined above.

Instability due to outlier eigenvalues

Mathematical condition for instability:
If we suppose that the bulk eigenvalues remain to the left of the imaginary axis, an instability may still occur if one or more of the outlier eigenvalues take on a positive real part. This may occur in two possible ways: (I) A single outlier with no imaginary part may cross at $\lambda = 0$; or (II) A pair of complex conjugate outliers may cross the imaginary axis. The point at which an instability of the first kind occurs can be obtained by substituting $\lambda = 0$ in Eqs. (S71) and (S72). Re-scaling σ^2 and $\mu_{\alpha\beta}$ to match the conventions in the main paper, we obtain

$$\begin{aligned} \left[NC\gamma_u\mu_{uu} - \frac{1}{\chi_u}\right]\left[NC\gamma_v\mu_{vv} - \frac{1}{\chi_v}\right] - (NC)^2\gamma_u\gamma_v\mu_{uv}\mu_{vu} = 0, \\ c\Gamma_u\gamma_u\chi_u^2 + c\Gamma_{uv}\gamma_v\chi_v\chi_u - d_u\chi_u + 1 = 0, \\ c\Gamma_{uv}\gamma_u\chi_u\chi_v + c\Gamma_{vv}\gamma_v\chi_v^2 - d_v\chi_v + 1 = 0. \end{aligned} \tag{S89}$$

The model parameters at which an instability of type (II) above occurs are found as follows: We evaluate the outlier eigenvalues by solving Eqs. (S71) and (S72) subject to Eq. (S73), and we carry out the re-scaling $\mu_{\alpha\beta} \to NC\mu_{\alpha\beta}$, $\sigma^2 \to NC\sigma^2$. We do this for varying model parameters until one of the outliers gains a positive real part. This way, we ensure that we spot any complex conjugate pairs crossing the imaginary axis. The sloped lines in Fig. 3 in the main text, for example, were generated in this way.

Example:
Fig. 2 in the main text shows an example in which a single outlier crosses the imaginary axis. In Fig. S1 we show a case in which a complex-conjugate pair of outliers has crossed the axis. The model parameters are the same as in Fig. 2 of the main text, with the exception of the $\mu_{\alpha\beta}$. This shows that altering the means of the matrix elements can turn an instability of type (I) above into one of type (II). We note that the bulk of the eigenvalue spectrum is the same in Fig. 2a and in Fig. S1. This confirms that the bulk spectrum is not affected by changes in the means $\mu_{\alpha\beta}$.

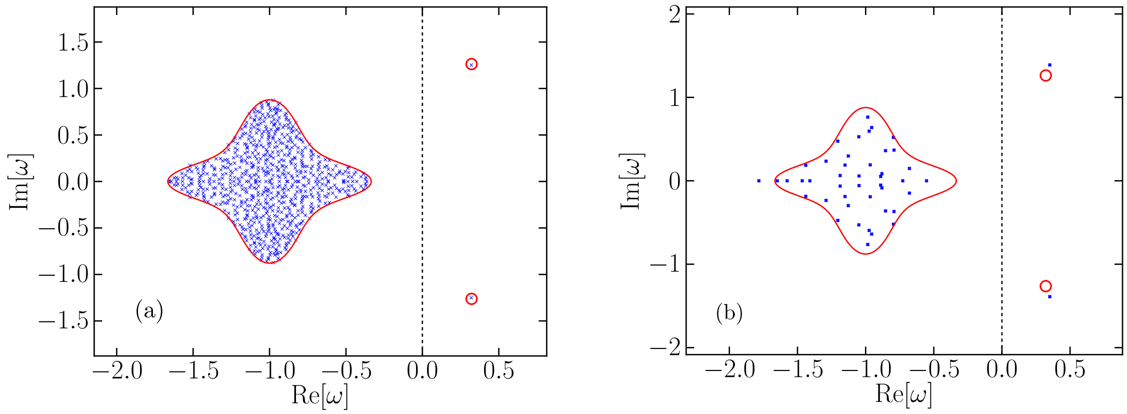

FIG. S1. **Further example of instability due to outlier eigenvalues crossing the imaginary axis.** Eigenvalues obtained numerically from computer-generated random matrices are shown as blue crosses. Red lines are the theoretical predictions for the boundary for the bulk region, and red hollow circles the predictions for the locations of any outliers. The model parameters are the same as in Fig. 2 (a) (with $q = 0$) of the main text, with the exception of the means $\mu_{\alpha\beta}$ which are now given by $CN\mu_{uu} = 4$, $CN\mu_{uv} = -4$, $CN\mu_{vu} = 4$, $CN\mu_{vv} = -2$, where the scaling of the system parameters is the same as in the main text. In the left-hand panel we use $N = 750$, on the right $N = 45$, keeping the products $CN\mu_{\alpha\beta}$ constant.

Special cases:

We can make analytical progress in the same set of special cases as in the previous section. First, consider the case $\gamma_u = 0$ (we could similarly use $\gamma_v = 0$). Then, the outlier eigenvalue [from Eq. (S72)] is real and given by

$$\lambda = NC\mu_{vv} + \frac{\Gamma_v c}{NC\mu_{vv}}. \tag{S90}$$

For $C = 1$ this reduces further to results mentioned in [S27, S29]. A necessary condition for stability is then

$$NC\mu_{vv} + \frac{\Gamma_v c}{NC\mu_{vv}} < 0. \tag{S91}$$

In the case where the prey and predator communities have uncorrelated internal interactions so that $\Gamma_u = \Gamma_v = 0$, the same numbers of species $\gamma_u = \gamma_v = \frac{1}{2}$, and the same death rates $d_u = d_v = d$ and $\mu_{uu} = -\mu_{vv}$, the outlier eigenvalues are given by

$$\lambda = -d \pm \frac{(NC)^2(\mu_{uu}^2 + \mu_{uv}\mu_{vu}) + 2\Gamma_{uv}c}{2NC\sqrt{\mu_{uu}^2 + \mu_{uv}\mu_{vu}}}. \tag{S92}$$

If $\mu_{uu}^2 + \mu_{uv}\mu_{vu} < 0$, there is no real solution for λ and there is guaranteed to be no instability, so long as $d > 0$. We note that complex conjugate pairs of outliers are made possible only by the random matrix having trophic (block) structure.

Similarly, in the case $\Gamma_{uv} = 0$ and $\Gamma_u = \Gamma_v = \Gamma$, the eigenvalues are given by

$$\lambda = -d \pm \frac{(NC)^2(\mu_{uu}^2 + \mu_{uv}\mu_{vu}) + 2\Gamma c}{2NC\sqrt{\mu_{uu}^2 + \mu_{uv}\mu_{vu}}}. \tag{S93}$$

In both special cases, the more negative the quantity $\mu_{uv}\mu_{vu}$ is, the more stable the equilibrium. We note that $-\mu_{uv}\mu_{vu}$ is a measure for the 'strength' of predation (taking into account both the effects on prey and predators). So, predation is a stabilising influence. Further, asymmetry in the interaction coefficients has a stabilising effect.

A further special case is $\Gamma_{\alpha\beta} = 0$ for all α and β. Here, we obtain

$$[NC\gamma_u\mu_{uu} - d_u]\,[NC\gamma_v\mu_{vv} - d_v] - (NC)^2\gamma_u\gamma_v\mu_{uv}\mu_{vu} > 0. \tag{S94}$$

Once again, the more negative the quantity $\mu_{uv}\mu_{vu}$ is, the more stable the equilibrium.

B. Dispersal-induced instability

So far we have considered only a non-spatial ecosystem in this Supplement, i.e. we have set $q = 0$ in Eq. (S4), or equivalently $D_u = D_v = 0$. We now address instabilities induced by dispersal. The additional terms arising from a non-zero dispersal term can be absorbed into a re-definition of d_α,

$$d_\alpha \to d_\alpha + q^2 D_\alpha. \tag{S95}$$

This can be seen directly from Eq. (S6). With the replacement in (S95), we can then use the stability criteria derived in Section S5 to obtain criteria for dispersal-induced instability.

The instability due to bulk eigenvectors is unaffected by dispersal

With the inclusion of dispersal Eqs. (S83) and (S84) become

$$\begin{aligned} \sum_\alpha \gamma_\alpha \chi_\alpha^2 &< \frac{1}{c}, \\ -1 &= -(d_\alpha + q^2 D_\alpha)\chi_\alpha + c\sum_\beta \Gamma_{\alpha\beta}\gamma_\beta\chi_\alpha\chi_\beta. \end{aligned} \tag{S96}$$

This is the condition for the stability of the Fourier mode with wavenumber q.

Although it is difficult to prove analytically, we find in general that the inclusion of dispersal serves only to shift the bulk of the eigenvalues in the negative real direction, never in the positive. This is seen to be the case in Fig. 2 in the main text. This can also be seen to be true in the special cases we have discussed already – increasing the magnitude of d_α (which is effective what one does by introducing non-zero q) in Eqs. (S85)-(S88) serves only to expand the range of parameters for which the equilibrium is stable.

Turing-type instability due to outlier eigenvalues

The Turing instability is a 'stationary' instability [S34] which exhibits no oscillations. This means that the eigenvalue crossing the imaginary axis has no imaginary part (unlike the example given in Fig. S1). The point in parameter space at which an instability of this type occurs in the

non-spatial case ($q = 0$) is given by the solution of Eq. (S89). Using this, and the replacement $d_\alpha \to d_\alpha + q^2 D_\alpha$ to account for $q \neq 0$, we arrive at the condition for stability with respect to disturbances of non-zero wavenumber q:

$$\begin{aligned} P(q^2) \equiv \left[NC\gamma_u\mu_{uu} - \frac{1}{\chi_u}\right]\left[NC\gamma_v\mu_{vv} - \frac{1}{\chi_v}\right] - (NC)^2\gamma_u\gamma_v\mu_{uv}\mu_{vu} > 0, \\ c\Gamma_u\gamma_u\chi_u^2 + c\Gamma_{uv}\gamma_v\chi_v\chi_u - (d_u + D_u q^2)\chi_u + 1 = 0, \\ c\Gamma_{uv}\gamma_u\chi_u\chi_v + c\Gamma_{vv}\gamma_v\chi_v^2 - (d_v + D_v q^2)\chi_v + 1 = 0. \end{aligned} \tag{S97}$$

In order for the ecosystem to show a Turing instability this condition must be satisfied for $q = 0$ (i.e., the system is stable with respect to perturbations with $q = 0$), but violated for some non-zero q (there are unstable modes with $q \neq 0$). That this can indeed be possible is exemplified by the special case where $\Gamma_{\alpha\beta} = 0$ for all α and β. In this case, the stability criterion for the Fourier mode q becomes

$$P_0(q^2) \equiv \left[NC\gamma_u\mu_{uu} - (d_u + D_u q^2)\right]\left[NC\gamma_v\mu_{vv} - (d_v + D_v q^2)\right] - (NC)^2\gamma_u\gamma_v\mu_{uv}\mu_{vu} > 0. \tag{S98}$$

The expression for $P_0(q^2)$ is quadratic in q^2. It has a minimum at

$$q^2_{\min} = \frac{(NC\gamma_u\mu_{uu} - d_u)D_v + (NC\gamma_v\mu_{vv} - d_v)D_u}{2D_u D_v}. \tag{S99}$$

If $P_0(0) > 0$ and $P_0(q^2_{\min}) < 0$, then there is a Turing instability. This occurs when

$$\frac{D_v}{D_u} > \gamma_u\gamma_v\left[\frac{1}{\gamma_u\mu'_{uu}}\left(\sqrt{\mu'_{uu}\mu'_{vv} - \mu_{uv}\mu_{vu}} + \sqrt{-\mu_{uv}\mu_{vu}}\right)\right]^2, \tag{S100}$$

where $NC\gamma_u\mu'_{uu} = NC\gamma_u\mu_{uu} - d_u$ and $NC\gamma_v\mu'_{vv} = NC\gamma_v\mu_{vv} - d_v$. This is very reminiscent of the criterion for Turing instability in more conventional reaction-diffusion systems [S35].

In the more general case, we need to find the minimum possible value of the expression on the left-hand side of the first relation in Eq. (S97). If the minimum value of $P(q^2)$ is negative (i.e. the condition in the first line of Eq. (S97) is violated), but the value of $P(0)$ is positive, then we say that there is a Turing (dispersal-induced) instability for this parameter set.

Again, we first find the minimum value of $P(q^2)$ for a given set of model parameters. To do this, we differentiate $P(q^2)$ with respect to q^2, noting that the three relations in Eq. (S97) are coupled. This leads to the following set of simultaneous equations to find $P(q^2_{\min})$

$$\begin{aligned} P'(q^2_{\min}) &= NC\gamma_u\mu_{uu}\chi'_u\left[NC\gamma_v\chi_v\mu_{vv} - 1\right] + NC\gamma_v\mu_{vv}\chi'_v\left[NC\gamma_u\chi_u\mu_{uu} - 1\right] \\ &\quad - (NC)^2\gamma_u\gamma_v\mu_{uv}\mu_{vu}\left[\chi_u\chi'_v + \chi_v\chi'_u\right] = 0, \\ 0 &= 2c\Gamma_u\gamma_u\chi_u\chi'_u + c\Gamma_{uv}\gamma_v\left(\chi_v\chi'_u + \chi'_v\chi_u\right) - (d_u + D_u q^2_{\min})\chi'_u - D_u\chi_u, \\ 0 &= c\Gamma_{uv}\gamma_u\left(\chi_v\chi'_u + \chi'_v\chi_u\right) + 2c\Gamma_{vv}\gamma_v\chi_v\chi'_v - (d_v + D_v q^2_{\min})\chi'_v - D_v\chi_v, \\ 0 &= c\Gamma_u\gamma_u\chi_u^2 + c\Gamma_{uv}\gamma_v\chi_v\chi_u - (d_u + D_u q^2_{\min})\chi_u + 1, \\ 0 &= c\Gamma_{uv}\gamma_u\chi_u\chi_v + c\Gamma_{vv}\gamma_v\chi_v^2 - (d_v + D_v q^2_{\min})\chi_v + 1, \end{aligned} \tag{S101}$$

where $\chi'_\alpha = \frac{\partial \chi_\alpha}{\partial q^2}$. One solves the above equations simultaneously to find χ_u, χ'_u, χ_v, χ'_v and q^2_{min}. One then evaluates

$$P(q^2_{\text{min}}) = [NC\gamma_u\chi_u\mu_{uu} - 1]\,[NC\gamma_v\chi_v\mu_{vv} - 1] - (NC)^2\gamma_u\gamma_v\mu_{uv}\mu_{vu}\chi_u\chi_v. \tag{S102}$$

So, one can find the minimum value of $P(q^2)$ for a particular parameter set and thus deduce whether or not there is dispersal-induced instability (i.e. whether the condition in Eq. (S97) is satisfied). For the purpose of constructing stability diagrams, one can instead set $P(q^2_{\text{min}}) = 0$, equivalent to assuming that the outlier eigenvalue is just about to cross the imaginary axis ($\lambda = 0$). One then solves for the critical model parameters instead which give $P(q^2_{\text{min}}) = 0$. This can be used to produce solid sloped lines in Figs. 3b and 4 in the main text .

Although the above set of equations appears cumbersome, we note that they are at most linear in χ'_u and χ'_v, so these can be solved for relatively easily. In practice, Eqs. (S101) reduce to three simultaneous equations for q^2_{min}, χ_u and χ_v.

C. Dispersal alone cannot make an unstable equilibrium stable

In the model discussed thus far, there are no instances in which an unstable equilibrium of the non-spatial ecosystem becomes stable when dispersal is introduced. This is because a stable equilibrium in the spatial model must be stable with respect to disturbances for all values of q, including $q = 0$ (which is equivalent to setting $D_\alpha = 0$ and corresponds to the non-spatial case). So, if the non-spatial system is unstable, the $q = 0$ mode in the spatial system must also be unstable.

With the introduction of spatial heterogeneity in the interaction coefficients however, this reasoning no longer applies. This is because spatial heterogeneity in the interaction coefficients introduces a non-trivial interdependence between the eigenvalue spectra for different Fourier modes q. That is, the spectra for different values of q can no longer be calculated independently and, more specifically, the bulk of the $q = 0$ spectrum may 'shrink' as a result of this coupling. Further details can be found in Section S10.

S6. PARAMETERS USED FOR FIGURES IN THE MAIN TEXT

Figure 2:
$N = 750$, $C = 0.5$, $\gamma_u = 2/3$, $\gamma_v = 1/3$, $c = CN\sigma^2 = 0.375$, $d_u = 1$, $d_v = 1$, $\Gamma_u = 0.5$, $\Gamma_{uv} = -0.9$, $\Gamma_v = -0.5$, $CN\mu_{uu} = 3$, $CN\mu_{uv} = -3$, $CN\mu_{vu} = 3$, $CN\mu_{vv} = -1.5$, $D_u = 1$, $D_v = 5$.

Figure 3:
$\Gamma_u = 0.5$, $\Gamma_v = -0.5$, $\Gamma_{uv} = -0.9$, $D_u = 1$, $D_v = 5$, $d_u = d_v = 1$, $CN\mu_{uu} = 3$, $CN\mu_{vv} = -1.5$, $\gamma_u = 2/3$, $\gamma_v = 1/3$, $\mu_{uv} = -\mu_{vu}$.

Figure 4:
$\Gamma_u = 1$, $\Gamma_v = -1$, $\Gamma_{uv} = 0$, $D_u = 1$, $d_u = d_v = 1$, $CN\mu_{uu} = CN\mu_{vu} = 3$, $CN\mu_u = -3$, $CN\mu_{vv} = -1.5$, $\gamma_u = 2/3$, $\gamma_v = 1/3$.

Figure 5:
$N = 100$, $\Gamma_u = 0.5$, $\Gamma_v = -0.5$, $\Gamma_{uv} = 0$, $D_u = 0.2$, $D_v = 20$, $\sqrt{CN}\sigma = 0.1$, $d_u = d_v = 0.1$, $CN\mu_{uu} = 1.2$, $CN\mu_{vu} = 2$, $CN\mu_{uv} = -2$, $CN\mu_{vv} = -0.8$, $a = 0.5$, $\gamma_u = 1/2$, $\gamma_v = 1/2$. In (a), $D_u, D_v = 0$ and in (b) $D_u = 0.2, D_v = 20$

Part II

Robustness against variations of the model

In this part of the Supplement we consider several variations on the model presented previously. In Section S7, we comment on the possibility of universality and show that our main results carry over to non-Gaussian random interaction matrices under relatively mild conditions. Further, while the model in the main text describes trophic structure in a statistical sense, we show in Section S8 that the formalism we develop can also be used when trophic interactions are enforced more strictly. Finally, in Section S9 we address the case in which the diagonal elements of the random interaction matrix are not uniform within blocks. To this end we extend the analytical calculation of the eigenvalue spectra in Part I. We use this to show that heterogeneity in the self-interaction or diffusion coefficients tends to broaden the eigenvalue spectrum, and hence promotes instability. In particular, the possibility of dispersal-induced instability persists in the face of such heterogeneity.

S7. UNIVERSALITY OF THE THEORY – NON-GAUSSIAN RANDOM MATRICES

A. Analytical demonstration of universality

We have so far considered the interaction coefficients (the elements of the matrix $m_{ij}^{\alpha\beta}$) to be drawn from a Gaussian ensemble. We now demonstrate that our results apply to other distributions as well provided some fairly liberal conditions are fulfilled.

Suppose that the random matrix elements $m_{ij}^{\alpha\beta}$, which we take for now to have zero mean, are drawn from a non-Gaussian distribution. We imagine however that the statistics of this non-Gaussian distribution are the same as the previously specified Gaussian distribution up to the second moment and that higher order moments decay sufficiently quickly with N. What is meant by 'sufficiently quickly' will emerge in the calculation that follows.

The first and second moments of the random matrix are

$$\begin{aligned}\overline{m_{ij}^{\alpha\beta}} &= 0,\\ \overline{(m_{ij}^{\alpha\beta})^2} &= \frac{\sigma^2}{N},\\ \overline{m_{ij}^{\alpha\beta} m_{ji}^{\alpha'\beta'}} &= \frac{\Gamma_{\alpha\beta\alpha'\beta'}\sigma^2}{N},\\ m_{ii}^{\alpha\alpha} &= -d_\alpha.\end{aligned} \tag{S103}$$

We begin once again with Eq. (S31). The object of primary interest when performing the average over the ensemble of random interaction matrices is

$$U_{ij}^{\alpha\beta} = \overline{\exp\left[i(z_i^{\alpha\star}y_j^{\beta} + z_i^{\alpha}y_j^{\beta\star})m_{ij}^{\alpha\beta} + i(z_j^{\beta\star}y_i^{\alpha} + z_j^{\beta}y_i^{\alpha\star})m_{ji}^{\beta\alpha}\right]}. \tag{S104}$$

We now expand the exponential as a series to obtain

$$U_{ij}^{\alpha\beta} = \sum_{r=0}^{\infty}\sum_{s=0}^{\infty}\frac{i^{r+s}}{r!s!}(z_i^{\alpha\star}y_j^{\beta} + z_i^{\alpha}y_j^{\beta\star})^r(z_j^{\beta\star}y_i^{\alpha} + z_j^{\beta}y_i^{\alpha\star})^s\overline{(m_{ij}^{\alpha\beta})^r(m_{ji}^{\beta\alpha})^s}. \tag{S105}$$

We now assume that the higher-order moments (such that $r+s>2$) are $\mathcal{O}(N^{-(1+\epsilon)})$ where $\epsilon>0$, as would be the case with the original Gaussian distribution. This means that all moments $r+s>0$ fall off with N faster than $1/N$. Since we take into account only the leading-order contribution in N^{-1}, this allows us to truncate the series in Eq. (S105), carry out the average over the disorder and re-exponentiate as follows

$$\begin{aligned} U_{ij}^{\alpha\beta} &\approx 1-\frac{\sigma^2}{2N}\left[(z_i^{\alpha\star}y_j^{\beta}+z_i^{\alpha}y_j^{\beta\star})^2+(z_j^{\beta\star}y_i^{\alpha}+z_j^{\beta}y_i^{\alpha\star})^2+2\Gamma_{\alpha\beta}(z_i^{\alpha\star}y_j^{\beta}+z_i^{\alpha}y_j^{\beta\star})(z_j^{\beta\star}y_i^{\alpha}+z_j^{\beta}y_i^{\alpha\star})\right] \\ &\approx \exp\left\{-\frac{\sigma^2}{2N}\left[(z_i^{\alpha\star}y_j^{\beta}+z_i^{\alpha}y_j^{\beta\star})^2+(z_j^{\beta\star}y_i^{\alpha}+z_j^{\beta}y_i^{\alpha\star})^2+2\Gamma_{\alpha\beta}(z_i^{\alpha\star}y_j^{\beta}+z_i^{\alpha}y_j^{\beta\star})(z_j^{\beta\star}y_i^{\alpha}+z_j^{\beta}y_i^{\alpha\star})\right]\right\}. \end{aligned} \tag{S106}$$

Once we sum over all combinations of $(\alpha,\beta;i,j)$ in the exponent, we find the same result as in Eq. (S32) and the calculation continues as before. This reasoning implies that the results in Eqs. (S50) are valid for a wide class of matrices, whose elements may be drawn from non-Gaussian distributions. We emphasize that all that is required for Eq. (S32) to be valid is that Eq. (S103) be satisfied and that any higher moments than the variance decrease sufficiently quickly with N. Similar arguments can also be used when the first moments of the random matrix elements are non-zero and so we should expect Eqs. (S71-S73) to be generally applicable in the same fashion.

B. Verification with Bernoulli, Laplace and uniformly distributed random numbers

To demonstrate the universality of our theory, we compare the eigenvalue spectra for random matrices whose elements are drawn from different distributions. In Fig. S2 we show that the locations of the eigenvalues are predicted accurately by our theory.

We first verify the universality of our theoretical approach using Bernoulli random numbers. Here, the joint probability distribution for the random matrix entries is

$$\begin{aligned} P(m_{ij}^{\alpha\beta},m_{ji}^{\beta\alpha}) &= \frac{(1+\Gamma_{\alpha\beta})}{4}\left[\delta(m_{ij}^{\alpha\beta}-\sigma/\sqrt{N})\delta(m_{ji}^{\beta\alpha}-\sigma/\sqrt{N})+\delta(m_{ij}^{\alpha\beta}+\sigma/\sqrt{N})\delta(m_{ji}^{\beta\alpha}+\sigma/\sqrt{N})\right] \\ &+\frac{(1-\Gamma_{\alpha\beta})}{4}\left[\delta(m_{ij}^{\alpha\beta}-\sigma/\sqrt{N})\delta(m_{ji}^{\beta\alpha}+\sigma/\sqrt{N})+\delta(m_{ij}^{\alpha\beta}+\sigma/\sqrt{N})\delta(m_{ji}^{\beta\alpha}-\sigma/\sqrt{N})\right]. \end{aligned} \tag{S107}$$

We note that this is for the case in which the mean vanishes, $\mu_{\alpha\beta}=0$. A non-zero mean can be added subsequently.

We also verify our universality claim with random numbers drawn from a Laplace distribution,

$$\begin{aligned} P(m_{ij}^{\alpha\beta},m_{ji}^{\beta\alpha}) &= \sqrt{\frac{N}{2\sigma^2}}\exp\left(-\frac{\sqrt{2N}\left|m_{ij}^{\alpha\beta}\right|}{\sigma}\right) \\ &\times\left[\frac{(1+\Gamma_{\alpha\beta})}{2}\delta(m_{ij}^{\alpha\beta}-m_{ji}^{\beta\alpha})+\frac{(1-\Gamma_{\alpha\beta})}{2}\delta(m_{ij}^{\alpha\beta}+m_{ji}^{\beta\alpha})\right], \end{aligned} \tag{S108}$$

and a uniform distribution,

$$P(m_{ij}^{\alpha\beta},m_{ji}^{\beta\alpha}) = \frac{\sqrt{N}}{2\sqrt{3}\sigma}\theta\left(m_{ij}^{\alpha\beta}-\frac{\sqrt{3}\sigma}{\sqrt{N}}\right)\theta\left(m_{ij}^{\alpha\beta}+\frac{\sqrt{3}\sigma}{\sqrt{N}}\right)$$

$$\times \left[\frac{(1+\Gamma_{\alpha\beta})}{2} \delta(m_{ij}^{\alpha\beta} - m_{ji}^{\beta\alpha}) + \frac{(1-\Gamma_{\alpha\beta})}{2} \delta(m_{ij}^{\alpha\beta} + m_{ji}^{\beta\alpha}) \right]. \tag{S109}$$

In Section S8, we also use a Gamma distribution. The probability distributions in Eqs. (S107–S109) are constructed such that the elements of the random matrices obey the relations in Eq. (S103).

Examples of spectra of matrices with these statistics are shown in Fig. S2. As can be seen from the data, the limiting spectra are independent of the details of the distribution and are well-described by the analytical theory [given in Eqs. (S51), (S71) and (S72)].

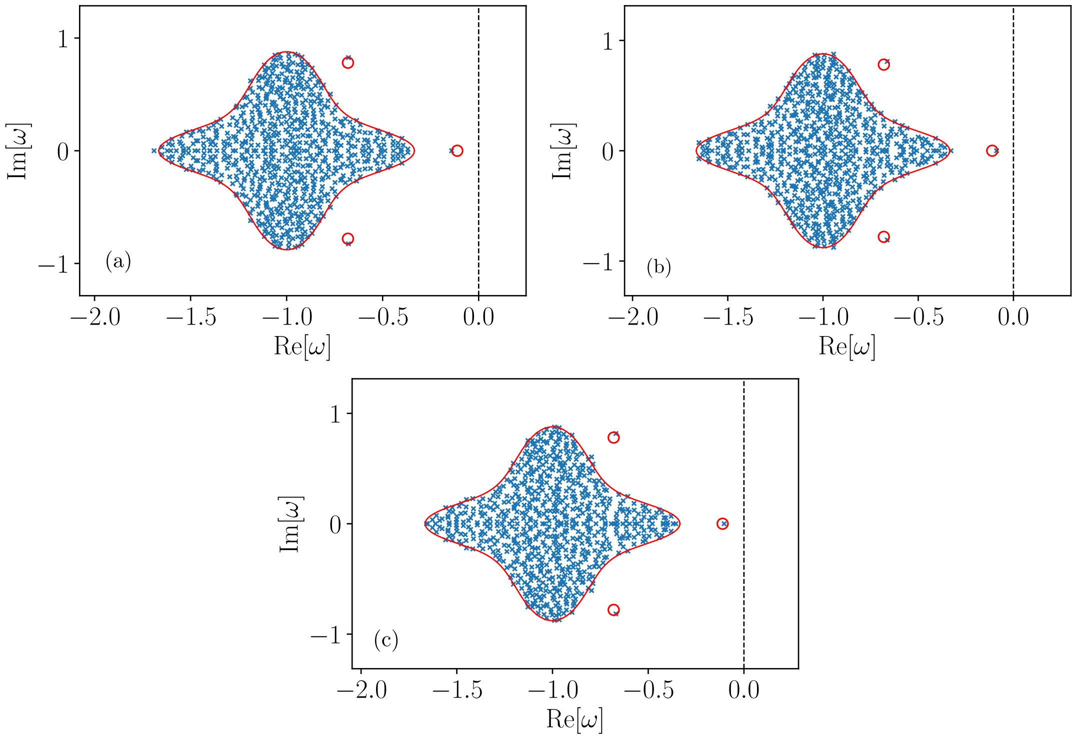

FIG. S2. **Universality: eigenvalue spectra only depend on first two moments of interaction matrix.** The eigenvalues of a random matrix $A_{ij}^{\alpha\beta}$ with non-Gaussian statistics. Panel (a): Bernoulli distribution [Eq. (S107)], panel (b): Laplace distribution [Eq. (S108)], panel (c): uniform distribution [Eq. (S109)]. All distributions fulfil Eq. (S103) but also have non-zero $\overline{m_{ij}^{\alpha\beta}} = \mu_{\alpha\beta}/N$. Model parameters are the same as in Fig. 2a in the main text with $N = 750$. Blue markers are from computer-generated matrices, the red lines and circles are the predictions of the analytical theory.

S8. STRICT ENFORCEMENT OF TROPHIC INTERACTIONS

In the first instance, we employed Gaussian distributions as the prototype for the distribution from which the elements of our random matrices were drawn. This does not necessarily guarantee that all interactions between, say, predator species and prey species will be assigned negative coefficients for the prey-like species and positive coefficients for the predator-like species. Due to the choice of the first moments there is a statistical tendency for this to be the case but it is not strictly enforced for each and every pair due to the unbounded support of the bivariate Gaussian distribution. Similarly, these distributions do not preclude the possibility of predator-prey-like

interactions between two prey species or two predator species (intraguild predation). The blocks in the matrix therefore do not describe strict trophic levels. Instead, the block structure divides the community into species which are more *likely* to act as prey or predators respectively. That is, there is a statistical trophic structure to the ecosystem represented by these matrices.

Starting from a given Gaussian ensemble, we now use the universality discussed in the previous section, to find matrices which enforce a given set of sign relations *and* produce the same eigenvalue spectrum as the Gaussian ensemble. We impose a $(-,+)$ relation for pairs in which one species belongs to the prey block and the other to the predator block. Further, we eliminate intraguild predation by constraining the interaction coefficients between two prey species (or two predator species) all to have the same sign (positive for prey-prey interactions and negative for predator-predator in the example below). Here, for the sake of the analysis, we also constrain $\mu_{uv} = -\mu_{vu}$, $\Gamma_{uv} < 0$, $\Gamma_{uu} > 0$ and $\Gamma_{vv} > 0$. A similar procedure could in principle be repeated for other set-ups [for example where prey-prey interactions were not constrained always to be $(+,+)$ but could also be $(-,-)$].

A natural starting point for the construction of such a matrix is a distribution with a support only on the positive (or negative) half of the real axis, guaranteeing the sign of the random variable. One such distribution is the gamma distribution. Consider a pair of independent gamma-distributed random variables $x_{ij}^{\alpha\beta} > 0$ and $y_{ij}^{\alpha\beta} > 0$, each distributed according to

$$p(\xi) = \frac{1}{\Gamma(k_{\alpha\beta})\theta_{\alpha\beta}^{k_{\alpha\beta}}} \xi^{k_{\alpha\beta}-1} e^{-\xi/\theta_{\alpha\beta}} \tag{S110}$$

for non-negative ξ, where $\Gamma(k)$ here indicates the standard gamma function (not to be confused with the correlation coefficients Γ_{uu} etc.). The mean and variance of this distribution are

$$\begin{aligned} \langle \xi \rangle &= k_{\alpha\beta}\theta_{\alpha\beta}, \\ \langle (\xi - \langle \xi \rangle)^2 \rangle &= k_{\alpha\beta}\theta_{\alpha\beta}^2. \end{aligned} \tag{S111}$$

Now focus on a given pair of species i and j, where i is in block $\alpha \in \{u, v\}$, and j in block $\beta \in \{u, v\}$. The diagonally opposing entries in the random matrix describing the interactions between these two species are then constructed as linear combinations of $x_{ij}^{\alpha\beta}$ and $y_{ij}^{\alpha\beta}$,

$$\begin{aligned} m_{ij}^{\alpha\beta} &= a_{xx}^{\alpha\beta} x_{ij}^{\alpha\beta} + a_{xy}^{\alpha\beta} y_{ij}^{\alpha\beta}, \\ m_{ji}^{\beta\alpha} &= a_{yx}^{\alpha\beta} x_{ij}^{\alpha\beta} + a_{yy}^{\alpha\beta} y_{ij}^{\alpha\beta}. \end{aligned} \tag{S112}$$

It can then be verified that the following choice for the coefficients in the linear transformation produces the correct signs, moments and correlations for the matrix elements $m_{ij}^{\alpha\beta}$,

$$\begin{aligned} a_{xx}^{\alpha\beta} &= (-1)^{\delta_{\beta v}} \left[\sqrt{1 + \Gamma_{\alpha\beta}} + \sqrt{1 - \Gamma_{\alpha\beta}} \right], \\ a_{xy}^{\alpha\beta} &= (-1)^{\delta_{\beta v}} (-1)^{1-\delta_{\alpha\beta}} \left[\sqrt{1 + \Gamma_{\alpha\beta}} - \sqrt{1 - \Gamma_{\alpha\beta}} \right], \\ a_{yx}^{\alpha\beta} &= (-1)^{\delta_{\alpha v}} (-1)^{1-\delta_{\alpha\beta}} \left[\sqrt{1 + \Gamma_{\alpha\beta}} - \sqrt{1 - \Gamma_{\alpha\beta}} \right], \\ a_{yy}^{\alpha\beta} &= (-1)^{\delta_{\alpha v}} \left[\sqrt{1 + \Gamma_{\alpha\beta}} + \sqrt{1 - \Gamma_{\alpha\beta}} \right], \end{aligned} \tag{S113}$$

if we choose $k_{\alpha\beta} = \frac{(\mu'_{\alpha\beta})^2}{N\sigma^2}$, $\theta_{\alpha\beta} = \frac{\sigma^2}{\mu'_{\alpha\beta}}$, where $\mu'_{\alpha\beta} = \frac{|\mu_{\alpha\beta}|}{\sqrt{1+\Gamma_{\alpha\beta}}}$. The exponents on the right-hand side of Eq. (S113) indicate Kronecker deltas, for example $\delta_{\beta v} = 1$ if $\beta = v$ (species j is in the predator block), and $\delta_{\beta v} = 0$ if $\beta = u$ (species j is in the prey block).

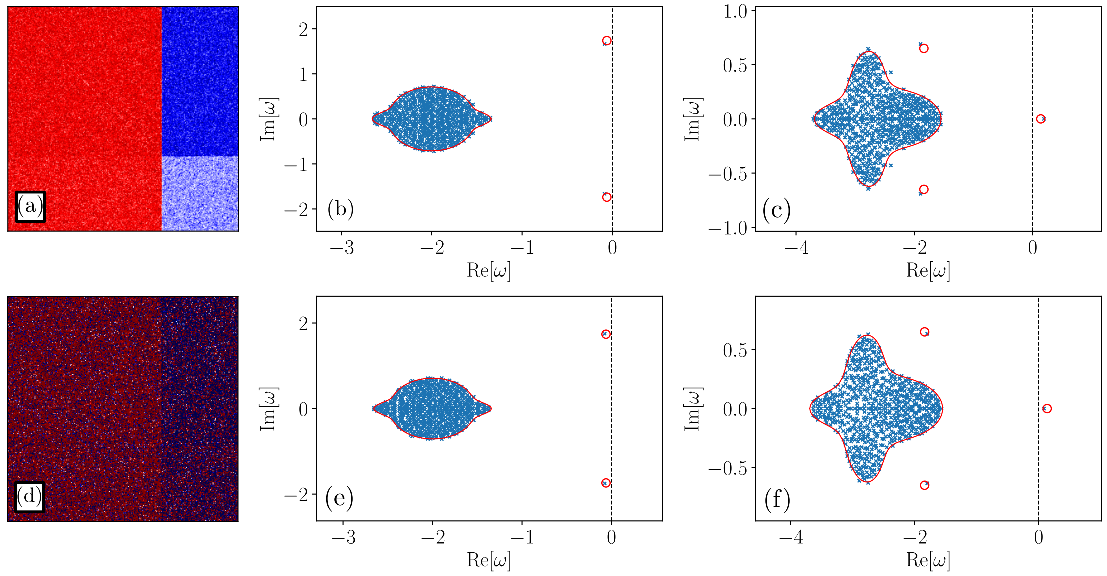

FIG. S3. **Universality allows the application of the Gaussian theory to a model with strict trophic interactions.** Comparison between the eigenvalue spectra of random matrices with entries drawn from a gamma distribution and a Gaussian distribution. Panels (a) and (d) show the entries of the matrix $m_{ij}^{\alpha\beta}$ with red indicating a positive entry and blue a negative one. In panels (a), (b) and (c), random matrix entries are constrained to have signs befitting the trophic level to which they belong. In panels (d), (e) and (f), random matrix elements are drawn from a Gaussian distribution and so do not necessarily obey the sign relations, although they have a statistical tendency to do so. The random matrix entries in (a) and (d) are drawn from distribution which have the same moments, so the spectra in panels (b)/(c) and (e)/(f), respectively, are statistically equivalent. The only difference between (b) and (c) [(e) and (f)] is the value of q, affecting only the diagonal elements of $\mathbf{M}_q$. The figure demonstrates that the dispersal-induced instability remains possible for a model with strict trophic levels. The system parameters (after rescaling, see Sec. S4) here are $D_u = 1$, $D_v = 5$, $NC\mu_{uu} = NC\mu_{vu} = -NC\mu_{uv} = 8$, $NC\mu_{vv} = -4$, $\gamma_u = 2/3$, $\gamma_v = 1/3$, $c = NC\sigma^2 = 0.375$, $d_u = d_v = 2$, $N = 750$, $C = 1$, $\Gamma_u = 0.5$, $\Gamma_{uv} = -0.9$, $\Gamma_v = 0.6$. Panels (b) and (e) $q = 0$, panels (c) and (f) $q = 0.5$.

That the correct signs of the elements $m_{ij}^{\alpha\beta}$ are upheld by Eq. (S113) can be seen case-by-case as follows. We note that in $\sqrt{1+\Gamma_{\alpha\beta}} + \sqrt{1-\Gamma_{\alpha\beta}} > 0$ trivially in all cases. Take for example the case $\alpha = \beta = u$. Then we require all $a_{\gamma\delta}^{uu}$ to be positive. Since we constrain $\Gamma_{uu} > 0$, $\sqrt{1+\Gamma_{uu}} > \sqrt{1-\Gamma_{uu}}$. All the powers of the factors of -1 in the prefactors are zero in this case and hence all the coefficients $a_{\gamma\delta}^{uu}$ are positive. In the case, $\alpha = \beta = v$, the coefficients must all be negative. Again, since $\Gamma_{vv} > 0$, we have $\sqrt{1+\Gamma_{vv}} > \sqrt{1-\Gamma_{vv}}$ so the signs of the coeffcients $a_{\gamma\delta}^{\alpha\beta}$ are determined by the prefactors, which one can see each yield -1 in this case. In the case $\alpha = u$ and $\beta = v$, we require $a_{xx}^{uv} < 0$, $a_{xy}^{uv} < 0$, $a_{yx}^{uv} > 0$ and $a_{yy}^{uv} > 0$. Now, since $\Gamma_{uv} < 0$, we have $\sqrt{1+\Gamma_{\alpha\beta}} < \sqrt{1-\Gamma_{\alpha\beta}}$. The prefactor of a_{xx}^{uv} is -1 and that of a_{xy}^{uv} is 1 – both coefficients

are negative. In contrast, the prefactor of a^{uv}_{yx} is -1 and that of a^{uv}_{yy} is 1 and both coefficients are positive. A similar outcome is true for the case $\alpha = v$ and $\beta = u$. This scheme is verified in Fig. S3(a).

We can thus produce random matrices whose elements are certain to have particular signs. This sign constraint still permits us to vary the statistics of the random matrix elements. As is shown in Fig. S3, one can reproduce the eigenvalue spectrum of a Gaussian random matrix with such a sign-constrained random matrix. This speaks to the validity of the block structure of the random matrices as a representation of trophic structure in an ecosystem. Conversely, we note that given a model with strictly enforced signs in each block of the matrix, one can find a Gaussian ensemble with the same first and second moments. One can then use the theory to compute the eigenvalue support and stability.

S9. VARIATION IN DIAGONAL ELEMENTS

In our original model, following May [S32], we took the diagonal elements of the interaction matrix to be constant within the groups of predator and prey species for the sake of mathematical simplicity. We now relax this assumption and allow these diagonal elements to vary. We extend our analytical results for the boundary of the bulk region of the eigenvalue spectrum and the locations of any outlier eigenvalues, taking this variation in diagonal entries into account. We then discuss the effect on stability when the self-regulation rates or the dispersal coefficients are allowed to vary.

A. Boundary of the bulk of the eigenvalue spectrum

We begin by returning to the model used in the main text [see also Eq. (S32)], making the alteration $d_\alpha \to d_i^\alpha$. That is, the diagonal elements may now vary from species to species. This formulation also allows us to study the effect of variation in the dispersal coefficients D_α on stability, as we discuss in Section S9 E.

The diagonal elements d_i^α are now drawn from an as-yet-unspecified distribution. Eq. (S32) becomes

$$\begin{aligned}\exp\left[-N\Phi(\omega)\right] = \int \prod_{i\alpha}\left[\left(\frac{d^2 z_i^\alpha d^2 y_i^\alpha}{2\pi^2}\right)\right]\exp\left[-\sum_{i\alpha} y_i^{\alpha\star}y_i^\alpha + \epsilon z_i^{\alpha\star}z_i^\alpha\right] \\ \times\exp\left[-i\sum_{i\alpha} z_i^{\alpha\star}y_i^\alpha(\omega^\star + d_i^\alpha) + z_i^\alpha y_i^{\alpha\star}(\omega + d_i^\alpha)\right] \\ \times\exp\left[-\frac{\sigma^2}{2N}\sum_{ij\alpha\beta}(z_i^{\alpha\star}y_j^\beta + z_i^\alpha y_j^{\beta\star})^2 + \Gamma_{\alpha\beta}(z_i^{\alpha\star}y_j^\beta + z_i^\alpha y_j^{\beta\star})(z_j^{\beta\star}y_i^\alpha + z_j^\beta y_i^{\alpha\star})\right].\end{aligned} \tag{S114}$$

We once again introduce the order parameters

$$\begin{aligned} u &= \frac{1}{N}\sum_{i\alpha} z_i^{\alpha\star}z_i^\alpha, \quad v = \frac{1}{N}\sum_{i\alpha} y_i^{\alpha\star}y_i^\alpha, \\ w_\alpha &= \frac{1}{N_\alpha}\sum_i z_i^{\alpha\star}y_i^\alpha, \quad w_\alpha^\star = \frac{1}{N_\alpha}\sum_i y_i^{\alpha\star}z_i^\alpha, \end{aligned} \tag{S115}$$

and we arrive at

$$\exp\left[-N\Phi(\omega)\right] = \int \mathcal{D}\left[\cdots\right] \exp\left[N(\Psi+\Theta+\Omega)\right], \tag{S116}$$

where $\mathcal{D}[\cdots]$ denotes integration over all of the order parameters and their conjugate ('hatted') variables, and where

$$\Psi = i\hat{u}u + i\hat{v}v + i\sum_\alpha \gamma_\alpha(\hat{w}_\alpha w_\alpha^\star + \hat{w}_\alpha^\star w_\alpha),$$

$$\Theta = -\epsilon u - v - \sigma^2 uv + \sum_\alpha \gamma_\alpha\left[-iw_\alpha\omega^\star - iw_\alpha^\star\omega\right] - \frac{1}{2}\sigma^2\sum_{\alpha\beta}\gamma_\alpha\gamma_\beta\Gamma_{\alpha\beta}(w_\alpha w_\beta + w_\alpha^\star w_\beta^\star),$$

$$\Omega = \frac{1}{N}\sum_{\alpha,i}\gamma_\alpha \ln\left[\int\left[\left(\frac{d^2z_i^\alpha d^2y_i^\alpha}{2\pi^2}\right)\right]\exp\left\{-i[\hat{u}z_i^{\alpha\star}z_i^\alpha + \hat{v}y_i^{\alpha\star}y_i^\alpha + (\hat{w}_\alpha + d_i^\alpha)y_i^{\alpha\star}z_i^\alpha + (\hat{w}_\alpha^\star + d_i^\alpha)z_i^{\alpha\star}y_i^\alpha]\right\}\right]$$

$$\approx \sum_\alpha \gamma_\alpha \int d(d^\alpha)P(d^\alpha)\ln\Bigg\{\int\left[\left(\frac{d^2z^\alpha d^2y^\alpha}{2\pi^2}\right)\right]$$
$$\times\exp\left\{-i[\hat{u}z^{\alpha\star}z^\alpha + \hat{v}y^{\alpha\star}y^\alpha + (\hat{w}_\alpha + d^\alpha)y^{\alpha\star}z^\alpha + (\hat{w}_\alpha^\star + d^\alpha)z^{\alpha\star}y^\alpha]\right\}\Bigg\}. \tag{S117}$$

Once again the integrals over y_i^α and z_i^α can be carried out, yielding

$$\begin{aligned}\Omega &= -\sum_\alpha\gamma_\alpha\int d(d^\alpha)P_\alpha(d^\alpha)\ln\left\{(\hat{w}_\alpha + d^\alpha)(\hat{w}_\alpha^\star + d^\alpha) - \hat{u}\hat{v}\right\}\\ &\equiv -\sum_\alpha\gamma_\alpha\left\langle\ln\left\{(\hat{w}_\alpha + d^\alpha)(\hat{w}_\alpha^\star + d^\alpha) - \hat{u}\hat{v}\right\}\right\rangle_d.\end{aligned} \tag{S118}$$

The notation $\langle\cdots\rangle_d$ indicates an average over the distribution $P_\alpha(d_\alpha)$ of diagonal elements. At the saddle point, we therefore obtain by differentiating Eq. (S117) with respect to the 'hatted' quantities [c.f. Eqs. (S38)]

$$\begin{aligned} iu = -\sum_\alpha\gamma_\alpha\left\langle\frac{\hat{v}}{(\hat{w}_\alpha + d_\alpha)(\hat{w}_\alpha^\star + d_\alpha) - \hat{u}\hat{v}}\right\rangle_d, &\quad iv = -\sum_\alpha\gamma_\alpha\left\langle\frac{\hat{u}}{(\hat{w}_\alpha + d_\alpha)(\hat{w}_\alpha^\star + d_\alpha) - \hat{u}\hat{v}}\right\rangle_d,\\ iw_\alpha^\star = \left\langle\frac{\hat{w}_\alpha^\star + d_\alpha}{(\hat{w}_\alpha + d_\alpha)(\hat{w}_\alpha^\star + d_\alpha) - \hat{u}\hat{v}}\right\rangle_d, &\quad iw_\alpha = \left\langle\frac{\hat{w}_\alpha + d_\alpha}{(\hat{w}_\alpha + d_\alpha)(\hat{w}_\alpha^\star + d_\alpha) - \hat{u}\hat{v}}\right\rangle_d.\end{aligned} \tag{S119}$$

We now define the following

$$\hat{f} = \sum_\alpha\gamma_\alpha\left\langle\frac{1}{(\hat{w}_\alpha + d_\alpha)(\hat{w}_\alpha^\star + d_\alpha) - \hat{u}\hat{v}}\right\rangle_d, \quad \hat{d}_\alpha = \frac{1}{\hat{f}}\left\langle\frac{d_\alpha}{(\hat{w}_\alpha + d_\alpha)(\hat{w}_\alpha^\star + d_\alpha) - \hat{u}\hat{v}}\right\rangle_d. \tag{S120}$$

Thus,

$$\begin{aligned} iu = -\hat{v}\hat{f}, &\quad iv = -\hat{u}\hat{f},\\ iw_\alpha^\star = (\hat{w}_\alpha^\star + \hat{d}_\alpha)\hat{f}, &\quad iw_\alpha = (\hat{w}_\alpha + \hat{d}_\alpha)\hat{f}.\end{aligned} \tag{S121}$$

We also obtain the complementary set of saddle-point relations by differentiating Eq. (S117) with respect to the 'unhatted' quantities [c.f. Eqs. (S43)]

$$i\hat{u} = \epsilon + \sigma^2 v, \quad i\hat{v} = 1 + \sigma^2 u,$$

$$i\hat{w}_\alpha = i\omega + \sigma^2 \sum_\beta \gamma_\beta \Gamma_{\alpha\beta} w^\star_\beta,$$
$$i\hat{w}^\star_\alpha = i\omega^\star + \sigma^2 \sum_\beta \gamma_\beta \Gamma_{\alpha\beta} w_\beta. \tag{S122}$$

We now employ a slightly different solution strategy to previous calculations, opting instead to leave the solution in terms of the hatted order parameters. For $\epsilon \to 0^+$ we see that

$$\hat{u} = \sigma^2 \hat{u} \hat{f}. \tag{S123}$$

This has two solutions – either $\hat{u} = 0$, in which case the resolvent $iw^\star$ is analytic and the eigenvalue density vanishes, or $\hat{f} = \frac{1}{\sigma^2}$. For the boundary of the support of the eigenvalue spectrum, we solve for the values of ω which satisfy both conditions simultaneously. That is, we solve

$$\hat{f} = \sum_\alpha \gamma_\alpha \left\langle \frac{1}{(\hat{w}_\alpha + d_\alpha)(\hat{w}^\star_\alpha + d_\alpha)} \right\rangle_d = \frac{1}{\sigma^2},$$
$$\hat{d}_\alpha = \sigma^2 \left\langle \frac{d_\alpha}{(\hat{w}_\alpha + d_\alpha)(\hat{w}^\star_\alpha + d_\alpha)} \right\rangle_d,$$
$$\hat{w}_\alpha = \omega - \sum_\beta \gamma_\alpha \gamma_\beta \Gamma_{\alpha\beta} (\hat{w}^\star_\beta + \hat{d}_\beta),$$
$$\hat{w}^\star_\alpha = \omega^\star - \sum_\beta \gamma_\alpha \gamma_\beta \Gamma_{\alpha\beta} (\hat{w}_\beta + \hat{d}_\beta). \tag{S124}$$

We note that the boundary of the eigenvalue spectrum is dependent on the shape of the probability distribution from which we draw the diagonal elements. This is in contrast to the universality property for the off-diagonal random matrix elements discussed in Section S7.

Recovering the results of Ref. [S14]

Eqs. (S124) simplify in the case $\gamma_u = 1$ to results previously obtained in Ref. [S14], which were obtained using a different method (that of quaternionic resolvents). Letting $iw^\star_u = \alpha(-\omega)$ and suppressing the subscript u for brevity, one deduces from the first and third of Eqs. (S124) and the third of Eqs. (S121)

$$\left\langle \left[|d - \omega - \Gamma\sigma^2 \alpha(\omega)|^2 \right]^{-1} \right\rangle_d = \frac{1}{\sigma^2}. \tag{S125}$$

Now using the fourth of Eqs. (S124) in combination with the third of Eqs. (S121) one obtains

$$\sigma^2 \alpha(\omega) - \hat{d} = -\omega^\star - \Gamma\sigma^2 \alpha^\star(\omega). \tag{S126}$$

Letting $\alpha(\omega) = \alpha_{\mathrm{re}} + i\alpha_{\mathrm{im}}$ and $\omega = \omega_x + i\omega_y$, the imaginary part of this equation obeys

$$\alpha_{\mathrm{im}} = \frac{\omega_y}{\sigma^2(1-\Gamma)}, \tag{S127}$$

and the real part obeys

$$\sigma^2 \alpha_{\mathrm{re}} = \left\langle \left[d - \sigma^2 \omega_x - \Gamma\sigma^4 \alpha_{\mathrm{re}} \right] \left[|d - \omega - \Gamma\sigma^2 \alpha(\omega)|^2 \right]^{-1} \right\rangle_d, \tag{S128}$$

where we have used Eq. (S125) and the second of Eqs. (S124). Writing the averages over d explicitly in Eqs. (S128) and (S125), one obtains

$$\frac{1}{\sigma^2} = \int d(d)P(d)\frac{1}{[d-\omega_x-\Gamma\sigma^2\alpha_{\rm re}]^2+\omega_y^2/(1-\Gamma)^2},$$
$$\sigma^2\alpha_{\rm re} = \int d(d)P(d)\frac{d-\sigma^2\omega_x-\Gamma\sigma^4\alpha_{\rm re}}{[d-\omega_x-\Gamma\sigma^2\alpha_{\rm re}]^2+\omega_y^2/(1-\Gamma)^2}. \tag{S129}$$

These are exactly Eqs. (4.42) and (4.43) in the Supplement of Ref. [S14] (once one sets $\sigma^2 = 1$ in the above equations and takes the limit $|\beta| \to 0$ in those of Ref. [S14] so as yield the equations for the boundary of the support).

B. Diagonal variation stretches the bulk support along the real axis

In order provide and example and to compare to previous work [S14], we take the case of a matrix without trophic structure. When the diagonal elements are uniformly distributed between $d_0 - \Delta_0/2$ and $d_0 + \Delta_0/2$ one finds from the first two of Eqs. (S124) (noting that $\hat{w}$ and $\hat{w}^\star$ are complex conjugates and defining $\hat{w} = \hat{w}_x + i\hat{w}_y$)

$$\hat{f} = \frac{1}{\hat{w}_y\Delta_0}\left[\arctan\left(\frac{\hat{w}_x+d_0+\Delta_0/2}{\hat{w}_y}\right) - \arctan\left(\frac{\hat{w}_x+d_0-\Delta_0/2}{\hat{w}_y}\right)\right],$$
$$\frac{\Delta_0\hat{d}}{\sigma^2} = \frac{1}{2}\ln\left[\frac{(\hat{w}_x+d_0+\Delta_0)^2+\hat{w}_y^2}{(\hat{w}_x+d_0-\Delta_0)^2+\hat{w}_y^2}\right] - \frac{\hat{w}_x\Delta_0}{\sigma^2}. \tag{S130}$$

In the case where $\Gamma = 0$, from last two of Eqs. (S124) we have $\hat{w} = \omega \equiv \omega_x + i\omega_y$ and we obtain the following closed form solution for the boundary of the support

$$\frac{1}{\omega_y\Delta_0}\left[\arctan\left(\frac{\omega_x+d_0+\Delta_0/2}{\omega_y}\right) - \arctan\left(\frac{\omega_x+d_0-\Delta_0/2}{\omega_y}\right)\right] = \frac{1}{\sigma^2}, \tag{S131}$$

which can be written alternatively

$$\frac{1}{\omega_y\Delta_0}\arctan\left[\frac{\Delta_0\omega_y}{(\omega_x+d_0)^2+\omega_y^2-\frac{\Delta_0^2}{4}}\right] = \frac{1}{\sigma^2}. \tag{S132}$$

One notes that for small Δ_0 this reduces to the circular law. We can solve this exactly when $\omega_y \to 0$. In this case, one finds

$$\omega_x = -d_0 \pm \sqrt{\sigma^2 + \frac{\Delta_0^2}{4}}. \tag{S133}$$

This agrees with the result for the abscissa derived in Ref. [S14] (see Eq. (5.13) in the Supplementary Material of Ref. [S14]).

Eq. (S133) indicates that the variation in diagonal elements acts to elongate the bulk region of the eigenvalue spectrum. This is demonstrated in Fig. S4.

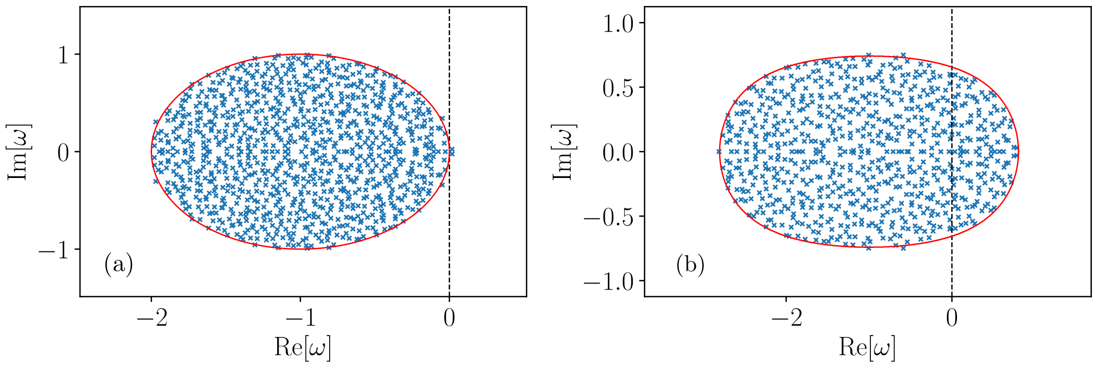

FIG. S4. **Destabilising effect of heterogeneity in diagonal matrix elements.** Eigenvalue spectrum for $NC\mu_{uu} = 0$, $NC\mu_{uv} = 0$, $NC\mu_{vu} = 0$, $NC\mu_{vv} = 0$, $c = NC\sigma^2 = 1$, $\Gamma_{uv} = 0$, $\Gamma_{uu} = 0$, $\Gamma_{vv} = 0$, $\gamma_u = 1$, $\gamma_v = 0$, $C = 1$ and $N = 750$. The diagonal entries are uniformly distributed with parameters $d_0 = 1$ and (a) $\Delta_0 = 0$, (b) $\Delta_0 = 3$.

C. Calculation of outlier eigenvalues

The saddle-point approximation to the resolvent $iw_\alpha^\star$ can be extracted from Eqs. (S124) using Eq. (S121). From this, the outlier eigenvalues which arise from the inclusion of non-zero random matrix entry means $\mu_{\alpha\beta}$ can be deduced as usual. The outlier eigenvalues λ must satisfy, as in Section S3,

$$\left[\gamma_u \mu_{uu} - \frac{1}{\chi_u(\lambda)}\right]\left[\gamma_v \mu_{vv} - \frac{1}{\chi_v(\lambda)}\right] - \gamma_u \gamma_v \mu_{uv}\mu_{vu} = 0. \tag{S134}$$

However, with the inclusion of variation in the diagonal elements, the quantities $\chi_\alpha(\lambda)$ now satisfy

$$\begin{aligned}
\chi_\alpha &= (\hat{w}_\alpha^\star + \hat{d}_\alpha)\hat{f}, \\
\hat{f} &= \sum_\alpha \gamma_\alpha \left\langle \frac{1}{(\hat{w}_\alpha + d_\alpha)(\hat{w}_\alpha^\star + d_\alpha)} \right\rangle_d, \\
\hat{d}_\alpha &= \frac{1}{\hat{f}} \left\langle \frac{d_\alpha}{(\hat{w}_\alpha + d_\alpha)(\hat{w}_\alpha^\star + d_\alpha)} \right\rangle_d, \\
\hat{w}_\alpha &= \lambda - \sum_\beta \gamma_\beta \Gamma_{\alpha\beta}(\hat{w}_\beta^\star + \hat{d}_\beta), \\
\hat{w}_\alpha^\star &= \lambda^\star - \sum_\beta \gamma_\beta \Gamma_{\alpha\beta}(\hat{w}_\beta + \hat{d}_\beta),
\end{aligned} \tag{S135}$$

subject to the condition

$$\sum_\alpha \gamma_\alpha |\chi_\alpha(\lambda)|^2 < \frac{1}{\sigma^2}. \tag{S136}$$

Example: Random matrix without trophic structure

We once again take a simple example to verify this approach. The equations for the outlier simplify greatly in the case where $\gamma_v = 0$ and $\Gamma_{\alpha\beta} = 0$ (i.e. where there is no trophic structure or

correlations). We find

$$\lambda^\star = -\hat{d} + \frac{1}{\mu \hat{f}},$$
$$\hat{f} = \left\langle \frac{1}{(\lambda + d)(\lambda^\star + d)} \right\rangle_d,$$
$$\hat{d} = \frac{1}{\hat{f}} \left\langle \frac{d}{(\lambda + d)(\lambda^\star + d)} \right\rangle_d. \tag{S137}$$

Taking the example once again of the uniform distribution for the diagonal elements centred on d_0 with width Δ_0, one obtains for the last two of Eqs. (S137)

$$\hat{f} = \frac{1}{\lambda_y \Delta_0} \arctan\left[\frac{\Delta_0 \lambda_y}{(\lambda_x + d_0)^2 + \lambda_y^2 - \frac{\Delta_0^2}{4}}\right],$$
$$\Delta_0 \hat{f}\hat{d} = \frac{1}{2} \ln\left[\frac{(\lambda_x + d_0 + \Delta_0/2)^2 + \lambda_y^2}{(\lambda_x + d_0 - \Delta_0/2)^2 + \lambda_y^2}\right] - \lambda_x \Delta_0 \hat{f}. \tag{S138}$$

Presuming that the outlier is real, we find

$$\hat{f} = \frac{1}{(\lambda_x + d_0)^2 - \Delta_0^2/4},$$
$$\hat{d} = \frac{1}{\Delta_0 \hat{f}} \ln\left[\frac{(\lambda_x + d_0 + \Delta_0/2)}{(\lambda_x + d_0 - \Delta_0/2)}\right] - \lambda_x. \tag{S139}$$

Hence, combining this with the first of Eqs. (S137)

$$\lambda_x = -d_0 + \frac{\Delta_0}{2} \frac{\exp[\Delta_0/\mu] + 1}{\exp[\Delta_0/\mu] - 1}. \tag{S140}$$

This expression is verified in Fig. S5.

When $\Delta_0 \to 0$, we recover $\lambda_x = -d_0 + \mu$. As $\Delta_0 \to \infty$, $\lambda_x \approx -d_0 + \Delta_0/2$ for $\mu > 0$ and $\lambda_x \approx -d_0 - \Delta_0/2$ for $\mu < 0$. So variation in the diagonal entries can act to change the outlier eigenvalues considerably, depending on the degree of variation.

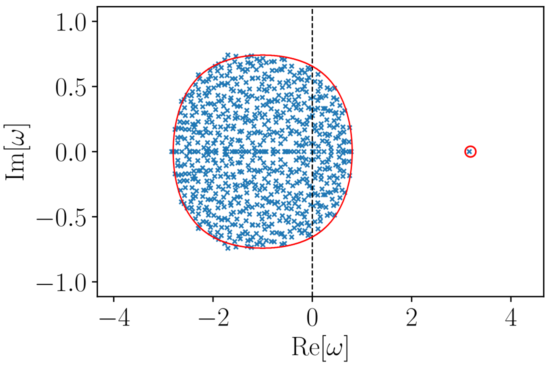

FIG. S5. **Verification of theoretical predictions for outlier eigenvalues of matrices with heterogeneous diagonal elements.** Eigenvalue spectrum for $NC\mu_{uu} = 4$, $NC\mu_{uv} = 4$, $NC\mu_{vu} = 4$, $NC\mu_{vv} = 4$, $c = NC\sigma^2 = 1.0$, $\Gamma_{uv} = 0$, $\Gamma_{uu} = 0$, $\Gamma_{vv} = 0$, $\gamma_u = 1$, $\gamma_v = 0$, $C = 1$ and $N = 750$. The diagonal entries are uniformly distributed with parameters $d_0 = 1$ and $\Delta_0 = 3$.

D. Dispersal-induced instability remains possible in models with trophic structure when self-interaction is heterogeneous

We now turn our attention to whether or not variation in the diagonal elements disrupts the possibility of a Turing instability. To do this, we return to the model with trophic structure. We once again take the simple case of $\Gamma_{\alpha\beta} = 0$. In this case, from Eq. (S124) we find the following closed-form solution for the boundary of the bulk region of the eigenvalue spectrum

$$\sum_{\alpha} \frac{\gamma_\alpha}{\omega_y \Delta_0^\alpha} \arctan\left[\frac{\Delta_0^\alpha \omega_y}{(\omega_x + d_0^\alpha)^2 + \omega_y^2 - \frac{(\Delta_0^\alpha)^2}{4}}\right] = \frac{1}{\sigma^2}. \tag{S141}$$

From Eqs. (S135), we see that the outlier eigenvalues obey

$$\begin{aligned} 0 &= \left[\gamma_u \mu_{uu} - \frac{1}{\chi_u(\lambda)}\right]\left[\gamma_v \mu_{vv} - \frac{1}{\chi_v(\lambda)}\right] - \gamma_u\gamma_v\mu_{uv}\mu_{vu}, \\ \chi_\alpha(\lambda_x, \lambda_y) &= (\lambda_x + \hat{d}_\alpha)\hat{f} - i\lambda_y \hat{f}, \\ \hat{f} &= \sum_\alpha \frac{\gamma_\alpha}{\lambda_y \Delta_0^\alpha} \arctan\left[\frac{\Delta_0^\alpha \lambda_y}{(\lambda_x + d_0^\alpha)^2 + \lambda_y^2 - \frac{(\Delta_0^\alpha)^2}{4}}\right], \\ \hat{d}_\alpha &= \frac{1}{2\Delta_0^\alpha \hat{f}} \ln\left[\frac{(\lambda_x + d_0^\alpha + \Delta_0^\alpha/2)^2 + \lambda_y^2}{(\lambda_x + d_0^\alpha - \Delta_0^\alpha/2)^2 + \lambda_y^2}\right] - \lambda_x, \end{aligned} \tag{S142}$$

where $\lambda = \lambda_x + i\lambda_y$. These equations can be evaluated numerically. We find that despite considerable diagonal variation, the Turing instability remains possible, as is demonstrated in Fig. S6.

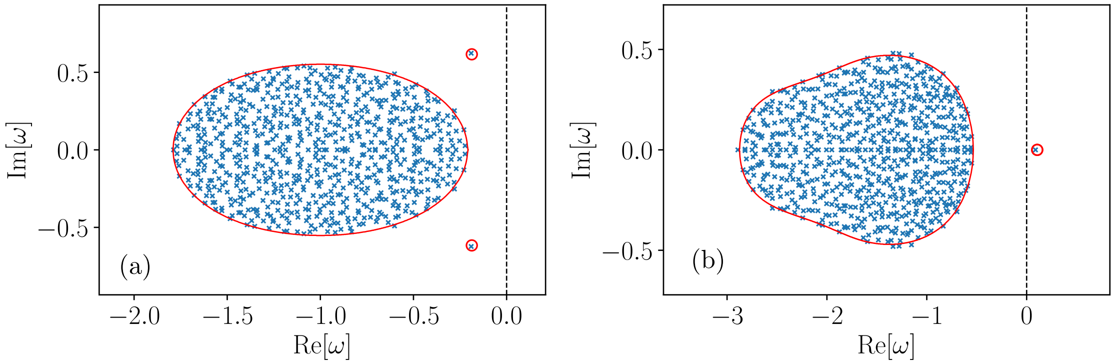

FIG. S6. **Example of dispersal-induced instability when self-interaction coefficients are heterogeneous.** Eigenvalue spectra for random matrices with trophic structure and statistical variation in the diagonal elements. The system parameters (after rescaling, see Sec. S4) here are $D_u = 1$, $D_v = 5$, $NC\mu_{uu} = NC\mu_{vu} = -NC\mu_{uv} = 3$, $NC\mu_{vv} = -1.5$, $\gamma_u = 2/3$, $\gamma_v = 1/3$, $c = NC\sigma^2 = 0.375$, $d_0^u = d_0^v = 1$, $\Delta_0^u = \Delta_0^v = 1$. Panel (a) $q = 0$, panel (b) $q = 1$.

E. Variation in dispersal coefficients can promote dispersal-induced instability

Now we imagine that instead of the intra-species auto-repression rates d^α, the dispersal rates D^α vary. We assume they are distributed uniformally between $D_0^\alpha - \Delta_0^\alpha/2$ and $D_0^\alpha + \Delta_0^\alpha/2$. This

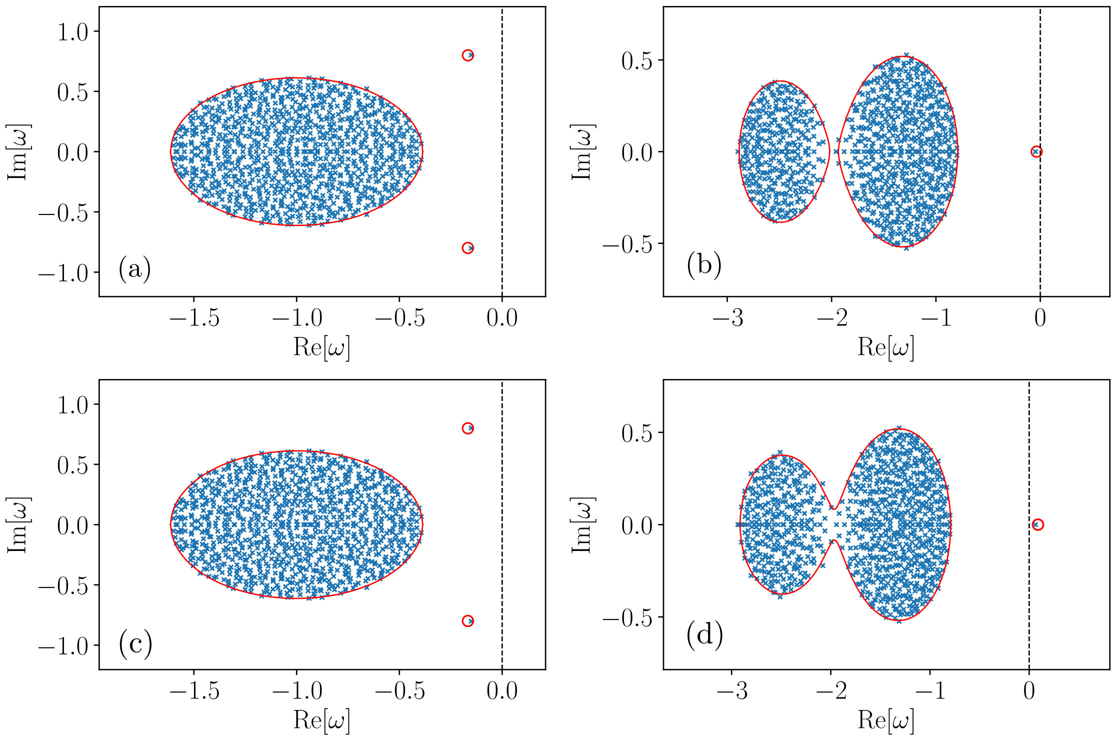

FIG. S7. **Example of dispersal-induced instability when diffusion coefficients are heterogeneous.** Eigenvalue spectra for random matrices with trophic structure and statistical variation in the dispersal coefficients. (a) Variation in dispersal coefficients has no effect on the $q = 0$ mode. In (b) there is no variation in dispersal coefficients but in (c) there is. In going from (b) to (c), the bulk regions of the spectrum are elongated in the horizontal direction and the outlier is made more unstable. The system parameters (after rescaling, see Sec. S4) here are $D_u = 1$, $D_v = 5$, $NC\mu_{uu} = NC\mu_{vu} = -NC\mu_{uv} = 3$, $NC\mu_{vv} = -1.5$, $\gamma_u = 2/3$, $\gamma_v = 1/3$, $c = NC\sigma^2 = 0.375$, $d_u = d_v = 1$. The top two panels have $\Delta_0^u = \Delta_0^v = 0$ and the bottom have $\Delta_0^u = \Delta_0^v = 1$. Panels (a) and (c) $q = 0$, panels (b) and (d) $q = 1$.

is a fairly minor change to make with respect to the preceding analysis, the only difference being that now the degree of diagonal variation depends on q^2. In Eqs. (S130), for example, one simply makes the replacement $d_0 \to d_0 + q^2 D_0$ and $\Delta_0 \to q^2\Delta_0$. In Fig. S7 we show eigenvalue spectra for the case of varying dispersal rates. As can be seen variation in the dispersal rates can promote dispersal-induced instability.

Part III

Turing instability and the stabilising effects of spatial heterogeneity

In this part of the Supplement we investigate the effects of spatial heterogeneity. To do this, we allow the interaction matrix to vary across different points in space. Such a setup was considered by Gravel, Massol and Leibold ('GML') in a model without trophic structure and with all-to-all dispersal between a set of discrete patches [S1]. GML report that this results in a stabilising effect for the bulk eigenvalues. The focus of our work is on dispersal-induced instability in complex ecosystems with trophic structure. We therefore ask how spatial heterogeneity impacts such an instability.

To answer this question we construct a model which combines the features described in the main text and those in GML. That is, we consider the combination of dispersal, spatial heterogeneity in the interaction coefficients, and trophic structure. We detail how the same analytical approach used to produce the results in the main text can be applied in this case. The definition of the combined model and the subsequent calculations can be found in Section S10. In Section S11 we demonstrate that this combined set-up contains the model in the main text and that of [S1] as limiting cases. In particular, we rederive the stability criteria reported in GML and observe the same stabilising effect when heterogeneity and dispersal are combined.

In Section S12 we use this set-up to show that the stabilising mechanism of GML is largely independent of the way dispersal is implemented; that is, whether dispersal is modelled by local or 'all-to-all' hopping between patches. We also introduce a non-zero mean to the random matrix elements in GML's model and demonstrate that outlier eigenvalues are not necessarily subject to the stabilising mechanism reported by GML for the bulk eigenvalues.

Conversely, in Section S13 we show that dispersal-induced instability persists if the dispersal in the model in the main text is changed from local diffusion in continuous space to non-local 'all-to-all' hopping amongst a set of patches (as it is in GML). This further demonstrates that the dispersal-induced effects under consideration are robust under changes in the implementation of dispersal.

Finally, in Section S14, we analyse a setup combining the features of the model in Section S1 and that of GML, i.e., trophic structure, spatial heterogeneity and dispersal. We illustrate that both the stabilising and destabilising dispersal-induced effects can be seen for different sets of model parameters. That is, the two effects can coexist within the same model. We then proceed to identify the roles of the different model parameters in promoting dispersal-induced stabilisation or destabilisation.

S10. SPATIAL HETEROGENEITY IN INTERACTION COEFFICIENTS

A. Model setup

In this section, we construct and analyse a model involving trophic structure, spatial heterogeneity of interactions and dispersal. This allows us to investigate the interplay between the Turing instability induced by outlier eigenvalues, and the stabilising mechanism for bulk eigenvalues reported in Ref. [S1].

The model involves n discrete 'patches', indexed by their spatial coordinate x. The coefficient $m_{ij;xx'}^{\alpha\beta}$ encodes the linear response of the abundance of species i belonging to trophic block α at patch x due to a change in abundance of species j belonging to trophic block β at patch x'. We note that the only way the abundance of a species at one location is affected by the abundance at another location is through migration. So for $x \neq x'$, $m_{ij;xx'}^{\alpha\beta} \propto \delta_{ij}\delta_{\alpha\beta}$ (see Eqs. (S143) below).

The introduction of the patch coordinate x in this way gives rise to a higher level of block structure: the matrix is split into n^2 blocks corresponding to pairs of patches, and each of these blocks is split into four sub-blocks reflecting the trophic structure of the ecosystem.

More precisely, the interaction matrix has entries with the following statistics

$$\begin{aligned}
\overline{m_{ij;xx}^{\alpha\beta}} &= \frac{\mu_{\alpha\beta}}{N}, \quad \text{for} \quad i \neq j, \\
\overline{(m_{ij;xx}^{\alpha\beta})^2} &= \frac{\sigma^2}{N}, \\
\overline{m_{ij;xx}^{\alpha\beta} m_{ij;x'x'}^{\alpha\beta}} &= \frac{c_{x,x'}\sigma^2}{N}, \\
\overline{m_{ij;xx}^{\alpha\beta} m_{ji;xx}^{\beta\alpha}} &= \frac{\Gamma_{\alpha\beta}\sigma^2}{N}, \\
\overline{m_{ij;xx}^{\alpha\beta} m_{ji;x'x'}^{\beta\alpha}} &= \frac{c_{x,x'}\Gamma_{\alpha\beta}\sigma^2}{N}, \\
m_{ii;xx}^{\alpha\alpha} &= -d_\alpha - D_\alpha, \\
m_{ij;xx'}^{\alpha\beta} &= D_\alpha \phi_{x,x'} \delta_{ij}\delta_{\alpha\beta},
\end{aligned} \tag{S143}$$

where $x \neq x'$. We have introduced the spatial correlation kernel $c_{x,x'}$, which determines the degree to which interaction coefficients vary from patch to patch. For example, if $c_{x,x'} = 0$ for all $x \neq x'$, then the interactions are entirely uncorrelated across patches. If on the other hand $c_{x,x'} = 1$ for all x, x' then interactions are the same everywhere (no spatial heterogeneity). When we refer to the matrix $\mathbf{m}$ in this section, we assume the matrix to have this finer block structure and the above statistics.

By taking $c_{x,x'} = 1$ for all x, x', we recover the discrete-patch model discussed in Section S1 C, which in turn reduces to the model in the main text in the continuum limit. If instead we set $\Gamma_{\alpha\beta} = 0$ and $\mu_{\alpha\beta} = 0$ (i.e. remove statistical correlations and set the mean of the random matrix elements to zero) and do not have any notion of trophic structure (by setting $\gamma_u = 1$ for instance), we recover the model in GML [S1].

B. Saddle-point calculation for the bulk spectrum

Replica method and Hubbard-Stratononich transformation

In a similar spirit to the earlier calculation in Section S2, in order to find the bulk of the eigenvalue spectrum, we set the mean of the random matrix elements to zero $\mu_{\alpha\beta} = 0$. Any non-zero mean is later restored in order to find the outlier eigenvalues. We evaluate a potential $\Phi(\omega)$ obeying Eq. (S23) in the limit $N \to \infty$. We begin with the Gaussian representation of the determinant [c.f. Eq. (S29)]

$$\det\left[(\mathbb{1}_N\omega^\star - \mathbf{m}^T)(\mathbb{1}_N\omega - \mathbf{m})\right]^{-1} = \int \prod_{i,\alpha,x}\left(\frac{d^2 z_{i,x}^\alpha}{\pi}\right)$$
$$\times \exp\left[-\epsilon\sum_{i,\alpha,x}|z_{i,x}^\alpha|^2 - \sum_{i,j,k,\alpha,\beta,\gamma,x,x',x''} z_{i,x}^{\alpha\star}(\omega^\star\delta_{ik}\delta_{\alpha\gamma}\delta_{xx''} - (m^T)_{ik;xx''}^{\alpha\gamma})(\omega\delta_{kj}\delta_{\gamma\beta}\delta_{x'x''} - m_{kj;x''x'}^{\gamma\beta})z_{j,x'}^\beta\right]. \quad \text{(S144)}$$

Next, we perform a Hubbard–Stratonovich transformation [S31] in order to linearise the terms involving $m_{ij;x,x'}^{\alpha\beta}$ yielding

$$\det\left[(\mathbb{1}_N\omega^\star - \mathbf{m}^T)(\mathbb{1}_N\omega - \mathbf{m})\right]^{-1} = \int\prod_{i\alpha x}\left(\frac{d^2 z_{i,x}^\alpha d^2 y_{i,x}^\alpha}{2\pi^2}\right)\exp\left[-\sum_{i\alpha x} y_{i,x}^{\alpha\star}y_{i,x}^\alpha + \epsilon z_{i,x}^{\alpha\star}z_{i,x}^\alpha\right]$$
$$\times\exp\left[i\sum_{ij\alpha\beta xx'} z_{i,x}^{\alpha\star}\left(m_{ji;x'x}^{\beta\alpha} - \omega^\star\delta_{ij}\delta_{\alpha\beta}\delta_{xx'}\right)y_{jx'}^\beta\right]\exp\left[i\sum_{ij\alpha\beta xx'} y_{i,x}^{\alpha\star}\left(m_{ij;xx'}^{\alpha\beta} - \omega\delta_{xx'}\delta_{ij}\delta_{\alpha\beta}\right)z_{j,x'}^\beta\right]. \quad \text{(S145)}$$

Average over the random matrix and introduction of order parameters

Carrying out the average over the random matrix elements in Eq. (S145) then gives

$$\exp\left[-N\Phi(\omega)\right] = \int\prod_{i\alpha x}\left(\frac{d^2 z_{ix}^\alpha d^2 y_{ix}^\alpha}{2\pi^2}\right)\exp\left[-\sum_{i\alpha x} y_{i,x}^{\alpha\star}y_{ix}^\alpha + \epsilon z_{ix}^{\alpha\star}z_{ix}^\alpha\right]$$
$$\times\exp\left[-i\sum_{i\alpha x} z_{ix}^{\alpha\star}y_{ix}^\alpha(\omega^\star + d_\alpha + D_\alpha) + z_{ix}^\alpha y_{ix}^{\alpha\star}(\omega + d_\alpha + D_\alpha)\right]$$
$$\times\exp\left[i\sum_{i\alpha xx'} z_{ix}^{\alpha\star}y_{ix'}^\alpha D_\alpha\phi_{x,x'} + z_{ix}^\alpha y_{ix'}^{\alpha\star}D_\alpha\phi_{x,x'}\right]$$
$$\times\exp\left[-\frac{\sigma^2}{2N}\sum_{ij\alpha\beta x}(z_{ix}^{\alpha\star}y_{jx}^\beta + z_{ix}^\alpha y_{jx}^{\beta\star})^2 + \Gamma_{\alpha\beta}(z_{ix}^{\alpha\star}y_{jx}^\beta + z_{ix}^\alpha y_{jx}^{\beta\star})(z_{jx}^{\beta\star}y_{ix}^\alpha + z_{jx}^\beta y_{ix}^{\alpha\star})\right]$$
$$\times\exp\left[-\frac{\sigma^2}{2N}\sum_{ij\alpha\beta xx'} c_{x,x'}(z_{ix}^{\alpha\star}y_{jx}^\beta + z_{ix}^\alpha y_{jx}^{\beta\star})(z_{ix'}^{\alpha\star}y_{jx'}^\beta + z_{ix'}^\alpha y_{jx'}^{\beta\star})\right]$$

$$\times \exp\left[-\frac{\sigma^2}{2N}\sum_{ij\alpha\beta xx'} c_{x,x'}\Gamma_{\alpha\beta}(z_{ix}^{\alpha\star}y_{jx}^{\beta}+z_{ix}^{\alpha}y_{jx}^{\beta\star})(z_{jx'}^{\beta\star}y_{ix'}^{\alpha}+z_{jx'}^{\beta}y_{ix'}^{\alpha\star})\right]. \quad \text{(S146)}$$

To capture the non-trivial spatial variation in the interaction matrix elements between patches, we define the following order parameters (c.f. Eq. (S33))

$$\begin{aligned} u_{x,x'} &= \frac{1}{N}\sum_{i\alpha} z_{i,x}^{\alpha\star}z_{ix'}^{\alpha}, \quad v_{x,x'} = \frac{1}{N}\sum_{i\alpha} y_{i,x}^{\alpha\star}y_{ix'}^{\alpha}, \\ w_{\alpha;x,x'} &= \frac{1}{N_\alpha}\sum_{i} z_{ix}^{\alpha\star}y_{i,x'}^{\alpha}, \quad w_{\alpha;x,x'}^{\star} = \frac{1}{N_\alpha}\sum_{i} y_{i,x}^{\alpha\star}z_{ix'}^{\alpha}, \end{aligned} \quad \text{(S147)}$$

which we impose in the integral Eq. (S146) using Dirac delta functions in their complex exponential representation. We can thus rewrite Eq. (S146) as

$$\exp\left[-N\Phi(\omega)\right] = \int \mathcal{D}\left[\cdots\right]\exp\left[Nn(\Psi+\Theta+\Omega)\right], \quad \text{(S148)}$$

where $\mathcal{D}[\cdots]$ denotes integration over all of the order parameters and their conjugate ('hatted') variables, and where

$$\begin{aligned} \Psi =& i\sum_{x,x'}\left[\hat{u}_{x,x'}u_{x,x'}+\hat{v}_{x,x'}v_{x,x'}+\sum_{\alpha}\gamma_\alpha(\hat{w}_{\alpha;x,x'}w_{\alpha;x,x'}^{\star}+\hat{w}_{\alpha;x,x'}^{\star}w_{\alpha;x,x'})\right], \\ \Theta =& \sum_{x}\Bigg\{-\epsilon u_{x,x}-v_{x,x}-\sigma^2\sum_{x'}c_{x,x'}u_{x,x'}v_{x',x}+i\sum_{\alpha x'}\phi_{x,x'}D_\alpha\left[w_{\alpha;x,x'}+w_{\alpha;x,x'}^{\star}\right] \\ & -i\sum_{\alpha}\gamma_\alpha\left[w_{\alpha;x,x}(\omega^\star+d_\alpha+D_\alpha)+w_{\alpha;x,x}^{\star}(\omega+d_\alpha+D_\alpha)\right] \\ & -\frac{1}{2}\sigma^2\sum_{\alpha\beta}\gamma_\alpha\gamma_\beta\Gamma_{\alpha\beta}c_{x,x'}\left[w_{\alpha;x,x'}w_{\beta;x',x}+w_{\alpha;x',x}^{\star}w_{\beta;x,x'}^{\star}\right]\Bigg\}, \\ \Omega =& \sum_{\alpha}\gamma_\alpha\ln\Bigg[\int\prod_{x}\left(\frac{d^2z_x^\alpha d^2y_x^\alpha}{2\pi^2}\right)\exp\Bigg\{-i\sum_{xx'}(\hat{u}_{x,x'}z_x^{\alpha\star}z_{x'}^{\alpha}+\hat{v}_{x,x'}y_x^{\alpha\star}y_{x'}^{\alpha} \\ & +\hat{w}_{\alpha;x,x'}y_x^{\alpha\star}z_{x'}^{\alpha}+\hat{w}_{\alpha;x,x'}^{\star}z_x^{\alpha\star}y_{x'}^{\alpha})\Bigg\}\Bigg]. \end{aligned} \quad \text{(S149)}$$

To proceed we now assume that the correlation kernel $c_{x,x'}$ is only a function of the distance $|x-x'|$. The order parameters ($w_{\alpha;x,x'}$ and so on) then also only depend $|x-x'|$. We can exploit this translational invariance to simplify the calculation through the use of discrete Fourier transforms.

We introduce the Fourier transformed variables $\tilde{z}_{iq}$ and $\tilde{y}_{iq}$ through

$$\tilde{z}_{iq} = \sum_{x} e^{iqx}z_{ix}, \quad \tilde{y}_{iq} = \sum_{x} e^{iqx}y_{ix}. \quad \text{(S150)}$$

The inverse transform is given by

$$z_{ix} = \frac{1}{n}\sum_{q} e^{-iqx}\tilde{z}_{iq}, \quad y_{ix} = \frac{1}{n}\sum_{q} e^{-iqx}\tilde{y}_{iq}. \quad \text{(S151)}$$

We drop the tilde from further notation for the sake of compactness (all variables with subscript q or q' are Fourier transforms). One then obtains

$$\begin{aligned}
\Psi &= i\sum_q \left[\hat{u}_q u_{-q} + \hat{v}_q v_{-q} + \sum_\alpha \gamma_\alpha(\hat{w}_{\alpha;q}w^\star_{\alpha;-q} + \hat{w}^\star_{\alpha;q}w_{\alpha;-q})\right],\\
\Theta &= \sum_q \Bigg\{ -\epsilon u_q - v_q - \frac{\sigma^2}{n}\sum_{q'} c_{q-q'}u_q v_{q'} - i\sum_\alpha \gamma_\alpha\left[w_{\alpha;q}(\omega^\star + d_\alpha + D_\alpha) + w^\star_{\alpha;q}(\omega + d_\alpha + D_\alpha)\right]\\
&\quad + i\sum_\alpha \phi_q D_\alpha\left[w_{\alpha;q} + w^\star_{\alpha;q}\right] - \frac{\sigma^2}{2n}\sum_{\alpha\beta;q'}\gamma_\alpha\gamma_\beta\Gamma_{\alpha\beta}c_{q-q'}[w_{\alpha;q}w_{\beta;q'} + w^\star_{\alpha;q'}w^\star_{\beta;q}]\Bigg\},\\
\Omega &= -\sum_\alpha \gamma_\alpha \ln(\hat{w}_{\alpha;q}\hat{w}^\star_{\alpha;q} - \hat{u}_q\hat{v}_q) + \text{const.}
\end{aligned} \tag{S152}$$

Saddle-point integration and evaluation of order parameters

We now take the limit $N\to\infty$, and carry out the integral in Eq. (S148) using the saddle-point method. To do this, we extremise the expression $\Psi+\Theta+\Omega$ with respect to the order parameters and the conjugates. Extremising with respect to the conjugate variables $\hat{u}, \hat{v}, \hat{w}_\alpha$ and $\hat{w}^\star_\alpha$, we find

$$\begin{aligned}
iu_{-q} &= \sum_\alpha \frac{-\gamma_\alpha\hat{v}_q}{\hat{w}_{\alpha;q}\hat{w}^\star_{\alpha;q} - \hat{u}_q\hat{v}_q}, \quad iv_{-q} = \sum_\alpha \frac{-\gamma_\alpha\hat{u}_q}{\hat{w}_{\alpha;q}\hat{w}^\star_{\alpha;q} - \hat{u}_q\hat{v}_q},\\
i\gamma_\alpha w_{\alpha;-q} &= \frac{\gamma_\alpha\hat{w}_{\alpha;q}}{\hat{w}_{\alpha;q}\hat{w}^\star_{\alpha;q} - \hat{u}_q\hat{v}_q}, \quad i\gamma_\alpha w^\star_{\alpha;-q} = \frac{\gamma_\alpha\hat{w}^\star_{\alpha;q}}{\hat{w}_{\alpha;q}\hat{w}^\star_{\alpha;q} - \hat{u}_q\hat{v}_q}.
\end{aligned} \tag{S153}$$

Similar to Section S2 C, we simplify matters by defining $\hat{f}_{\alpha;q} = \frac{1}{(\hat{w}_{\alpha;q}\hat{w}^\star_{\alpha;q} - \hat{u}_q\hat{v}_q)}$, and we obtain an implicit equation for $\hat{f}_{\alpha;q}$ in terms of r_{-q} and $w_{\alpha;-q}w^\star_{\alpha;-q}$:

$$-w_{\alpha;-q}w^\star_{\alpha;-q} = \hat{f}_{\alpha;q} - \hat{f}^2_{\alpha;q}\frac{r_{-q}}{\left[\sum_\beta \gamma_\beta\hat{f}_{\beta;q}\right]^2}. \tag{S154}$$

We now extremise $\Psi+\Theta+\Omega$ in Eq. (S148) with respect to $u_q, v_q, w_{\alpha;q}$ and $w^\star_{\alpha;q}$. We find

$$\begin{aligned}
i\hat{u}_{-q} &= \epsilon + \frac{\sigma^2}{n}\sum_{q'} c_{q-q'}v_{q'}, \quad i\hat{v}_{-q} = 1 + \frac{\sigma^2}{n}\sum_{q'} c_{q-q'}u_{q'},\\
i\hat{w}_{\alpha;-q} &= i[\omega + d_\alpha + D_\alpha(1-\phi_q)] + \frac{\sigma^2}{n}\sum_\beta \Gamma_{\alpha\beta}\gamma_\beta\sum_{q'} c_{q-q'}w^\star_{\beta;q'},\\
i\hat{w}^\star_{\alpha;-q} &= i[\omega^\star + d_\alpha + D_\alpha(1-\phi_q)] + \frac{\sigma^2}{n}\sum_\beta \Gamma_{\alpha\beta}\gamma_\beta\sum_{q'} c_{q-q'}w_{\beta;q'}.
\end{aligned} \tag{S155}$$

Combining these with Eqs. (S153), one finally obtains

$$v_q = \left(\epsilon + \frac{\sigma^2}{n}\sum_{q'} c_{q-q'}v_{q'}\right)\sum_\alpha \gamma_\alpha\hat{f}_{\alpha;-q},$$

$$
\begin{aligned}
u_q &= \left(1 + \frac{\sigma^2}{n}\sum_{q'} c_{q-q'} u_{q'}\right)\sum_{\alpha}\gamma_\alpha \hat{f}_{\alpha;-q},\\
w_{\alpha;q} &= -\left[i(\omega + d_\alpha + D_\alpha(1-\phi_q)) + \frac{\sigma^2}{n}\sum_{\beta,q'}\Gamma_{\alpha\beta}\gamma_\beta c_{q-q'} w^\star_{\beta;q'}\right]\hat{f}_{\alpha;-q},\\
w^\star_{\alpha;q} &= -\left[i(\omega^\star + d_\alpha + D_\alpha(1-\phi_q)) + \frac{\sigma^2}{n}\sum_{\beta}\Gamma_{\alpha\beta}\gamma_\beta c_{q-q'} w_{\beta;q'}\right]\hat{f}_{\alpha;-q}.
\end{aligned}
\tag{S156}
$$

If we compare this result with the one in Eq. (S44), we observe two key differences: (1) The Fourier transform of the continuous Laplacian operator $-q^2$ is now replaced with its discrete counterpart $1-\phi_q$, (2) Because of the imperfect correlation between the interaction coefficients in different patches, the eigenvalue spectra for different Fourier modes q are no longer independent. This interdependence is encoded in the Fourier transform of the correlation kernel $c_{q-q'}$. For perfect correlation ($c_{x,x'} = 1$ for x and x') this reduces to $c_{q-q'} \propto \delta_{q,q'}$ and the eigenvalue spectra for different modes q once again decouple.

Although obtaining a general solution of Eqs. (S156) is complicated (while technically possible), the support of the eigenvalue spectrum can be found more easily in various special cases. We explore some of these in the following sections.

C. Outlier eigenvalues

Setup and general relations

In a similar fashion to Section S3, we now wish to find the outliers that arise when we perturb the random matrix $\mathbf{m}$ [whose statistics are given in Eq. (S143) with $\mu_{\alpha\beta} = 0$] by introducing a finite mean. That is, we are now interested in the matrix with elements

$$
m'^{\alpha\beta}_{ij;x,x'} = m^{\alpha\beta}_{ij;x,x'} + \frac{\mu_{\alpha\beta}}{N}\delta_{x,x'}, \tag{S157}
$$

and we define the matrix

$$
(\boldsymbol{\mu})^{\alpha\beta}_{ij;x,x'} = \mu_{\alpha\beta}\delta_{x,x'}, \tag{S158}
$$

which has elements independent of i and j.

Similar to the calculation in Section S3 any eigenvalue of the perturbed matrix outside the bulk of the unperturbed eigenvalue spectrum must satisfy

$$
\det\left[\mathbb{1}_{Nn} - \frac{1}{N}(\lambda \mathbb{1}_{Nn} - \mathbf{m})^{-1}\boldsymbol{\mu}\right] = 0. \tag{S159}
$$

Simplification to an effective 2n-species problem

We once again consider the resolvent matrix $\mathbf{G} = (\lambda \mathbb{1}_{Nn} - \mathbf{m})^{-1}$. Using reasoning along the lines of Section S3, one arrives at a similar expression to Eq. (S60)

$$\det \left[\mathbb{1}_{2n} - \boldsymbol{\mu}^{(2n)} \frac{1}{N} \sum_{ij} \mathbf{G}^{(ij)}(\lambda) \right] = 0, \tag{S160}$$

where here $\left(\mathbf{G}^{(ij)}\right)_{\alpha\beta;x,x'} = \left((\mathbb{1}_N \lambda - \mathbf{m})^{-1}\right)^{\alpha\beta}_{ij;x,x'}$ instead.

Final expression for outliers

Assuming the translational invariance of the averaged resolvent, one finds using Eq. (S61) in combination with the discrete Fourier transform (which can be represented as an orthonormal transformation)

$$\det \left[\mathbb{1}_{2n} - \boldsymbol{\mu}^{(2n)} \frac{1}{N} \sum_{ij} \mathbf{G}^{(ij)}(\lambda) \right] = \prod_q \det \left[\mathbb{1}_{2} - \boldsymbol{\mu}^{(2)} \frac{1}{N} \sum_{ij} \tilde{\mathbf{G}}^{(ij)}_q(\lambda) \right] = 0, \tag{S161}$$

Given that the resolvent matrix is approximately diagonal for large N (see Section S3), we only need to calculate the sum of diagonal elements $\frac{1}{N}\sum_i \tilde{G}^{\alpha\alpha}_{ii;q}$ in Eq. (S160). As in the calculation for the model used in the main text, we can identify these averaged resolvents with the order parameters $i\gamma_\alpha w^\star_{q;\alpha}$.

Our final equation for the outliers λ is

$$\prod_q \det \left[\mathbb{1}_2 - \boldsymbol{\mu}^{(2)} \boldsymbol{\gamma} \boldsymbol{\chi}_q(\lambda) \right] = 0, \tag{S162}$$

with

$$\boldsymbol{\gamma}(\lambda) = \begin{bmatrix} \gamma_u & 0 \\ 0 & \gamma_v \end{bmatrix}, \quad \boldsymbol{\chi}_q(\lambda) = \begin{bmatrix} \chi_{u;q} & 0 \\ 0 & \chi_{v;q} \end{bmatrix}. \tag{S163}$$

This can be written as

$$\prod_q \left\{ \left[\gamma_u \mu_{uu} - \frac{1}{\chi_{u;q}(\lambda)} \right] \left[\gamma_v \mu_{vv} - \frac{1}{\chi_{v;q}(\lambda)} \right] - \gamma_u \gamma_v \mu_{uv} \mu_{vu} \right\} = 0. \tag{S164}$$

Each value of q thus yields possible values for λ. We consider each value of q individually:

$$\left[\gamma_u \mu_{uu} - \frac{1}{\chi_{u;q}(\lambda)} \right] \left[\gamma_v \mu_{vv} - \frac{1}{\chi_{v;q}(\lambda)} \right] - \gamma_u \gamma_v \mu_{uv} \mu_{vu} = 0. \tag{S165}$$

We note that this takes the same form as in Eq. (S71). From Eqs. (S156), the quantities $\chi_{\alpha;q}(\lambda) = i w^\star_{q;\alpha}$ satisfy

$$-1 = -[\lambda + d_\alpha + D_\alpha(1 - \phi_q)]\chi_{\alpha;q} + \frac{\sigma^2}{n} \sum_{\beta;q'} \gamma_\beta \Gamma_{\alpha\beta} c_{q-q'} \chi_{\alpha;q} \chi_{\beta;q'}, \tag{S166}$$

and so the outliers for different values of q remain coupled through these equations.

As with the model used in the main text, not all solutions of this set of equations are valid. At the saddle point, the order parameter u_q must satisfy $u_q \geq 0$. This gives an additional criterion which is important for eliminating invalid solutions for λ.

S11. PREVIOUS RESULTS AS LIMITING CASES

A. Model in the main text

We now recover the model in the main text from Eqs. (S143) by taking the continuum limit in a similar way to Section S1 C, imposing that interactions do not vary in space, and making dispersal local. More precisely, we impose the following: (1) There are a large number patches, (2) these patches are spaced a small distance apart, (3) dispersal is only allowed between adjacent patches, (4) the interaction coefficients are the same in every patch (i.e. there is perfect correlation between the interaction coefficients in different patches).

The reduction can then be seen as follows. We set the correlation kernel to be constant in space $c_{x,x'} = 1$. The Fourier transform of the correlation kernel is then given by $\tilde{c}_{q-q'} = n\delta_{q,q'}$. Immediately, we see that the first two equations in Eq. (S156) decouple and we obtain

$$\begin{aligned} -w_{\alpha;-q}w^{\star}_{\alpha;-q} &= \hat{f}_{\alpha;q} - \hat{f}^2_{\alpha;q}\frac{r_{-q}}{\left[\sum_\beta \gamma_\beta \hat{f}_{\beta;q}\right]^2}, \\ v_q &= \left(\epsilon + \sigma^2 v_q\right)\sum_\alpha \gamma_\alpha \hat{f}_{\alpha;-q}, \\ u_q &= \left(1 + \sigma^2 u_q\right)\sum_\alpha \gamma_\alpha \hat{f}_{\alpha;-q}, \end{aligned} \tag{S167}$$

which are equivalent to the first two of Eqs. (S44) and the first of Eqs. (S47). We now choose the following local dispersal kernel

$$\phi(x,x') = \begin{cases} \frac{1}{2} & \text{if} \quad x = x' + \Delta x, \\ \frac{1}{2} & \text{if} \quad x = x' - \Delta x, \\ 0 & \text{otherwise}, \end{cases} \tag{S168}$$

where the separation between patches Δx is assumed small. We take the continuum limit in a similar way to Section S1 C. Noting that once again $\phi_q = \cos(\Delta x\, q)$, the last two equations in Eq. (S156) become

$$\begin{aligned} w_{\alpha;q} &= -\left[i(\omega + d_\alpha + D'_\alpha q^2) + \sigma^2 \sum_\beta \Gamma_{\alpha\beta}\gamma_\beta w^{\star}_{\beta;q'}\right]\hat{f}_{\alpha;-q}, \\ w^{\star}_{\alpha;q} &= -\left[i(\omega^{\star} + d_\alpha + D'_\alpha q^2) + \sigma^2 \sum_\beta \Gamma_{\alpha\beta}\gamma_\beta w_{\beta;q'}\right]\hat{f}_{\alpha;-q}, \end{aligned} \tag{S169}$$

and Eq. (S166) for the outliers becomes

$$-1 = -[\lambda + d_\alpha + D'_\alpha q^2]\chi_{\alpha;q} + \sigma^2\gamma_\beta\Gamma_{\alpha\beta}\chi_{\alpha;q}\chi_{\beta;q} \tag{S170}$$

where $D'_\alpha = D_\alpha(\Delta x)^2/2$. These take the same form as in Eqs. (S44) and Eqs. (S72) respectively. Hence, with appropriate scaling of the hopping constant, we recover the continuum model used in the main text.

B. Stabilising mechanism reported by Gravel, Massol and Leibold ('GML') [S1]

Specifications of the model

We now show how the results by Gravel, Massol and Leibold (GML) can be recovered. The model by GML does not contain trophic block-structure, so we need to remove the distinction between the groups of predator and prey species. This can be achieved by setting $\gamma_u = 1$ (implying $\gamma_v = 0$). The group labelled 'v' in our model is then no longer present. In the rest of this subsection (S11 B) for simplicity write D instead of D_u and d instead of d_u. We also replace Γ_u by Γ, μ_{uu} by μ and the remove the subscript u in the order parameters.

Further, the interaction matrix entries for a single patch in GML [S1] are each drawn independently – correlations between random matrix entries are not considered ($\Gamma = 0$). Finally, the mean of the interaction matrix elements is also zero ($\mu = 0$). As a result, there are no outlier eigenvalues.

With these simplifications, the model by GML can be obtained from Eqs. (S143) by choosing the following dispersal kernel and correlations between interactions in different patches,

$$
\begin{aligned}
c_{x,x'} &= \delta_{x,x'} + (1 - \delta_{x,x'})\rho, \\
\phi_{x,x'} &= (1 - \delta_{x,x'})\frac{1}{n-1},
\end{aligned}
\tag{S171}
$$

The degree to which there is spatial heterogeneity (or conversely, how much different patches are correlated) is quantified by the parameter ρ. This may vary between 0 (patches are completely independent) and 1 (where all patches have identical interaction coefficients). With the aforementioned restrictions in mind, we now show that the results for the eigenvalue spectra derived in [S1] also follow from Eqs. (S156). From these, we then also demonstrate that stabilisation can be result from the combination of dispersal and spatial heterogeneity.

Eigenvalue spectra

The Fourier transforms of the kernels in Eqs. (S171) are

$$
\begin{aligned}
c_q &= \left[1 + \rho(n\delta_{q,0} - 1)\right], \\
\phi_q &= \frac{(n\delta_{q,0} - 1)}{n-1}.
\end{aligned}
\tag{S172}
$$

We observe that there are now only two relevant cases for the value of q. The first is $q = 0$ and the second encompasses all values $q \neq 0$. In particular we have $v_q = v_{q'}$ (for q and q' both non-zero) and similarly for the other order parameters.

In the limit $\epsilon \to 0^+$ [c.f. Eq. (S155)], the first of Eqs. (S156) becomes

$$
\begin{aligned}
v_0 &= \frac{\sigma^2}{n}\left[[1 + \rho(n-1)]v_0 + (n-1)(1-\rho)v_q\right]\hat{f}_0, \\
v_q &= \frac{\sigma^2}{n}\left[(n - 1 + \rho)v_q + (1-\rho)v_0\right]\hat{f}_{-q},
\end{aligned}
\tag{S173}
$$

where again $q \neq 0$. There are two solutions to these equations. One is the trivial solution $v_0 = v_q = 0$. The other is found by recognising Eqs. (S173) as an eigenvector problem. Thus for non-trivial

v_0 and v_q we must have

$$\begin{aligned}&\hat{f}_0\left[1+\rho(n-1)\right]+\hat{f}_{-q}\left[\rho+n-1\right]\\&+\frac{\sigma^2}{n}\hat{f}_0\hat{f}_{-q}\left[(1-\rho)^2(n-1)-(1+\rho(n-1))(\rho+n-1)\right]=\frac{n}{\sigma^2}.\end{aligned}\tag{S174}$$

The following conditions must be satisfied in order for the order parameters to have the correct properties (i.e. sign and complex conjugate relations)

$$\begin{aligned}&-w_0^\star w_0>0,\quad -w_0^\star w_0\in\mathbb{R},\quad -w_q^\star w_q>0,\quad -w_q^\star w_q\in\mathbb{R},\\&1-\frac{\sigma^2}{n}[1+\rho(n-1)]\hat{f}_0>0,\quad 1-\frac{\sigma^2}{n}[\rho+n-1]\hat{f}_{-q}>0.\end{aligned}\tag{S175}$$

When $v_0=v_q=0$, we have $\hat{f}_0=-w_0^\star w_0$ and $\hat{f}_{-q}=-w_{-q}^\star w_{-q}$ [by definition – c.f. Eq. (S154)]. Therefore, the last two of Eqs. (S156) yield analytic expressions for $iw_0^\star$ and $iw_q^\star$ in ω, meaning that there is zero eigenvalue density where this trivial solution is the only one possible. The boundary of the region of the complex plane where there is non-zero eigenvalue density is given by the values of ω for which Eq. (S174) and $\hat{f}_0=-w_0^\star w_0$ and $\hat{f}_{-q}=-w_{-q}^\star w_{-q}$ are simultaneously true. Together with Eqs. (S156), we have

$$\hat{f}_0=\frac{1}{|\omega+d|^2},\quad \hat{f}_{-q}=\frac{1}{|\omega+d+Dn/(n-1)|^2}.\tag{S176}$$

Combining this with Eq. (S174) yields for the boundary of the eigenvalue support

$$\begin{aligned}&\frac{n}{\sigma^2}|\omega+d+Dn/(n-1)|^2|\omega+d|^2-\left[1+\rho(n-1)\right]|\omega+d+Dn/(n-1)|^2-\left[\rho+n-1\right]|\omega+d|^2\\&=\frac{\sigma^2}{n}\left[(1-\rho)^2(n-1)-(1+\rho(n-1))(\rho+n-1)\right].\end{aligned}\tag{S177}$$

In the case $\rho=0$, this simplifies further and we obtain

$$\frac{1}{|\omega+d|^2}+\frac{(n-1)}{|\omega+d+Dn/(n-1)|^2}=\frac{n}{\sigma^2}.\tag{S178}$$

Simplification is also possible in the case $\rho=1$. The support of the eigenvalue spectrum here is given by the union of the area within two circles:

$$|\omega+d|^2=\frac{\sigma^2}{n},\quad |\omega+d+Dn/(n-1)|^2=\frac{\sigma^2}{n}.\tag{S179}$$

The expressions in Eqs. (S178) and (S179) were reported in Ref. [S1] [see the discussion in Section S1 of the Supplementary Material of [S1] and in particular Eq. (1.6)] but were arrived at using a different method.

The result for general $0<\rho<1$ in Eq. (S177) was not explicitly written down in [S1]. Using our approach, we are able to extend the results to matrices with block structure and more complicated statistics ($\Gamma\neq 0$ and $\mu\neq 0$) and local dispersal (as opposed to all-to-all), as is discussed in more detail in Section S12.

Stabilising mechanism

Now that we have obtained analytical results for the eigenvalue spectra, we highlight the stabilising effect of the combination of spatial heterogeneity and dispersal in the model by GML. First we examine the case $D = 0$. Here, Eq. (S177) reduces to

$$\left\{|\omega + d|^2 - \frac{\sigma^2}{n}\left[1 + \rho(n-1)\right]\right\}\left\{|\omega + d|^2 - \frac{\sigma^2}{n}\left[\rho + (n-1)\right]\right\} = \frac{\sigma^4}{n^2}(1-\rho)^2(n-1). \tag{S180}$$

For reasonably large n therefore, the boundary of the spectrum for $D = 0$ is well-approximated by $|\omega + d|^2 = \sigma^2$ (this is verified in Fig. S8(a)). This is the same result one would obtain without spatial heterogeneity. This means that spatial heterogeneity only has an appreciable effect on stability once dispersal is introduced.

In Fig. S8 we demonstrate how the combination of dispersal and spatial heterogeneity can be stabilising. Panel (a) shows an example of a spectrum for an unstable equilibrium of a system without dispersal. The data shown is for $\rho = 1$, but as one would expect from the above reasoning, we find that the spectrum does not depend substantially on ρ for $D = 0$. When we introduce dispersal but no spatial heterogeneity [Fig. S8(b)], we find no stabilising effect. This would be expected from our discussion in Section S5 C: dispersal alone cannot stabilise a system. However, when we introduce heterogeneity along with dispersal, the system is stabilised. This can be seen by comparing panel (c) $[D = 0.9, \rho = 0.5]$ to panel (a) $[D = 0, \rho = 1]$. Fig. S8(d) $[D = 0.9, \rho = 0]$ shows that the stabilising effect is stronger as the interactions become more heterogeneous in space.

Conversely, in Fig. S9, we illustrate the effect of increasing the rate of dispersal D. Panel (a) once again shows an eigenvalue spectrum for $D = 0$, this time with maximum heterogeneity ($\rho = 0$). As we increase the rate of dispersal from zero (panels (b) $D = 0.9$, (c) $D = 2.5$, (d) $D = 3.5$), the larger part of the spectrum (associated with the $q \neq 0$ Fourier modes) moves leftwards. The part of the spectrum associated with the $q = 0$ Fourier mode (the smaller disk in panels (c) and (d)) remains unmoved as the magnitude of the dispersal is increased. If this smaller disk were large enough that some of the eigenvalues comprising it crossed the imaginary axis, then dispersal would not be able to stabilise the equilibrium, regardless of the value of D.

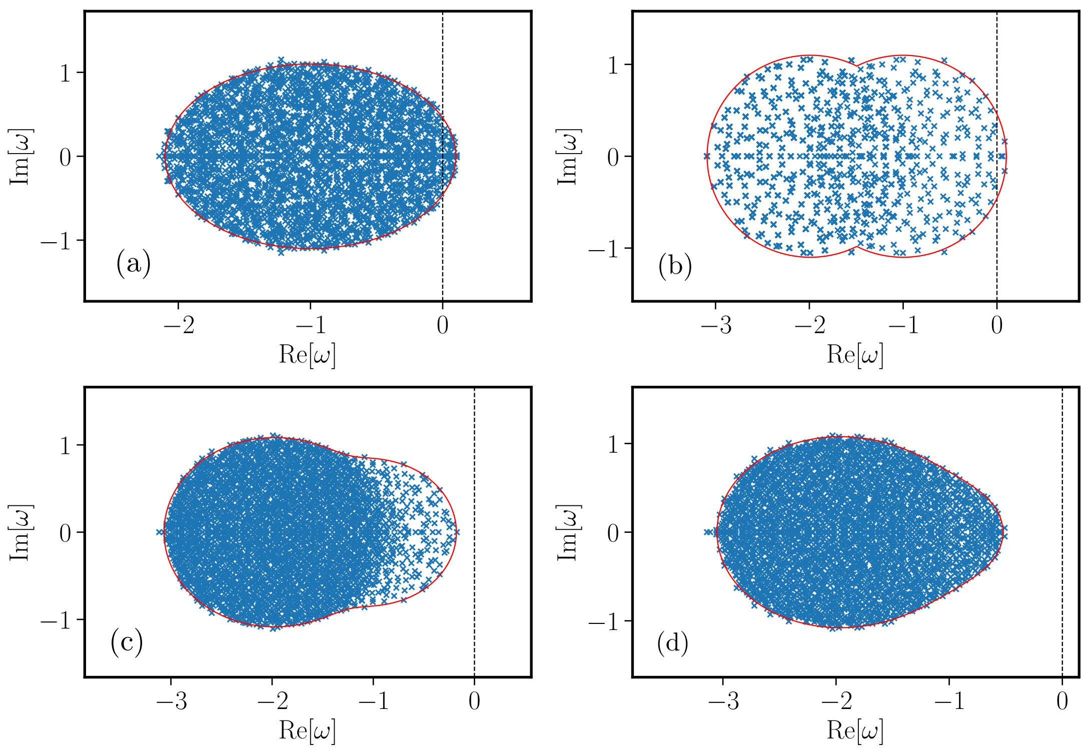

FIG. S8. **Dispersal can stabilise an equilibrium when interactions are heterogeneous in space.** The figure shows eigenvalue spectra for instances of the model in [S1]. Spectra of computer-generated random interaction matrices are shown as blue crosses. Red lines are the theoretical predictions for the boundary of the support from Eq. (S177). In all panels (after rescaling, see Sec. S4), $d = 1$, $n = 11$, $N = 300$, $C = 1$, $c = NC\sigma^2 = 1.21$. In panel (a) there is no dispersal ($D = 0$) and the equilibrium is unstable (data is for $\rho = 1$). In panel (b) there is now dispersal ($D = 0.9$) but no spatial heterogeneity ($\rho = 1$). The equilibrium is still unstable. In panel (c) spatial heterogeneity and dispersal has been introduced ($\rho = 0.5,\ D = 0.9$); the disk in the spectrum corresponding to the $q = 0$ mode has become smaller, and the equilibrium stabilised. In panel (d) the equilibrium is stabilised further by greater heterogeneity ($\rho = 0$ – complete independence between patches, $D = 0.9$).

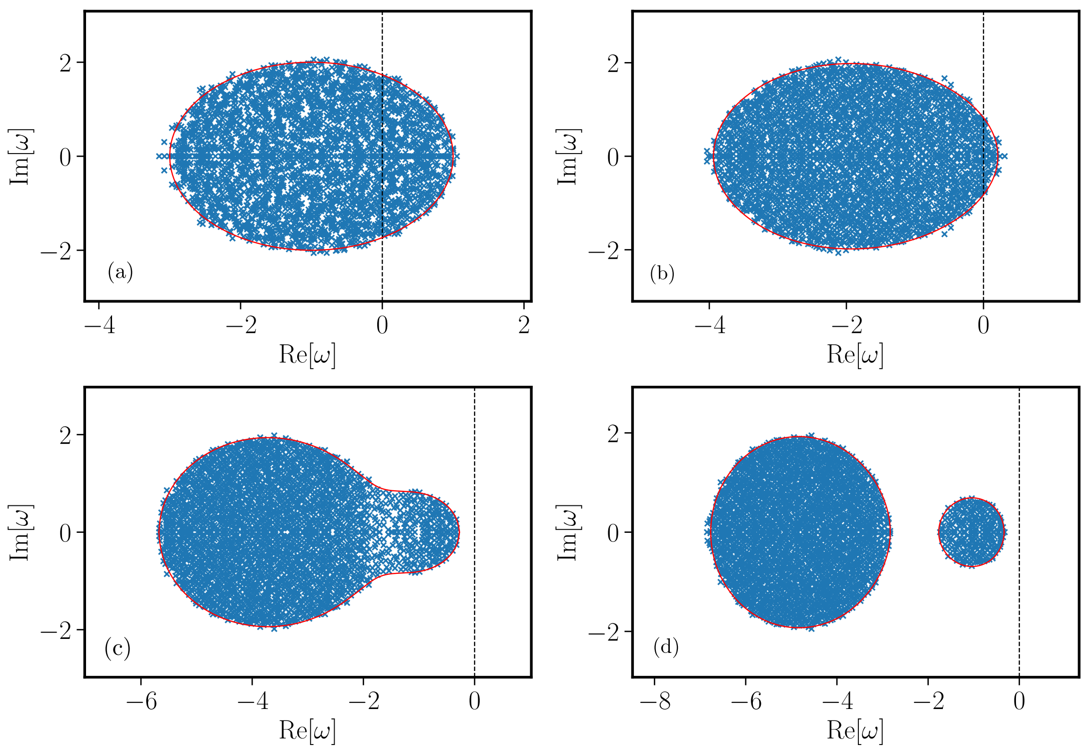

FIG. S9. **Dispersal must be sufficiently strong to produce stabilisation.** The figure shows eigenvalue spectra for instances of the model in [S1]. Spectra of computer-generated random interaction matrices are shown as blue crosses. Red lines are the theoretical predictions for the boundary of the support from Eq. (S177). In all panels (after rescaling, see Sec. S4), $\rho = 0$, $d = 1$, $n = 11$, $N = 300$, $C = 1$, $c = NC\sigma^2 = 4$. In panel (a) there is no dispersal ($D = 0$) and the equilibrium is unstable. In panel (b) there is now dispersal ($D = 0.9$). The equilibrium is still unstable. In panel (c) dispersal is increased ($D = 2.5$), the larger bulk region associated with the $q \neq 0$ modes is shifted leftwards and the equilibrium stabilised. In panel (d), the larger bulk region for $q \neq 0$ is shifted further leftwards, while the region associated with $q = 0$ remains in the same location (here $D = 3.5$).

S12. ADDITIONS TO THE MODEL BY GML

We now introduce some additional features to the model of GML [S1] using our general results derived in Section S10. Specifically, we show in Section S12 A that stabilisation not only occurs for all-to-all dispersal between patches, but also when dispersal is local (i.e., between nearest-neighbour patches). In Section S12 B we then discuss the introduction of local interspecies correlations (setting $\Gamma \neq 0$). Section S12 C then contains a discussion of outlier eigenvalues. These are responsible for the Turing instability, which is the focus of our work. We show that, unlike bulk eigenvalues, they are not necessarily subject to the stabilisation mechanism.

For this section, as with subsection S11 B, we take $\gamma_v = 0$ and suppress the subscript u for the sake of brevity.

A. The stabilising mechanism is preserved when dispersal is local

Eigenvalue spectra for local dispersal

We now examine the impact that changing the dispersal kernel from all-to-all to local has on the results presented in Fig. S8 and in Eq. (S177). We now use the local dispersal kernel (see also Eq. (S168))

$$\phi_{x,x'} = \begin{cases} \frac{1}{2} & \text{if} \quad x = x' - 1 \\ \frac{1}{2} & \text{if} \quad x = x' + 1 \\ 0 & \text{otherwise} \end{cases} . \tag{S181}$$

This has the Fourier transform $\phi_q = \cos(q)$ (as in Section S11 A, but setting $\Delta x = 1$). We keep the same form for $c_{x,x'}$ as in Eq. (S171). Eqs. (S156) then reduce to

$$\begin{aligned} v_q &= \frac{1}{n}\left[\epsilon n + \sigma^2(1-\rho)\sum_{q'} v_{q'} + n\rho\sigma^2 v_q\right]\hat{f}_{-q}, \\ u_q &= \frac{1}{n}\left[n + \sigma^2(1-\rho)\sum_{q'} u_{q'} + n\rho\sigma^2 u_q\right]\hat{f}_{-q}, \\ w_q &= -i\left[\omega + d + D[1-\cos(q)]\right]\hat{f}_{-q}, \\ w_q^\star &= -i\left[\omega^\star + d + D[1-\cos(q)]\right]\hat{f}_{-q}, \end{aligned} \tag{S182}$$

bearing in mind the restrictions mentioned at the start of Section S11 B. Due to the larger number of contributing Fourier modes, we cannot proceed as easily as in the case of all-to-all dispersal. However, we can still approximate the boundary of the eigenvalue support quite effectively. As we will see below, the equations approximately decouple for different values of q when the diffusion coefficient is sufficiently large.
First we deduce from the last two of Eqs. (S182) that

$$\hat{f}_{-q} = \frac{1}{|\omega + d + D[1-\cos(q)]|^2} \tag{S183}$$

on the boundary of the eigenvalue support, where $\hat{f}_{-q} = -w_q^\star w_q$. This means that for large D, $\hat{f}_{-q}$ is small unless $\omega \approx -d - D[1-\cos(q)]$. This indicates that the eigenvalue support is divided into clumps, one for each value of $1-\cos(q)$. When $\hat{f}_{-q}$ is small, so is v_q. Consequently, we can proceed based on the approximation that only one value of v_q is non-zero in any one region of the complex plane.

We wish to find the values of ω at which Eqs. (S182) first permit a non-zero solution. The solution only depends on the value of $\cos(q)$ and not on q directly. This means that $v_q = v_{q'}$ when $\cos(q) = \cos(q')$. We first focus on the case in which q does not take values $q = 0$ or $q = \pi$. For these values of q, the non-trivial solution of the first of Eqs. (S182) is given by$\hat{f}_{-q} = \frac{n}{\sigma^2}\left[2(1-\rho) + n\rho\right]^{-1}$. The boundaries of the clumps of eigenvalues are therefore given by the solution of $\hat{f}_{-q} = \frac{n}{\sigma^2}\left[2(1-\rho) + n\rho\right]^{-1} = -w_q^\star w_q$, with $w_q^\star$ and w_q being given by the last two

of Eqs. (S182). This yields

$$|\omega + d + D[1 - \cos(q)]|^2 = \frac{\sigma^2}{n}\,[2 + \rho(n-2)]\,. \tag{S184}$$

When $q = 0$ or $q = \pi$, instead the the non-trivial solution of the first of Eqs. (S182) is given by $\hat{f}_{-q} = \frac{n}{\sigma^2}\,[(1-\rho) + n\rho]^{-1}$. Hence, the boundaries of the clumps of eigenvalues corresponding to these values of q are given by

$$|\omega + d + D(1 - \cos(q))|^2 = \frac{\sigma^2}{n}\,[1 + \rho(n-1)]\,. \tag{S185}$$

We recall that we have assumed D to be large so that the approximation after Eq. (S183) is valid.

For $D = 0$, we see that the boundary of the eigenvalue spectrum becomes independent of the dispersal kernel. For sufficiently large n we can therefore proceed as in as in Section S11 B, and approximate the equation characterising the boundary as $|\omega + d|^2 = \sigma^2$.

We therefore see that the stabilising effect of the combination of dispersal and heterogeneity remains when the dispersal is local: For $D = 0$, the support of the spectrum is approximately circular, with radius approximately σ and centre $-d$. An example is shown in Fig. S10, where we choose $\rho = 0.5$. We note that the aspect ratio for the image in panel (a) was chosen in-line with that of panel (b), the scaling on the axes in (a) stretches the circular disk in horizontal direction. When we introduce dispersal ($D > 0$), modes with non-zero values of q become relevant. The circular patch of eigenvalues closest to the imaginary axis now has a smaller radius of approximately $\sigma\sqrt{[1 + \rho(n-1)]/n} < \sigma$ and the spectrum is now entirely in the negative half-plane.

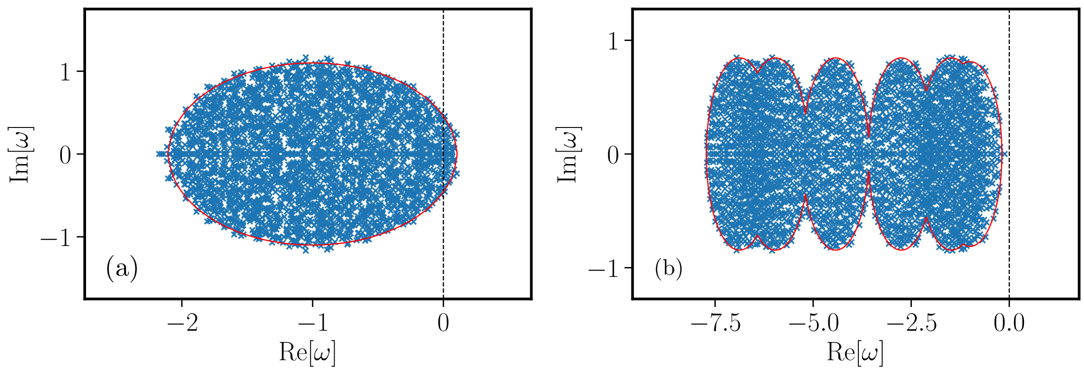

FIG. S10. **Stabilising mechanism is preserved when dispersal is local.** The system parameters here are the same as in Fig. S8, with the exception that dispersal is now local. In particular we have $\rho = 0.5$. Panel (a) shows the case $D = 0$ so that the spectrum is the same as Fig. S8(a). The diffusion constant in panel is $D = 3$. The example also demonstrates the approximation of the bulk regions as circles [see text after Eq. (S183)] is reasonably accurate even when D is only moderately large. The aspect ratio and size of the image for panel (a) was chosen in-line with that of panel (b). As a consequence the scaling on the axes in (a) stretches the circular disk in horizontal direction.

Stability criteria for all-to-all and nearest-neighbour dispersal

We first consider all-to-all dispersal. The boundary of the support of the eigenvalue spectrum in this case is given by Eq. (S177). When D is large, this is well approximated by two circles,

$$\begin{aligned} |\omega + d|^2 &= \frac{\sigma^2}{n}\left[1+\rho(n-1)\right], \\ |\omega + d + Dn/(n-1)|^2 &= \frac{\sigma^2}{n}\left[n-1+\rho\right]. \end{aligned} \tag{S186}$$

For large diffusion constants, the first of these disks is closer to the imaginary axis and so the criterion for stability is

$$\sigma^2 < \frac{d^2 n}{1+\rho(n-1)}. \tag{S187}$$

Keeping in mind that we have re-scaled the variance of interactions (see Section S4), we need to carry out the replacement $\sigma^2 \to \sigma^2 N$. In the limit of large N (which we always assume), Eq. (S187) is then the same as the stability criterion in Eq. (3) of [S1].

In the case of nearest-neighbour dispersal between patches, since the clump of eigenvalues corresponding to $q = 0$ is the same as in the all-to-all case [compare Eqs. (S186) and (S185)], we obtain the same criterion for stability from Eqs. (S185). As a consequence, the stability criteria for the model in [S1] remain unchanged if all-to-all dispersal between patches is replaced by local dispersal. The stabilising mechanism for bulk eigenvalues due to spatial heterogeneity and dispersal therefore remains.

B. Inclusion of correlations: $\Gamma \neq 0$

The work in Ref. [S1] assumed that the interaction coefficients between species within a patch could all be drawn independently, in a similar style to May's original model [S32]. Our analytical approach allows one to include correlations. We deduce the boundary of the eigenvalue support in this case and show that $\Gamma < 0$ can further stabilise the community.

We again use the dispersal and correlation kernels in Eq. (S171). From the first relation in Eq. (S156), we once again obtain [c.f. Eq. (S174)]

$$\hat{f}_0\left[1+\rho(n-1)\right] + \hat{f}_{-q}\left[\rho+n-1\right] + \frac{\sigma^2}{n}\hat{f}_0\hat{f}_{-q}\left[(1-\rho)^2(n-1) - (1+\rho(n-1))(\rho+n-1)\right] = \frac{n}{\sigma^2}. \tag{S188}$$

Now, because of the new asymmetry $\Gamma \neq 0$, the last two of Eqs. (S156) for the order parameters $w_q^\star$ and w_q no longer decouple for different values of q. We instead obtain

$$\begin{aligned} w_0 &= -\left\{ i[\omega + d] + \frac{\Gamma\sigma^2}{n}\left[[1+\rho(n-1)]w_0^\star + (1-\rho)(n-1)w_q^\star\right] \right\}\hat{f}_0, \\ w_0^\star &= -\left\{ i[\omega^\star + d] + \frac{\Gamma\sigma^2}{n}\left[[1+\rho(n-1)]w_0 + (1-\rho)(n-1)w_q\right] \right\}\hat{f}_0, \\ w_q &= -\left\{ i[\omega + d + Dn/(n-1)] + \frac{\Gamma\sigma^2}{n}\left[(\rho+n-1)w_q^\star + (1-\rho)w_0^\star\right] \right\}\hat{f}_{-q}, \end{aligned}$$

$$w_q^\star = -\left\{ i[\omega^\star + d + Dn/(n-1)] + \frac{\Gamma\sigma^2}{n}\left[(\rho+n-1)w_q + (1-\rho)w_0\right] \right\} \hat{f}_{-q}, \tag{S189}$$

For the boundary of the support of the eigenvalue spectrum, these must be solved simultaneously with $\hat{f}_0 = -w_0 w_0^\star$ and $\hat{f}_{-q} = -w_q w_q^\star$ and Eq. (S188) subject to the following conditions

$$\begin{aligned} -w_0^\star w_0 > 0, \quad -w_0^\star w_0 \in \mathbb{R}, \quad -w_q^\star w_q > 0, \quad -w_q^\star w_q \in \mathbb{R}, \\ 1 - \frac{\sigma^2}{n}\hat{f}_0[1+\rho(n-1)] > 0, \quad 1 - \frac{\sigma^2}{n}\hat{f}_{-q}[\rho+n-1] > 0. \end{aligned} \tag{S190}$$

Although these equations are difficult to handle analytically, they can be solved numerically. One finds that negative correlations $\Gamma < 0$ between the interaction coefficients $m_{ij;xx}$ and $m_{ji;xx}$ of species i and j within the same patch x further stabilises an equilibrium to an even greater extent than dispersal and heterogeneity on their own, an example is shown in Fig. S11. Such negative correlation between pairs of interaction coefficients in matrices without block structure is tantamount to predation.

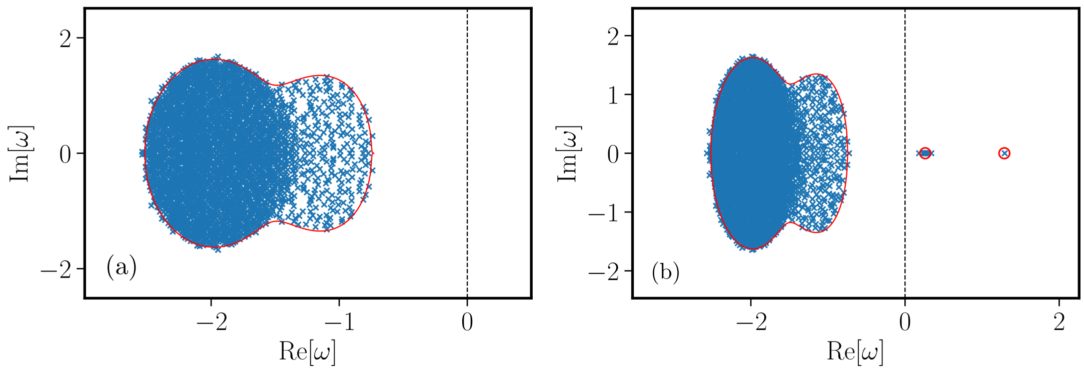

FIG. S11. **Verification of theory when interactions are locally correlated, but heterogeneous in space.** Panel (a): The setup and parameters are identical to those in Fig. S8(c) with the sole adjustment $\Gamma = -0.5$. The boundary of the bulk region is predicted by Eqs. (S189) and the outlier eigenvalues are found using Eq. (S191). The equilibrium is more stable than that in Fig. S8(c), confirming that anti-correlation in the interaction matrix acts to stabilise. In panel (b) the interaction matrix elements have a finite mean $NC\mu = 2.5$ (after rescaling, see Sec. S4) This produces two sets of outlier eigenvalues [one for $q = 0$ and one for $q \neq 0$ in equations Eq. (S164)]. As before blue crosses are from numerically diagonalising computer-generated random matrices, red lines and circles represent the analytical predictions for bulk and outlier eigenvalues respectively.

C. Outlier eigenvalues are not necessarily subject to the same stabilising effect as the bulk

As a precursor to the full inclusion of trophic structure in Section S14, we demonstrate the effect including a non-zero mean ($\mu \neq 0$) in the model of GML [S1]. In the original work in Ref. [S1], the interaction matrix elements were assumed to be drawn from a Gaussian distribution with zero mean ($\mu = 0$). As we have previously discussed, the inclusion of a non-zero mean can have a profound effect on stability. We can examine this effect using Eq. (S166), which becomes

$$-1 = -[\lambda + d]\chi_0 + \frac{\sigma^2\Gamma}{n}\chi_0\left\{[1+\rho(n-1)]\chi_0 + (1-\rho)(n-1)\chi_q\right\},$$

$$-1 = -[\lambda + d + Dn/(n-1)]\chi_q + \frac{\sigma^2\Gamma}{n}\chi_q \left\{[\rho + (n-1)]\chi_q + (1-\rho)\chi_0\right\}. \tag{S191}$$

These are solved simultaneously [c.f. Eq. (S165)] first with

$$\chi_0(\lambda) = \frac{1}{\mu}, \tag{S192}$$

to yield one set of outlier eigenvalues and then with

$$\chi_q(\lambda) = \frac{1}{\mu}, \tag{S193}$$

to yield another set. Again, one must impose $u_0 > 0$ and $u_q > 0$ [c.f. Eqs. (S156)] to eliminate invalid solutions. These expressions for the outliers are verified in Fig. S11.

When $\Gamma = 0$ (as was the case in the model originally presented in Ref. [S1]), we obtain the remarkably simple result for the outliers

$$\lambda_q = \mu - [d + D(1-\phi_q)]. \tag{S194}$$

Crucially, when $\Gamma = 0$, the outlier eigenvalues are independent of ρ (and therefore heterogeneity). This example demonstrates that the outlier eigenvalues are not necessarily subject to the same stabilising effects as the bulk distribution. This will prove important when we discuss the interplay between the stabilising effect of spatial heterogeneity and our destabilising mechanism in Section S14.

S13. DISPERSAL-INDUCED INSTABILITY PERSISTS ON A DISCRETE-PATCH LANDSCAPE WITH ALL-TO-ALL DISPERSAL

In Section S12 A we showed that the stabilising mechanism reported by [S1] in a model without trophic structure persists when dispersal is local. Here, we now demonstrate a complementary statement: dispersal-induced instability in the model with trophic structure continues to be possible in case of all-to-all dispersal between a set of patches.

Using the dispersal and correlation kernels in Eq. (S171) with $\rho = 1$, the equations for the order parameters decouple for different values of q, and we obtain

$$\begin{aligned}
-w_{\alpha;-q}w^\star_{\alpha;-q} &= \hat{f}_{\alpha;q} - \hat{f}^2_{\alpha;q}\frac{r_{-q}}{\left[\sum_\beta \gamma_\beta \hat{f}_{\beta;q}\right]^2},\\
v_q &= \left(\epsilon + \sigma^2 v_q\right)\sum_\alpha \gamma_\alpha \hat{f}_{\alpha;-q},\\
u_q &= \left(1 + \sigma^2 u_q\right)\sum_\alpha \gamma_\alpha \hat{f}_{\alpha;-q},\\
w_{\alpha;q} &= -\left[i\{\omega + d_\alpha + D_\alpha[1 - (n\delta_{q,0} - 1)/(n-1)]\} + \sigma^2 \sum_\beta \Gamma_{\alpha\beta}\gamma_\beta w^\star_{\beta;q}\right]\hat{f}_{\alpha;-q},\\
w^\star_{\alpha;q} &= -\left[i\{\omega^\star + d_\alpha + D_\alpha[1 - (n\delta_{q,0} - 1)/(n-1)]\} + \sigma^2 \sum_\beta \Gamma_{\alpha\beta}\gamma_\beta w_{\beta;q}\right]\hat{f}_{\alpha;-q},
\end{aligned} \tag{S195}$$

These equations are almost identical to those derived for the model used in the main text, the only difference being the dispersal kernel. They can be obtained from Eqs. (S44) with the substitution $q^2 \to 1 - \phi_q$.

We expect to see two contributions to the eigenvalue spectra, one for $q = 0$ and one for $q \neq 0$. The $q = 0$ contribution is the same as that which is found from Eqs. (S44) since, due to total probability, $1 - \phi_0 = 0$ (a statement which is independent of the hopping kernel). When $q \neq 0$, we have for the all-to-all hopping kernel that $1 - \phi_q = \frac{n}{n-1}$ [c.f. Eqs. (S172)]. The second contribution to the spectrum can therefore be obtained by setting $q = \sqrt{\frac{n}{n-1}}$ in the model in Section S1.

We can also calculate the outlier eigenvalues, whose locations are given by Eqs. (S165) and (S166). Again, the equations decouple for different values of q when $\rho = 1$, and we obtain a result similar to those in Eqs. (S71) and (S72), with the modification $q^2 \to 1 - \phi_q$,

$$\left[\gamma_u \mu_{uu} - \frac{1}{\chi_{u;q}(\lambda)}\right]\left[\gamma_v \mu_{vv} - \frac{1}{\chi_{v;q}(\lambda)}\right] - \gamma_u \gamma_v \mu_{uv} \mu_{vu} = 0,$$
$$-1 = -[\lambda + d_\alpha + D_\alpha(1 - \phi_q)]\chi_{\alpha;q} + \sigma^2 \sum_{\beta;q'} \gamma_\beta \Gamma_{\alpha\beta} \chi_{\alpha;q} \chi_{\beta;q}. \tag{S196}$$

These are to be solved subject to

$$\sum_\alpha \gamma_\alpha |\chi_{\alpha;q}|^2 < \sigma^2. \tag{S197}$$

We reach the conclusion that the system with all-to-all dispersal is equivalent to a system with continuous-space local diffusion restricted to Fourier modes $q = 0$ and $q = \sqrt{\frac{n}{n-1}}$. Therefore, if the mode with $q = 0$ is stable and the mode with $q = \sqrt{\frac{n}{n-1}}$ unstable in the main-text model (with local, diffusive dispersal), then we also observe a dispersal-induced instability in the model with discrete patches and all-to-all dispersal. An explicit example is given in Fig. S12.

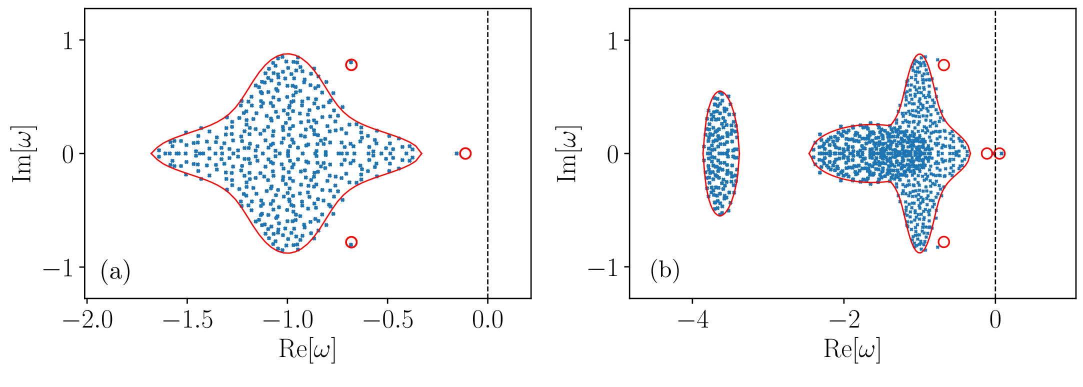

FIG. S12. **Dispersal-induced instability persists in discrete-patch landscape with all-to-all dispersal.** Panel (a) shows the spectrum when there is no dispersal ($D_u = D_v = 0$). In panel (b) $D_u = 0.5$, $D_v = 2.5$. The spectrum in (a) has the same support and outliers as that in Fig. 2(a) in the main text. The spectrum in (b) demonstrates that dispersal-induced instability still occurs when dispersal is all-to-all between a discrete set of patches. As before, predictions from the analytical theory are shown as red lines and circles, and numerical results for computer-generated random matrices as blue crosses. Parameters are (after rescaling, see Sec. S4) $NC\mu_{uu} = 3$, $NC\mu_{uv} = -3$, $NC\mu_{vu} = 3$, $NC\mu_{vv} = -1.5$, $c = NC\sigma^2 = 0.325$, $n = 11$, $\rho = 1$, $\Gamma_{uv} = -0.9$, $\Gamma_{uu} = 0.5$, $\Gamma_{vv} = -0.5$, $\gamma_u = 2/3$, $\gamma_v = 1/3$, $C = 1$ and $N = 450$.

S14. COMBINING TROPHIC STRUCTURE, INTERACTION HETEROGENEITY AND DISPERSAL

A. Stabilising and destabilising mechanisms: explicit example

The focus of our work is the possibility of dispersal-induced instability. Ref. [S1] argues that dispersal can act to stabilise an equilibrium. In this section we comment on these seemingly contrasting findings, and we show that there is no contradiction.

The two mechanisms can in fact be thought of as quite separate. The stabilising effect reported in [S1] acts on bulk eigenvalues and is dependent upon spatial heterogeneity in the inter-species interactions. The destabilising effects of dispersal that we highlight, on the other hand, are due to outlier eigenvalues and require trophic structure in the community. As we will show, in a model which combines all of these features, both the stabilising and the destabilising effects of dispersal can be seen. Precisely which is observed depends on the circumstances.

We give an explicit demonstration of this in Fig. S13. The data in all four panels is obtained from a model combining spatial heterogeneity, trophic structure and dispersal. The different panels are for different choices of the model parameters. Panel (a) shows a spectrum for an equilibrium of a system with trophic structure, heterogeneity of interactions in space, but without dispersal. The example is such that the equilibrium is stable – the bulk eigenvalues and the outliers all have negative real parts. In panel (b) we now introduce dispersal, keeping all other model parameters unchanged. As seen in the figure, there are now outlier eigenvalues in the positive half-plane. The equilibrium has become unstable. The bulk eigenvalues however remain to the left of the real axis [with an overall tendency to move to the left compared to panel (a)]. The comparison of panels (a) and (b) therefore shows the destabilising effect of dispersal on outlier eigenvalues. This is the mechanism highlighted in the main text (see Fig. 2).

In Fig. S13(c) we show again an eigenvalue spectrum for an equilibrium of a system without dispersal. Parameters are chosen such that the bulk spectrum crosses the imaginary axis into the positive half-plane. This makes the equilibrium unstable. We note that outlier eigenvalues in this example have negative real parts. In panel (d) we now allow for dispersal, with all other parameters as in panel (c). The stabilising effect on the bulk eigenvalues sets in, and the bulk spectrum is now strictly in the negative half-plane. At the same time, the outlier eigenvalues also remain in the negative half. The equilibrium is now stable. This is the mechanism highlighted by GML [S1] (see also Section S11 B).

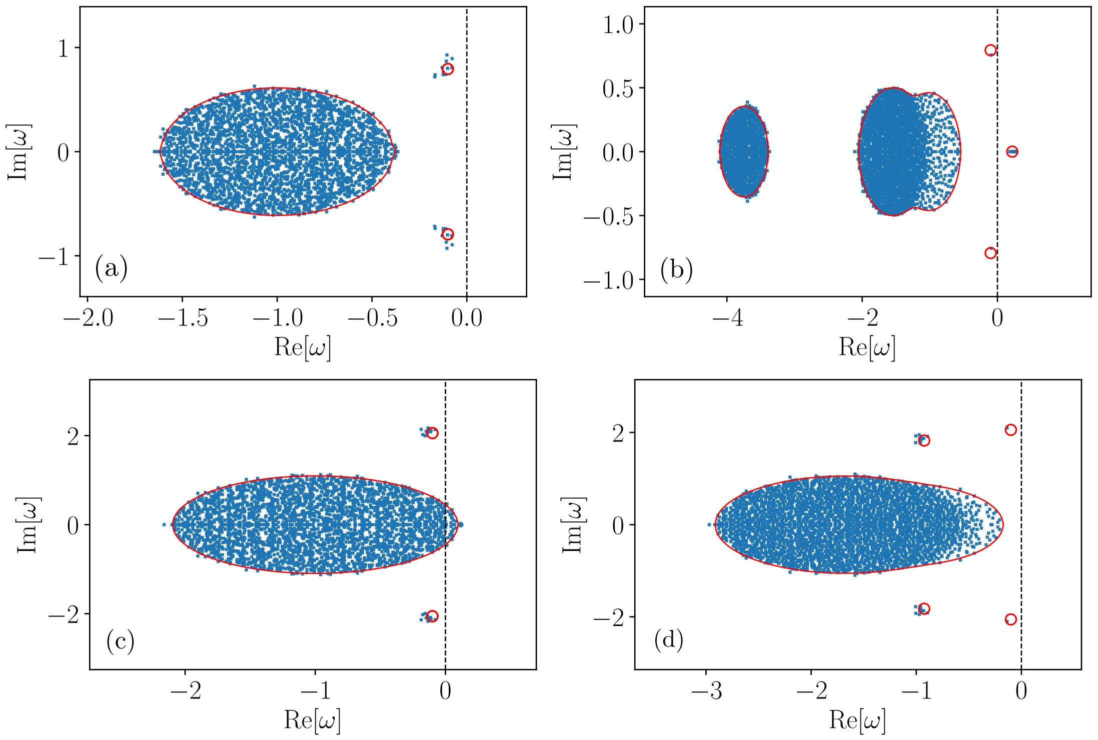

FIG. S13. **Demonstration of the stabilising and destabilising mechanisms in the same model.** Eigenvalue spectra demonstrating the different effects that result from the combination of trophic structure, spatial variation in interaction coefficients and dispersal. The combination of dispersal and spatial variation in interaction coefficients acts to stabilise whereas the combination of dispersal and trophic structure acts to destabilise. Depending on the system parameters, one effect takes precedence over the other. Panels (a) and (b) demonstrate the former effect and panels (c) and (d) demonstrate the latter. For the model parameters here (after rescaling, see Sec. S4), we have (a) $D_u = 0$, $D_v = 0$, $NC\mu_{vu} = -NC\mu_{uv} = 3.6$, $c = NC\sigma^2 = 0.325$ (b) $D_u = 0.5$, $D_v = 2.5$, $NC\mu_{vu} = -NC\mu_{uv} = 3.6$, $c = NC\sigma^2 = 0.325$ (c) $D_u = 0$, $D_u = 0$, $NC\mu_{vu} = -NC\mu_{uv} = 5.4$, $c = NC\sigma^2 = 1.21$ (c) $D_u = 0.5$, $D_v = 1$, $NC\mu_{vu} = -NC\mu_{uv} = 5.4$, $c = NC\sigma^2 = 1.2$. The remaining model parameters are $NC\mu_{uu} = 3.6$, $NC\mu_{uv} = -3.6$, $NC\mu_{vu} = 3.6$, $NC\mu_{vv} = -1.8$, $n = 11$, $\rho = 0.5$, $\Gamma_{uv} = 0$, $\Gamma_{uu} = 0$, $\Gamma_{vv} = 0$, $\gamma_u = 2/3$, $\gamma_v = 1/3$, $C = 1$ and $N = 300$.

B. Analytical demonstration

In order to take an analytically tractable example, we set $\Gamma_{\alpha\beta} = 0$ in Eqs. (S156) and once again use the dispersal and correlation kernels in Eq. (S171). Similar to the previous examples in Section S11 B, the first of Eqs. (S156) yields

$$\begin{aligned}&\sum_\alpha \gamma_\alpha \hat{f}_{\alpha;0}\left[1+\rho(n-1)\right] + \sum_\alpha \gamma_\alpha \hat{f}_{\alpha;-q}\left[\rho+n-1\right]\\&+\frac{\sigma^2}{n}\sum_{\alpha,\beta}\gamma_\alpha\gamma_\beta \hat{f}_{\alpha;0}\hat{f}_{\beta;-q}\left[(1-\rho)^2(n-1)-(1+\rho(n-1))(\rho+n-1)\right] = \frac{n}{\sigma^2},\end{aligned} \tag{S198}$$

subject to the following conditions

$$-w^\star_{\alpha;0}w_{\alpha;0} > 0, \quad -w^\star_{\alpha;0}w_{\alpha;0} \in \mathbb{R}, \quad -w^\star_{\alpha;q}w_{\alpha;q} > 0, \quad -w^\star_{\alpha;q}w_{\alpha;q} \in \mathbb{R},$$

$$1 - \frac{\sigma^2}{n}[1+\rho(n-1)]\sum_\alpha \gamma_\alpha \hat{f}_{\alpha;0} > 0, \quad 1 - \frac{\sigma^2}{n}[\rho+n-1]\sum_\alpha \gamma_\alpha \hat{f}_{\alpha;-q} > 0. \tag{S199}$$

Because $\Gamma_{\alpha\beta} = 0$, the equations for $w_{\alpha;q}$ and $w^\star_{\alpha;q}$ in Eq. (S156) decouple. When we solve these simultaneously with $\hat{f}_{\alpha;q} = -w^\star_{\alpha;q} w_{\alpha;q}$, we find

$$\hat{f}_{\alpha;-q} = \frac{1}{|\omega + d_\alpha + D_\alpha(1-\phi_q)|^2}. \tag{S200}$$

We thus obtain the following closed-form expression for the boundary of the eigenvalue support

$$\begin{aligned}&\sum_\alpha \gamma_\alpha \left[\frac{1+\rho(n-1)}{|\omega+d_\alpha|^2} + \frac{\rho+n-1}{|\omega+d_\alpha+\frac{D_\alpha n}{n-1}|^2}\right] \\ &+ \frac{\sigma^2}{n}\sum_{\alpha,\beta}\gamma_\alpha\gamma_\beta \frac{\left[(1-\rho)^2(n-1) - (1+\rho(n-1))(\rho+n-1)\right]}{|\omega+d_\alpha|^2|\omega+d_\beta+\frac{D_\beta n}{n-1}|^2} = \frac{n}{\sigma^2}.\end{aligned} \tag{S201}$$

For this simple example with $\Gamma_{\alpha\beta} = 0$, separate expressions for outlier eigenvalues associated with different values of q can be found from Eqs. (S165) and (S166). One arrives at the compact result

$$\left[\gamma_u\mu_{uu} - \lambda_q - d_u - D_u(1-\phi_q)\right]\left[\gamma_v\mu_{vv} - \lambda_q - d_v - D_v(1-\phi_q)\right] - \gamma_u\gamma_v\mu_{uv}\mu_{vu} = 0. \tag{S202}$$

This special case demonstrates that the stabilising and the destabilising mechanisms are rather separate. The region of the complex plane containing the bulk eigenvalues is determined by Eq. (S201). This relation has an explicit dependence on the level of spatial heterogeneity (parametrised by ρ), making heterogeneity a key factor for the stabilising influence of dispersal on the bulk eigenvalues. The outlier eigenvalues for this example on the other hand are determined by Eq. (S202), with no dependence on ρ (in a similar way to the outliers obtained in the single-trophic-level case in Section S12 C). This means that the outlier eigenvalues are not subject to the stabilising mechanism – the dispersal-induced instability can not be mitigated by spatial heterogeneity.

The picture becomes more complicated when there are correlations ($\Gamma_{\alpha\beta} \neq 0$). However, this example serves to illustrate our point which is that one must be wary of both the stabilising and destabilising effects. Dispersal is therefore not a unilaterally stabilising or destabilising force by itself. Rather, the picture is more subtle; in combination with spatial heterogeneity dispersal may be stabilising, given the correct circumstances, and in combination with trophic structure it may be destabilising.

C. Coexistence of the Turing instability and the heterogeneity-associated stabilisation

We have verified that the heterogeneity-associated stabilising mechanism can survive with the inclusion of trophic structure and that the Turing instability can survive with the inclusion of spatial heterogeneity in Fig. S13. Now, we investigate in a more general fashion under what conditions dispersal is a stabilising or a destabilising influence. This can be achieved by constructing a stability diagram similar to Fig. 3 in the main text (see Fig. S14) for the case where spatial heterogeneity is included. In general, the lines which divide the stable region of parameter space

from the unstable region can be found by identifying sets of parameters where (a) the bulk region crosses the imaginary axis or (b) an outlier crosses the imaginary axis.

To find the horizontal lines in Fig. S14, one solves Eqs. (S156) for the value of σ^2 where the boundary of the bulk eigenvalue support first touches the imaginary axis. Due to the symmetry of the support, this must occur at $\omega = 0$. That is, one solves the following equations for σ^2 [using the hopping and correlation kernels in Eqs. (S171)]

$$
\begin{aligned}
\det\left[V_{q,q'}\right] = 0, \quad V_{q,q'} = \delta_{q,q'} + \frac{\sigma^2}{n} c_{q-q'} \sum_{\alpha} \gamma_\alpha w^\star_{\alpha;q} w_{\alpha;q}, \\
0 = -1 + \left[i(d_\alpha + D_\alpha(1-\phi_q)) + \frac{\sigma^2}{n} \sum_{\beta,q'} \Gamma_{\alpha\beta}\gamma_\beta c_{q-q'} w^\star_{\beta;q'} \right] w^\star_{\alpha;q}, \\
0 = -1 + \left[i(d_\alpha + D_\alpha(1-\phi_q)) + \frac{\sigma^2}{n} \sum_{\beta} \Gamma_{\alpha\beta}\gamma_\beta c_{q-q'} w_{\beta;q'} \right] w_{\alpha;q}.
\end{aligned} \tag{S203}
$$

To find the sloped lines in Fig. S14, one instead solves Eqs. (S164) to (S166) for the value of σ^2, as a function of μ_{vu}, where an outlier first touches the imaginary axis. That is, one sets $\mathrm{Re}[\lambda] = 0$ in Eq. (S166). We leave $\mathrm{Im}[\lambda]$ as a free parameter but we note that for the Turing instability the imaginary part of the most unstable eigenvalue turns out to be zero in all cases that we tried. We note that after rescaling we have $\sigma^2 \to NC\sigma^2 = c$ and $\mu_{vu} \to NC\mu_{vu} = p$ (see Section S4).

Fig. S14 highlights which regions of parameter space are subject to the stabilising and the destabilising effects when dispersal is ‘switched on’. As with Fig. 3 of the main text, there remains a lower bound on the amount of predation required for stability – this lower bound is increased by the introduction of dispersal in a similar way to Fig. 3, hence destabilising certain parameters sets. There also remains an upper bound on the amount of complexity required for stability. Now, because of the inclusion of spatial heterogeneity, this upper bound is increased with dispersal and certain unstable parameter sets begin to exhibit stability. So when both trophic structure and spatial heterogeneity are included in the model, both the destabilising mechanism discussed in the main text and the stabilising mechanism discussed above play a role in determining how dispersal affects stability. Importantly, neither effect necessarily precludes nor diminishes the other.

D. What determines if dispersal is stabilising or destabilising?

We now explore the roles that the different components of the model play in determining whether one observes the stabilising or destabilising effects of dispersal. Because of the number of model parameters, it is difficult to be exhaustive. We therefore highlight the influence of the most salient components and focus on general tendencies.

The principal elements determining whether dispersal acts to stabilise or destabilise are: (i) the presence of a pronounced trophic structure; (ii) the ratio of the diffusion coefficients for predator and prey species; (iii) the overall magnitude of the dispersal; and (iv) spatial heterogeneity of the inter-species interactions. These different elements do not always act independently, and their interplay depends on other parameters, for example the complexity c. However, broadly speaking,

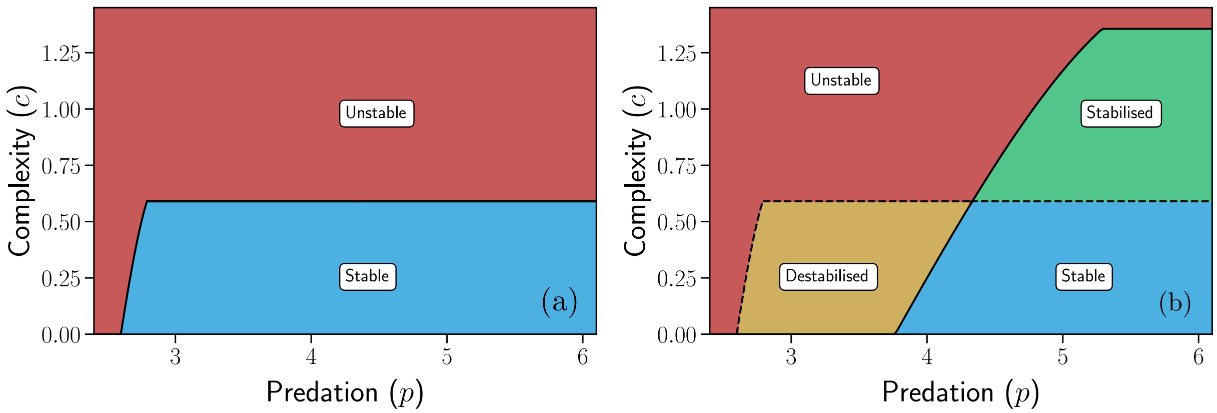

FIG. S14. If the interactions are spatially heterogeneous (species do not interact in the same way in one place as they do in another), stabilisation and destabilisation are both possible when dispersal is introduced. In a similar way to Fig. 3 of the main text, panel (a) shows sets of system parameters for which the model ecosystem is stable or unstable without taking dispersal into account. Panel (b) shows which sets of parameters are stable/unstable once dispersal is introduced. We have chosen a set of system parameters which accentuates both the stabilising and the destabilising mechanisms. The amount of spatial heterogeneity here is maximal ($\rho = 0$) and the ratio of the predator to the prey dispersal coefficients is quite large ($D_v/D_u = 10$). The remaining system parameters here are $n = 11$ (number of patches), $d_u = d_v = 1$, $\gamma_u = 2/3$, $\gamma_v = 1/3$, $\Gamma_u = 0.5$, $\Gamma_v = 0.6$, $\Gamma_{uv} = 0$, $D_u = 0.5$ and $D_v = 5$ (in panel (b)), $NC\mu_{uu} = 3$, $NC\mu_{vv} = -1.5$, and like Fig. 3 in the main text $NC\mu_{vu} = -NC\mu_{uv}$.

elements (i) and (ii) are relevant mostly for the the destabilising mechanism presented in the main text, whereas (iii) and (iv) relate more to the stabilising mechanism.

Elements (i) – (iv) listed above each correspond to a subset of the model parameters. For example, several parameters contribute to the degree of trophic structure in the population [item (i) above]. Among these are the relative sizes of the prey and predator blocks (γ_u, γ_v), the correlation parameters $\Gamma_{\alpha\beta}$, and the mean coefficients in each of the four blocks of the interaction matrix (i.e., the parameters $\mu_{\alpha\beta}$).

Elements (ii) and (iii) describe properties of the dispersal in the model. To discuss these quantitatively, it is useful to define $\theta = D_v/D_u$ as the predator-to-prey ratio of diffusion coefficients and $\bar{D} = \sqrt{D_u D_v}$ as the magnitude of dispersal. We then have $D_u = \bar{D}/\sqrt{\theta}$ and $D_v = \sqrt{\theta}\bar{D}$.

Finally, correlation (or, conversely, heterogeneity) of the interactions in space finally [item (iv)] is indicated by the model parameter ρ, with $\rho = 0$ representing the absence of any correlations (full heterogeneity), and $\rho = 1$ a situation in which there is no heterogeneity at all.

What promotes the dispersal-induced instability?

Similar to the simpler two-component systems in Turing's original work, an important characteristic for dispersal-induced instability in complex ecosystems is the ratio of the dispersal coefficients for predator and prey species. As shown in Fig. 4 of the main text, greater values of the predator-to-prey ratio of diffusion coefficients (θ) promote dispersal-induced instability. This can also be seen from Eq. (S100). Provided that there is dispersal, its magnitude $\bar{D}$ is not immediately im-

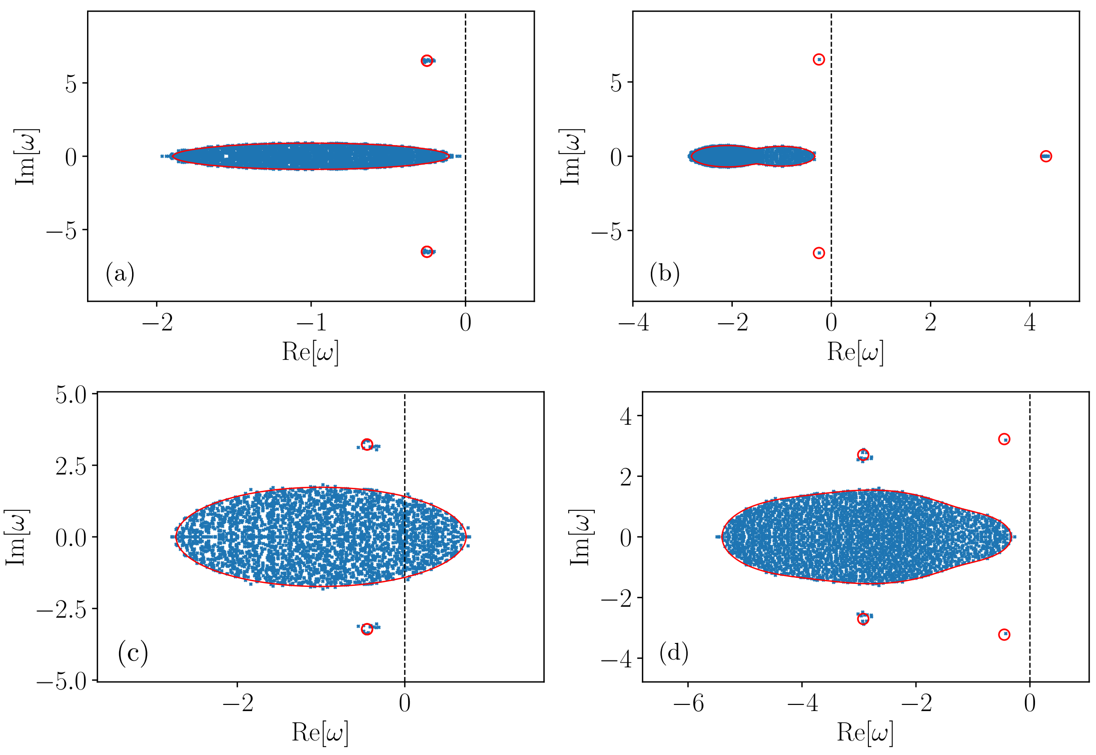

FIG. S15. **Exaggerated stabilising and destabilising mechanisms.** In comparison to Fig. S13 (a) and (b), panels (a) and (b) here demonstrate that an increased ratio D_v/D_u, increased trophic structure (reflected in $\mu_{\alpha\beta}$) and moderate complexity gives rise to a strong destabilising effect for dispersal. In comparison to Fig. S13 (c) and (d), panels (c) and (d) here demonstrate that a larger degree of spatial heterogeneity coupled with a sufficiently large typical dispersal rate $\bar{D}$ and large enough predation gives rise to a strong stabilising effect. For the model parameters here (after rescaling, see Sec. S4), we have (a) $D_u = 0$, $D_v = 0$, $c = NC\sigma^2 = 0.8$, $\rho = 0.5$, $NC\mu_{uu} = 15$, $NC\mu_{uv} = -24$, $NC\mu_{vu} = 24$, $NC\mu_{vv} = -25.5$; (b) the same but with $D_u = 1$, $D_v = 20$; (c) $D_u = 0$, $D_u = 0$, $c = NC\sigma^2 = 3$, $\rho = 0$, $NC\mu_{uu} = 3$, $NC\mu_{uv} = -7.5$, $NC\mu_{vu} = 7.5$, $NC\mu_{vv} = -2.4$ (d) the same but with $D_u = 1.5$, $D_v = 3$. The remaining model parameters are (in all cases) $n = 11$, $\Gamma_{uv} = 0$, $\Gamma_{uu} = 0$, $\Gamma_{vv} = 0$, $\gamma_u = 2/3$, $\gamma_v = 1/3$, $C = 1$ and $N = 300$.

portant for the instability in the mathematical model. The quantity $\bar{D}$ only appears as a factor multiplying q, so its main role is to control the wavelength associated with the instability.

As a second main component, trophic structure is required to see dispersal-induced instability (see ‘Eigenvalue spectra’ in Results in the main text). The structure of the community is determined by multiple parameters as discussed above. The role of each of these specific parameters is elucidated by the special cases in Section S5, but we here focus on predation p. In order for genuine dispersal-induced instability to occur the equilibrium must be stable when there is no dispersal. Fig. S14 (a) shows that this is only the case if p exceeds a threshold. Predation is also a stabilising influence in the system with dispersal, and dispersal-induced instability is not seen when predation is too high. As a result predation must be within a limited range in order for dispersal to destabilise equilibria which would otherwise be stable [see Fig. S14 (b)]. A competition between the ratio of diffusion coefficients (θ) and predation (p) then emerges. High values of θ promote dispersal-induced instability and increase the range of p for which such instability is possible.

The statements in the previous two paragraphs apply – in principle – to Turing's original two-species system [S36]. The parameter which best encapsulates how our model differs to a simple two-species system is the complexity $c = NC\sigma^2$. Complexity generally acts to destabilise equilibria in ecosystems with or without dispersal (as per May's original findings [S32]). Too high a complexity can therefore make the ecosystem unstable even if there is no dispersal [see Fig. S14(a)]. Therefore, complexity must be sufficiently low in order for dispersal to be the factor which destabilises the equilibrium [see Fig. S14 (b)]. After all, if the complexity is so large that the system without dispersal is unstable, then, of course, dispersal only has the potential to act as a stabilising influence. However, with this caveat in mind, an increase in complexity can also reduce the ratio of diffusion coefficients θ required for the Turing instability (Fig. 4 in the main text). Complexity then increases the propensity for dispersal to be destabilising.

In Fig. S15 (a) and (b) we exaggerate the destabilising effect [compared to Fig. S13 (a) and (b)] by increasing D_v/D_u, increasing the relative sizes of the trophic parameters $\mu_{\alpha\beta}$ and choosing a moderate complexity. We note that neither the presence nor the complete absence of spatial heterogeneity are necessary for dispersal-induced instability. The instability shown in Fig. 2 of the main paper for example is for a situation with identical interactions across space (no heterogeneity). Figs. S13, S14 and S15 demonstrate that dispersal-induced instability can persist in the face of heterogeneity.

What promotes the dispersal as a stabilising influence?

May's original observation [S32] was that for sufficiently high complexity c, the eigenvalues of a random Jacobian strayed into the real half-plane, signifying instability. The primary driving factor behind the stabilising mechanism first presented in Ref. [S1] is spatial heterogeneity of the interactions. The main mathematical effect of the combination of heterogeneity and dispersal is to compress a part of the bulk region of the eigenvalue spectrum, bringing the eigenvalues into the negative half-plane (see Fig. S8). Greater spatial heterogeneity gives rise to a greater amount of compression. This means that an unstable equilibrium can be stabilised by an increase of spatial heterogeneity.

In order to see this stabilising effect, spatial heterogeneity must be combined with a sufficiently large typical dispersal $\bar{D}$ (see Fig. S9). This is so that the part of the eigenvalue spectrum that is not subject to the shrinking effects of heterogeneity is moved sufficiently leftward. In a sense, dispersal in this context aids in allowing heterogeneity to be stabilising. We also note that the ratio of dispersal coefficients θ must not be too large so that both predator and prey species are subject to the stabilisation.

In order for an equilibrium to be stable, both the bulk of the eigenvalue spectrum and any outlier eigenvalues must be entirely in the negative half-plane. The stabilising effect of dispersal with heterogeneity acts primarily on the bulk eigenvalues. However, it does not apply in the same way to any outliers, which arise from the inclusion of trophic structure. It is these outliers which are responsible for the dispersal-induced instability. This means that dispersal can only stabilise an unstable equilibrium if no Turing instability is introduced by the dispersal.

In Fig. S15 (c) and (d), we demonstrate that the combination of increased spatial heterogeneity, a sufficiently large $\bar{D}$ and a large enough predation p (so as to preclude the excursion of positive

eigenvalues into the positive half-plane, see Fig. S14) acts to exaggerate the stabilising effect in comparison to Fig. S13 (c) and (d).

[S1] Dominique Gravel, François Massol, and Mathew A Leibold, "Stability and complexity in model meta-ecosystems," Nature Communications **7**, 12457 (2016).

[S2] Tobias Galla, "Random replicators with asymmetric couplings," Journal of Physics A: Mathematical and General **39**, 3853 (2006).

[S3] Guy Bunin, "Ecological communities with Lotka-Volterra dynamics," Physical Review E **95**, 042414 (2017).

[S4] Jacopo Grilli, Matteo Adorisio, Samir Suweis, György Barabás, Jayanth R Banavar, Stefano Allesina, and Amos Maritan, "Feasibility and coexistence of large ecological communities," Nature Communications **8**, 14389 (2017).

[S5] Stefano Allesina, Jacopo Grilli, György Barabás, Si Tang, Johnatan Aljadeff, and Amos Maritan, "Predicting the stability of large structured food webs," Nature Communications **6**, 1–6 (2015).

[S6] Joseph W. Baron and Tobias Galla, "Stochastic fluctuations and quasipattern formation in reaction-diffusion systems with anomalous transport," Physical Review E **99**, 052124 (2019).

[S7] Eugene P. Wigner, "Characteristic vectors of bordered matrices with infinite dimensions," Annals of Mathematics **62**, 548–564 (1955).

[S8] Eugene P Wigner, "On the distribution of the roots of certain symmetric matrices," Annals of Mathematics **67**, 325–328 (1958).

[S9] Brézin Edouard, Vladimir Kazakov, Didina Serban, Paul Wiegmann, and Anton Zabrin, eds., *Applications of Random Matrices in Physics* (Springer, Amsterdam, Netherlands, 2006).

[S10] Marc Mézard, Giorgio Parisi, and Miguel Virasoro, *Spin glass theory and beyond: An Introduction to the Replica Method and Its Applications*, Vol. 9 (World Scientific Publishing Company, London, 1987).

[S11] Madan Lal Mehta, *Random matrices*, Vol. 142 (Elsevier, London, 2004).

[S12] Leonid A Pastur, "On the spectrum of random matrices," Theoretical and Mathematical Physics **10**, 67–74 (1972).

[S13] Tim Rogers, "Universal sum and product rules for random matrices," Journal of Mathematical Physics **51**, 093304 (2010).

[S14] György Barabás, Matthew J Michalska-Smith, and Stefano Allesina, "Self-regulation and the stability of large ecological networks," Nature Ecology & Evolution **1**, 1870–1875 (2017).

[S15] Alan J. Bray and Michael A. Moore, "Evidence for massless modes in the 'solvable model' of a spin glass," Journal of Physics C: Solid State Physics **12**, L441–L448 (1979).

[S16] Pau Vilimelis Aceituno, Tim Rogers, and Henning Schomerus, "Universal hypotrochoidic law for random matrices with cyclic correlations," Physical Review E **100**, 010302 (2019).

[S17] Terence Tao and Van Vu, "Random matrices: Universality of local eigenvalue statistics up to the edge," Communications in Mathematical Physics **298**, 549–572 (2010).

[S18] Terence Tao, Van Vu, Manjunath Krishnapur, *et al.*, "Random matrices: Universality of ESDs and the circular law," The Annals of Probability **38**, 2023–2065 (2010).

[S19] Terence Tao, *Topics in Random Matrix Theory* (American Mathematical Society, Providence, Rhode Island, US, 2012).

[S20] Hans-Jürgen Sommers, Andrea Crisanti, Haim Sompolinsky, and Yaakov Stein, "Spectrum of large random asymmetric matrices," Physical Review Letters **60**, 1895–1898 (1988).

[S21] Alexander Altland and Ben D Simons, *Condensed Matter Field Theory* (Cambridge University Press, 2010).

[S22] Antonius C. C. Coolen, *The Mathematical Theory of Minority Games: Statistical Mechanics of Interacting Agents)* (Oxford University Press, Oxford, UK, 2005).

[S23] Elizabeth Gardner, "The space of interactions in neural network models," Journal of Physics A: Mathematical and Gegeneral **21**, 257 (1988).

[S24] Antonius C. C. Coolen, Reimer Kühn, and Peter Sollich, *Theory of Neural Information Processing Systems* (Oxford University Press, Oxford, UK, 2004).

[S25] Damien Challet, Matteo Marsili, and Yi-Chen Zhang, *Minority Games: Interacting Agents In Financial Markets* (Oxford University Press, Oxford, UK, 2005).

[S26] Fritz Haake, Felix Izrailev, Nils Lehmann, Dirk Saher, and Hans-Jürgen Sommers, "Statistics of complex levels of random matrices for decaying systems," Zeitschrift für Physik B Condensed Matter **88**, 359–370 (1992).

[S27] Sean O'Rourke, David Renfrew, *et al.*, "Low rank perturbations of large elliptic random matrices," Electronic Journal of Probability **19** (2014).

[S28] Stefano Allesina and Si Tang, "The stability–complexity relationship at age 40: a random matrix perspective," Population Ecology **57**, 63–75 (2015).

[S29] Samuel F Edwards and Raymund C Jones, "The eigenvalue spectrum of a large symmetric random matrix," Journal of Physics A: Mathematical and General **9**, 1595 (1976).

[S30] David Sherrington and Scott Kirkpatrick, "Solvable model of a spin-glass," Physical Review Letters **35**, 1792 (1975).

[S31] John Hubbard, "Calculation of partition functions," Physical Review Letters **3**, 77–78 (1959).

[S32] Robert M May, "Will a large complex system be stable?" Nature **238**, 413–414 (1972).

[S33] Stefano Allesina and Si Tang, "Stability criteria for complex ecosystems," Nature **483**, 205–208 (2012).

[S34] A. Yadav and Werner Horsthemke, "Kinetic equations for reaction-subdiffusion systems: Derivation and stability analysis," Physical Review E **74**, 066118 (2006).

[S35] Michael Cross and Pierre Hohenberg, "Pattern formation outside of equilibrium," Reviews of Modern Physiscs **65**, 851–1112 (1993).

[S36] Alan M Turing, "The chemical basis of morphogenesis," Philosophical Transactions of the Royal Society of London. Series B, Biological Sciences **237**, 37–72 (1952).
