# Supplementary material for: Dispersal-induced instability in complex ecosystems
Source: Nat Commun. 2020 Nov 27;11:6032. doi: 10.1038/s41467-020-19824-4 (PMC7695839; doi:10.1038/s41467-020-19824-4)
Supplement: Supplementary file 3 — Reporting Summary [file 41467_2020_19824_MOESM3_ESM.pdf]

nature research

Corresponding author(s): Tobias Galla

Last updated by author(s): 6 October 2020

Reporting Summary

Nature Research wishes to improve the reproducibility of the work that we publish. This form provides structure for consistency and transparency in reporting. For further information on Nature Research policies, see Authors & Referees and the Editorial Policy Checklist .

Statistics

For all statistical analyses, confirm that the following items are present in the figure legend, table legend, main text, or Methods section.

n/a	Confirmed	
[ ]	[x]	The exact sample size (n) for each experimental group/condition, given as a discrete number and unit of measurement
[ ]	[x]	A statement on whether measurements were taken from distinct samples or whether the same sample was measured repeatedly
[x]	[ ]	The statistical test(s) used AND whether they are one- or two-sided<br>*Only common tests should be described solely by name; describe more complex techniques in the Methods section.*
[ ]	[x]	A description of all covariates tested
[x]	[ ]	A description of any assumptions or corrections, such as tests of normality and adjustment for multiple comparisons
[x]	[ ]	A full description of the statistical parameters including central tendency (e.g. means) or other basic estimates (e.g. regression coefficient) AND variation (e.g. standard deviation) or associated estimates of uncertainty (e.g. confidence intervals)
[x]	[ ]	For null hypothesis testing, the test statistic (e.g. F, t, r) with confidence intervals, effect sizes, degrees of freedom and P value noted<br>*Give P values as exact values whenever suitable.*
[x]	[ ]	For Bayesian analysis, information on the choice of priors and Markov chain Monte Carlo settings
[x]	[ ]	For hierarchical and complex designs, identification of the appropriate level for tests and full reporting of outcomes
[x]	[ ]	Estimates of effect sizes (e.g. Cohen's d, Pearson's r), indicating how they were calculated

Our web collection on statistics for biologists contains articles on many of the points above.

Software and code

Policy information about availability of computer code

Data collection	We wrote custom codes in Wolfram Mathematica and Python to evaluate our theory predictions and to test these predictions against Monte Carlo simulations. Mathematica version 12 (commercial) and Python version 3.8 (open source) were used. Used Python packages included matplotlib, numpy and scipy. Codes for Figs. 2-6 are available from the following link https://doi.org/10.5281/zenodo.4068257. The codes for producing the figures in the Supplementary Material are available upon reasonable request.
Data analysis	We wrote custom codes in Wolfram Mathematica and Python to evaluate our theory predictions and to test these predictions against Monte Carlo simulations. Mathematica version 12 (commercial) and Python version 3.8 (open source) were used. Used Python packages included matplotlib, numpy and scipy. Codes for Figs. 2-6 are available from the following link https://doi.org/10.5281/zenodo.4068257. The codes for producing the figures in the Supplementary Material are available upon reasonable request.

For manuscripts utilizing custom algorithms or software that are central to the research but not yet described in published literature, software must be made available to editors/reviewers. We strongly encourage code deposition in a community repository (e.g. GitHub). See the Nature Research guidelines for submitting code & software for further information.

Data

Policy information about availability of data

All manuscripts must include a data availability statement. This statement should provide the following information, where applicable:

- Accession codes, unique identifiers, or web links for publicly available datasets
- A list of figures that have associated raw data
- A description of any restrictions on data availability

The data in Figs. 2-6 is generated using the codes in the code availability statement. The data is also available upon reasonable request to the corresponding author.

Field-specific reporting

Please select the one below that is the best fit for your research. If you are not sure, read the appropriate sections before making your selection.

☐ Life sciences ☐ Behavioural & social sciences ☒ Ecological, evolutionary & environmental sciences

For a reference copy of the document with all sections, see nature.com/documents/nr-reporting-summary-flat.pdf

Ecological, evolutionary & environmental sciences study design

All studies must disclose on these points even when the disclosure is negative.

Study description	We conduct a theoretical investigation into the role of dispersal in ecosystem stability. We modify May's random matrix approach to include multiple trophic levels and dispersive effects. Using mathematical techniques from Statistical Physics, we are able to predict how different aspects of an ecological community (complexity, predation, dispersal) contribute to stability.
Research sample	In order to verify our theoretical predictions of the eigenvalue spectra of random matrices, we generate realizations of random matrices and find their eigenvalues (using functions in the numpy Python library).
Sampling strategy	Different sizes of the random matrices were used to illustrate the range of validity of the theoretical approach (which is most accurate for large matrix dimension N).
Data collection	The data presented in the figures was produced using the codes at the following url: https://github.com/josephwbaron/Dispersal-induced-instability-in-complex-ecosystems<br>These were written by Joseph Baron and based on the theory detailed in the Supplemental Material.
Timing and spatial scale	N/A
Data exclusions	N/A
Reproducibility	We provide our codes at the following url: https://github.com/josephwbaron/Dispersal-induced-instability-in-complex-ecosystems
Randomization	N/A
Blinding	N/A

Did the study involve field work? ☐ Yes ☒ No

Reporting for specific materials, systems and methods

We require information from authors about some types of materials, experimental systems and methods used in many studies. Here, indicate whether each material, system or method listed is relevant to your study. If you are not sure if a list item applies to your research, read the appropriate section before selecting a response.

Materials & experimental systems

n/a	Involved in the study
☒	☐ Antibodies
☒	☐ Eukaryotic cell lines
☒	☐ Palaeontology
☒	☐ Animals and other organisms
☒	☐ Human research participants
☒	☐ Clinical data

Methods

n/a	Involved in the study
☒	☐ ChIP-seq
☒	☐ Flow cytometry
☒	☐ MRI-based neuroimaging
